# Supplementary material for: An Antifungal with a Novel Mechanism of Action Discovered via Resistance Gene-Guided Genome Mining
Source: ACS Cent Sci. 2026 Jan 31;12(2):197–207. doi: 10.1021/acscentsci.5c02019 (PMC12947280; doi:10.1021/acscentsci.5c02019)
Supplement: Supplementary file 1 [file oc5c02019_si_001.pdf]

Supporting Information for: An antifungal with a novel mechanism of action discovered via resistance-gene guided genome mining

Bruno Perlatti[1], Sandeep Vellanki[2], Yalong Zhang[3],Yi-Ming Chiang[1], Yingxia Hu[1], Mengdi Yuan[3], Kyle Dunbar[1], Abigail Fine[1], Michelle F Grau[1], Sheena Li[1], Timothy O'Donnell[1], Rajani Shenoy[1], Hongtao Li[5], Hui Shi[5],Xia Xu[5], Zeyu Chen[5], Tara Arvedson[1], Yi Tang[3,4], Robert A Cramer[2], Victor Cee[1], Colin JB Harvey[1]*

[1]Hexagon Bio, Menlo Park, California, 94025, USA

[2]Department of Microbiology and Immunology, Geisel School of Medicine, Dartmouth College, Hanover, New Hampshire, 03755, USA

[3]Department of Chemistry and Biochemistry and

[4]Department of Chemical and Biomolecular Engineering and University of California,Los Angeles, California, 90095, USA

[5]Biortus Biosciences Co. Ltd., China

*To whom correspondence should be addressed: colin@hexagonbio.com

Materials and Methods **6**
Media and buffer composition 6
Heterologous production of **1** in *Aspergillus nidulans* 6
Overexpression of the *sdt*BGC in *Aspergillus terreus* 7
Untargeted metabolomics analysis 7
Expression and purification of NeoA from *E. coli* 8
Expression and purification of microsomes containing NeoG from *S. cerevisiae* 8
Construction of a plasmid for expression of *neoG* in *S. cerevisiae* 9
In vitro characterization of the activities of NeoA and NeoG 9
ALS biochemical assay (*Sc*ALS) 10
ALS biochemical assay (*Af*ALS) 10
Expression and purification of *Sc*ALS 10
Expression and purification of *Af*ALS 11
Forward genetic screening of **1** 12
Antifungal screening of **1** 12
Protein expression and Purification for Cryo-EM 13
Cryo-EM sample preparation, data collection and processing 14
Model building and refinement 14
Construction of *A. fumigatus* AF293 *ilv2Δ* and the reconstituted strain 15
Assessing the pathogenicity of *A. fumigatus* AF293 variants in a mouse model of invasive aspergillosis 16
Spectroscopic Analyses 16
Isolation of compounds: 17
Isolation of compounds **1**, **13**, **14**, **15**, **16**, **17** and **19** 17
Isolation of compounds **11** and **12** 18
Structure elucidation of isolated compounds 18
Supporting Tables **20**
Table S1: Allele frequencies of all mutations observed in *S. cerevisiae* ALS from clones raised to be resistant to **1** 20
Table S2: Cryo-EM data collection, refinement and validation statistics 21
Table S3: Physical chemical and pharmacokinetic parameters for **1** 22
Table S4: MIC values for **1** against a panel of pathogenic molds. Values for both >50% and 100% growth inhibition measured at both 24 and 48 hours are provided 22
Table S5: Identity of residues shown to cause resistance across fungal species. Column titles are representative of the wild-type *S. cerevisiae* ALS 23
Table S6: Primers used in the construction and validation of *A. fumigatus* ilv2Δ (Afu3g10310) 24
Table S7: NMR spectroscopic data for **1** 25
Table S8: NMR spectroscopic data for **10** 26
Table S9: NMR spectroscopic data for **11** 27

Table S10: NMR spectroscopic data for **12**....28
Table S11: NMR spectroscopic data for **13**....29
Table S12: NMR spectroscopic data for **14**....30
Table S13: NMR spectroscopic data for **15**....31
Table S14: NMR spectroscopic data for **16**....32
Table S15: NMR spectroscopic data for **17**....33
Table S16: NMR spectroscopic data for **19**....34
Supporting Figures....35
Figure S1: Base peak chromatograms (BPCs) and extracted ion chromatograms for wild-type *A. terreus* and *A. terreus* P_{COX}-*sdtE*. 35
Figure S2: Detailed proposed biosynthesis of **1** inclusive of all isolated shunts and intermediates. 36
Figure S3: Sequence alignment of CghA and SdtD highlighting the A to S difference at position 242....37
Figure S4: Small molecule X-ray structure of **1**....37
Figure S5: Mutations in ALS observed in *S. cerevisiae* clones resistant to **1**. 38
Figure S6: Inhibition of recombinant *S. cerevisiae* ALS by **1** and its C12 diastereomer **13**.38
Figure S7: Peptide mapping of *S. cerevisiae* ALS treated with **1** localizes the site of covalent modification to the SGRPGPVLVDLPKDVTAAIL peptide. 39
Figure S8: cryoEM density of compound **1** and the conformational changes it causes at the AfALS substrate channel....40
Figure S9: Multiple sequence alignment of ALS proteins derived from all fungi included in the antifungal screening panel in this study. Positions where mutations were observed in forward genetic screens of **1** are highlighted with orange boxes....41
Figure S10: Validation of *A. fumigatus* mutants....42
Figure S11: SDS page gel of recombinant NeoA....43
Figure S12: Quality of the cryo-EM reconstruction of the Apo and **1**-bound *Af*ALS....43
Figure S13: The ^{1}H NMR Spectrum of **1** in MeOD (600 MHz)....44
Figure S14: The ^{13}C NMR Spectrum of **1** in MeOD (150 MHz)....44
Figure S15: The ^{1}H-^{1}H COSY NMR Spectrum of **1** in MeOD (600 MHz)....45
Figure S16: The ^{1}H-^{13}C HSQC NMR Spectrum of **1** in MeOD (600 MHz)....45
Figure S17: The ^{1}H-^{13}C HMBC NMR Spectrum of **1** in MeOD (600 MHz)....46
Figure S18: The ^{1}H-^{1}H NOESY NMR Spectrum of **1** in MeOD (600 MHz)....46
Figure S19: HRMS spectrum of **1**....47
Figure S20: The ^{1}H NMR Spectrum of **10** in MeOD (600 MHz)....47
Figure S21: The ^{13}C NMR Spectrum of **10** in MeOD (600 MHz)....48
Figure S22: The ^{1}H-^{1}H COSY NMR Spectrum of **10** in MeOD (600 MHz)....48
Figure S23: The ^{1}H-^{13}C HSQC NMR Spectrum of **10** in MeOD (600 MHz)....49
Figure S24: The ^{1}H-^{13}C HMBC NMR Spectrum of **10** in MeOD (600 MHz)....49
Figure S25: The ^{1}H-^{1}H NOESY NMR Spectrum of **10** in MeOD (600 MHz)....50
Figure S26: HRMS spectrum of **10**....50
Figure S27: The ^{1}H NMR Spectrum of **11** in $CDCN_3$ (500 MHz)....51

Figure S28: The ^{13}C NMR Spectrum of **11** in $CDCN_3$ (500 MHz).. 51
Figure S29: The ^{1}H-^{1}H COSY NMR Spectrum of **11** in $CDCN_3$ (500 MHz).......................... 52
Figure S30: The ^{1}H-^{13}C HSQC NMR Spectrum of **11** in $CDCN_3$ (500 MHz)......................... 52
Figure S31: The ^{1}H-^{13}C HMBC NMR Spectrum of **11** in $CDCN_3$ (500 MHz)......................... 53
Figure S32: The ^{1}H-^{1}H NOESY NMR Spectrum of **11** in $CDCN_3$ (500 MHz)........................ 53
Figure S33: HRMS Spectrum of **11**.. 54
Figure S34: The ^{1}H NMR Spectrum of **12** in CD_2Cl_2 (500 MHz).. 54
Figure S35: The ^{13}C NMR Spectrum of **12** in CD_2Cl_2 (500 MHz).. 55
Figure S36: The ^{1}H-^{1}H COSY NMR Spectrum of **12** in CD_2Cl_2(500 MHz)............................ 55
Figure S37: The ^{1}H-^{13}C HSQC NMR Spectrum of **12** in CD_2Cl_2 (500 MHz)......................... 56
Figure S38: The ^{1}H-^{13}C HMBC NMR Spectrum of **12** in CD_2Cl_2 (500 MHz)......................... 56
Figure S39: The ^{1}H-^{1}H NOESY NMR Spectrum of **12** in CD_2Cl_2 (500 MHz)........................ 57
Figure S40: HRMS spectrum of **12**.. 57
Figure S41: The ^{1}H NMR Spectrum of **13** in MeOD (600MHz).. 58
Figure S42: The ^{13}C NMR Spectrum of **13** in MeOD (150MHz)... 58
Figure S43: The ^{1}H-^{1}H COSY NMR Spectrum of **13** in MeOD (600 MHz)........................... 59
Figure S44: The ^{1}H-^{13}C HSQC NMR Spectrum of **13** in MeOD (600 MHz).......................... 59
Figure S45: The ^{1}H-^{13}C HMBC NMR Spectrum of **13** in MeOD (600 MHz).......................... 60
Figure S46: The ^{1}H-^{1}H NOESY NMR Spectrum of **13** in MeOD (600 MHz)......................... 60
Figure S47: HRMS Spectrum of **13**.. 61
Figure S48: The ^{1}H NMR Spectrum of **14** in MeOD (600MHz).. 61
Figure S49: The ^{13}C NMR Spectrum of **14** in MeOD (600MHz)... 62
Figure S50: The ^{1}H-^{1}H COSY NMR Spectrum of **14** in MeOD (600 MHz)........................... 62
Figure S51: The ^{1}H-^{13}C HSQC NMR Spectrum of **14** in MeOD (600 MHz).......................... 63
Figure S52: The ^{1}H-^{13}C HMBC NMR Spectrum of **14** in MeOD (600 MHz).......................... 63
Figure S53: The ^{1}H-^{1}H NOESY NMR Spectrum of **14** in MeOD (600 MHz)......................... 64
Figure S54: HRMS spectrum of **14**.. 64
Figure S55: The ^{1}H NMR Spectrum of **15** in MeOD (600MHz).. 65
Figure S56: The ^{13}C NMR Spectrum of **15** in MeOD (600MHz)... 65
Figure S57: The ^{1}H-^{1}H COSY NMR Spectrum of **15** in MeOD (600 MHz)........................... 66
Figure S58: The ^{1}H-^{13}C HSQC NMR Spectrum of **15** in MeOD (600 MHz).......................... 66
Figure S59: The ^{1}H-^{13}C HMBC NMR Spectrum of **15** in MeOD (600 MHz).......................... 67
Figure S60: The ^{1}H-^{1}H NOESY NMR Spectrum of **15** in MeOD (600 MHz)......................... 67
Figure S61: HRMS spectrum of **15**.. 68
Figure S62: The ^{1}H NMR Spectrum of **16** in MeOD (600MHz).. 68
Figure S63: The ^{13}C NMR Spectrum of **16** in MeOD (600MHz)... 69
Figure S64: The ^{1}H-^{1}H COSY NMR Spectrum of **16** in MeOD (600 MHz)........................... 69
Figure S65: The ^{1}H-^{13}C HSQC NMR Spectrum of **16** in MeOD (600 MHz).......................... 70
Figure S66: The ^{1}H-^{13}C HMBC NMR Spectrum of **16** in MeOD (600 MHz).......................... 70
Figure S67: The ^{1}H-^{1}H NOESY NMR Spectrum of **16** in MeOD (600 MHz)......................... 71
Figure S68: HRMS spectrum of **16**.. 71

Figure S69: The ^{1}H NMR Spectrum of **17** in MeOD (600MHz)............72
Figure S70: The ^{13}C NMR Spectrum of **17** in MeOD (600MHz)............72
Figure S71: The ^{1}H-^{1}H COSY NMR Spectrum of **17** in MeOD (600 MHz)............73
Figure S72: The ^{1}H-^{13}C HSQC NMR Spectrum of **17** in MeOD (600 MHz)............73
Figure S73: The ^{1}H-^{13}C HMBC NMR Spectrum of **17** in MeOD (600 MHz)............74
Figure S74: The ^{1}H-^{1}H NOESY NMR Spectrum of **17** in MeOD (600 MHz)............74
Figure S75: HRMS spectrum of **17**............75
Figure S76: The ^{1}H NMR Spectrum of **19** in MeOD (600MHz)............75
Figure S77: The ^{13}C NMR Spectrum of **19** in MeOD (150MHz)............76
Figure S78: The ^{1}H-^{1}H COSY NMR Spectrum of **19** in MeOD (600 MHz)............76
Figure S79: The ^{1}H-^{13}C HSQC NMR Spectrum of **19** in MeOD (600 MHz)............77
Figure S80: The ^{1}H-^{13}C HMBC NMR Spectrum of **19** in MeOD (600 MHz)............77
Figure S81: The ^{1}H-^{1}H NOESY NMR Spectrum of **19** in MeOD (600 MHz)............78
Figure S82: HRMS spectrum of **19**............78
References............79

Materials and Methods

Media and buffer composition

ST buffer: 1.2 M sorbitol, 12.5 mM Tris-HCl, pH 7.5

STC buffer: 1.2 M sorbitol, 10 mM calcium chloride, 12.5 mM Tris-HCl, pH 7.5

Digestion buffer: 280 g/l $MgSO_4 \cdot 7H_2O$, 120 g/l Vino Taste Pro, in a 175 mM sodium phosphate buffer containing 14.79 g/l $NaH_2PO_4 \cdot H_2O$, 17 g/l Na_2PO_4, with pH adjusted to 7.0

GMM: 10 g/L glucose, 6 g/L $NaNO_3$, 0.52 g/L KCl, 0.52 g/L $MgSO_4 \cdot 7H_2O$, 1.52 g/L KH_2PO_4, 1 mL/L trace elements concentrate, 0.05 w/v Riboflavin

YG: 20 g/l glucose, 10 g/l yeast extract, and 1 mL trace elements concentrate

20X salt solution: 120 g/l $NaNO_3$, 10.4 g/l KCl, 10.4 g/l $MgSO_4 \bullet 7H_2O$, 30.4 g/l KH_2PO_4

SMK: 40 g/L soluble starch, 1 g/L yeast extract, 4.3 g/L Murashige and Skoog Basal Salt Mixture

Trace elements concentrate: 1.0 g/L $FeSO_4.7H_2O$, 1.0 g/L $MnSO_4.H_2O$, 0.2 g/L $ZnSO_4.7H_2O$, 0.1 g/L $CaCl_2.2H_2O$, 0.056 g/L HBO_3, 0.025 g/L $CuCl_2.2H_2O$, 0.019 g/L $(NH_4)6.Mo_7O_{24}.4H_2O$, 50 mL/L 12M HCl)

YPD medium: 20 g/L peptone, 10 g/L yeast extract, 10 g/L dextrose

TEGM buffer: 50 mM Tris-HCl, pH 7.5, 1 mM EDTA, 0.6 M sorbitol, 1.5 mM β-mercaptoethanol, 20% glycerol (v/v), Pierce^{TM} protease inhibitor

Formulation buffer: 300 mM K_2HPO_4, pH 8, 10 μM FAD, 0.5 mM TCEP, 10% glycerol v/v

Heterologous production of 1 in *Aspergillus nidulans*

To produce the metabolites encoded in the *sdt*BGC, we refactored the BGC in *A. nidulans* using the asperfuraone(*afo*) regulon with the gene for the positive regulator AN1029 (*afoA*) expressed under the inducible alcA promoter (P_{alcA}) as previously described.[1] In brief, intergenic regions of the *afo* regulon were amplified by PCR from the gDNA of *A. nidulans* and 7 genes of the *sdt*BGC were ordered as synthetic DNA from Twist Bioscience and PCR amplified. All relevant amplicons were gel purified and put through intermediate assemblies using NEBuilder HiFi DNA Assembly Master Mix (NEB, #E2621). The assembled DNA fragments were then used as templates for PCR to generate seven large amplicons, ranging in size from 4.2 to 8.5 kb for transformation. The recipient strain was derived from *A. nidulans* A1145 (the Fungal Genetics Stock Center strain number, https://www.fgsc.net/), in which the BGCs for sterigmatocystin (AN7804 – AN7825) and asperfuranone (AN1036 – AN1028) have been deleted. Protoplast preparation and *A. nidulans* transformation of the 7 prepared fragments were carried out as previously described.[2] After transformation, prototrophic colonies were randomly picked and examined by diagnostic PCR.

For each transformation, 6 individual clones were picked and inoculated into each of two deep-well blocks, one with 750 µl of glucose minimal media (GMM) in each well and the second with 750 µl lactose containing minimal media (LMM) per well. All cultures were grown for 48 hours at 30°C with shaking at 750 RPM prior to induction by the addition of 3.75 µl (0.5 % v/v) methylethyl ketone. After a further 72 hrs of growth under the same conditions, all plates were frozen and lyophilized overnight. Lyophilized culture solids were resuspended in 750 µl of methanol, left to soak for 2 hours and filtered through 2 µM filter plates prior to LCMS analysis.

Overexpression of the *sdt*BGC in *Aspergillus terreus*

The cluster specific transcription factor gene *sdtE* was cloned under the control of the P_{COX} promoter from the *A. nidulans* cytochrome C oxidase and the terminator from gene AN0717 on a plasmid containing an AMA1 origin of replication and a hygromycin resistance marker. 2 µg of the assembled plasmid was used for each transformation.

To prepare biomass for transformation, *Aspergillus pseudoterreus* (NRRL4017, isolated from a soil sample taken in Argentina) was cultured for 24 hr at 180 rpm in 100 mL of YG media. Mycelia were collected from this culture and prepared for transformation as described previously[2]. Briefly, mycelia were incubated at 30°C and 100 rpm for 4-5 hr in 100 mL of freshly prepared and filtered digestion buffer. 45 mL of this protoplast suspension was filtered through miracloth, and 5 mL of 0.4 M ST buffer was gently layered on top. Samples were centrifuged at 1800 G for 10 min with deceleration on the gentlest setting. Protoplasts were collected at the interface and washed once with 0.6M KCl solution containing 44.7 g/l KCl, and once with 0.6M KCl, $CaCl_2$ solution containing (g/L): KCl, 44.7; $CaCl_2$ 7.4. Protoplasts were quantified and diluted to 1.0×10^8 protoplasts/ mL with KCl, $CaCl_2$ solution. Transformation of the prepared protoplasts was undertaken with 2 µg of plasmid DNA as previously described.[2] Each transformation was added to 35 mL of molten minimal media + KCl + hygromycin containing: 15 g/l glucose, 44.7 g/l KCl, 8 g/l agar, 0.4 g/l hygromycin, 1 mL trace elements concentrate, and 50 mL of 20 X salt solution. This mixture was gently inverted and poured into a 10 cm petri dish. Transformations were incubated at 37°C for 3-5 days at which point colonies were picked, cultured as described above, and subjected to untargeted metabolomic analysis.

Untargeted metabolomics analysis

High-resolution LCMS analyses were performed on a Thermo Fisher Scientific Vanquish Horizon UHPLC System integrated with a Thermo Q Exactive hybrid quadrupole-orbitrap high-resolution mass spectrometer with a HESI ion source. Two µL of the filtered culture extract to be analysed was injected and separated using a water-acetonitrile gradient on an Accucore Vanquish C18+ Reversed Phase HPLC Column (1.5 µm, 2.1 mm x 50 mm, Cat# 27101-052130) maintained at 50°C. Solvent A: 0.1% formic acid in water; Solvent B: 0.1% formic acid in acetonitrile. A/B gradient started at 12.5% B increased linearly to 97% B at 8.5 min and held at 97% B for 1 min, using a flow rate 0.6 mL/min. Mass spectrometer parameters: spray voltage 3 kV for positive mode, capillary temperature 380 °C, probe heater temperature 400 °C; sheath, auxiliary, and spare gas 60, 20, and 1, respectively; S-lens RF level 50, resolution 140,000 at m/z 200, AGC target 3×10^{10}. Tandem mass spectrum analysis was carried out with an exclusion list of known endogenous features with the same parameters (vide supra) with the following

additions or adjustments: Full MS Resolution: 35,000, AGC target 1 x 10^6, Max IT: 30 ms, scan range: 150 to 1400 m/z, ddMS2 Resolution: 17,500, AGC target 5 x 10^4, Max IT: 50 ms, Loop Count: 10, isolation window: 2.0 m/z, stepped collision energy: 20, 40, 60 NCE.

Untargeted LCMS metabolomics data were analyzed using an internally developed, proprietary algorithm (FTOP; Hexagon Bio), which is not publicly available. The algorithm operates on a reference corpus comprising LC–MS features extracted from approximately 10,000 high-resolution LC–MS runs of *Aspergillus nidulans* chassis strains engineered for heterologous expression of ~400 distinct fungal BGCs. For each detected feature, defined by an m/z value and retention time, the algorithm estimates the likelihood that the feature originates from a specific target BGC (*sdt*BGC) based on its frequency of occurrence across the full dataset. Feature matching across runs was performed using an m/z tolerance of 5 ppm and a retention time tolerance of 0.03 min. Features that occur preferentially in samples expressing the *sdt*BGC relative to the broader dataset are assigned higher scores. While the full implementation of the algorithm is proprietary, the methodological framework described here enables evaluation of its application and limitations.

Expression and purification of NeoA from *E. coli*

The *neoA* gene was amplified with overhang primers from the cDNA of strain *A. nidulans* expressing *neoABDFGH*. The PCR product was cloned into pET-28a(+) digested with NdeI/BamHI using NEBuilder® HiFi DNA Assembly Master Mix (NEB, #E2621). The resulting plasmid was confirmed by DNA sequencing and introduced into *E. coli* BL21(DE3) cells for His_6-tagged protein induction and purification. The *E. coli* cells harboring the corresponding plasmid were grown overnight in LB medium with 50 μg/mL kanamycin at 37°C, 220 rpm. 5 mL of the starter culture was inoculated into 1 L of fresh LB medium with 50 μg/mL kanamycin and incubated at 37°C until the optical density at 600 nm (OD_{600} value) reached 0.6. After the culture was submerged in ice water for 25 min, protein expression was induced by addition of 100 μM isopropyl-β-D-thiogalactopyranoside (IPTG) and cultured for an additional 20 hrs at 16°C, 220 rpm. Cells were subsequently harvested by centrifugation at 5,000 rpm, 4°C for 10 min, resuspended in 40 mL lysis buffer (50 mM Tris-HCl, pH 7.5, 10 mM imidazole, 500 mM NaCl and 10% glycerol, v/v), and lysed by sonication on ice. The lysate was centrifuged at 14,000 rpm, 4°C for 30 min to remove the cellular debris. Then the supernatant was subjected to Ni-NTA affinity chromatography at 4°C for protein purification according to the manufacturer's protocols (GE Healthcare). The purified protein was concentrated and exchanged into a storage buffer (50 mM Tris-HCl, 50 mM NaCl, pH 7.5) by using Amicon® Ultra-15 Centrifugal Filters. The purity of protein was checked by SDS-PAGE (**Figure S11**), and the concentration was determined by Bradford method. The purified proteins were flash-frozen in liquid N_2 and stored at -80°C.

Expression and purification of microsomes containing NeoG from *S. cerevisiae*

S. cerevisiae RC01 transformed with a plasmid expressing *neoG* was cultivated in 5 mL selective dropout medium at 28°C for 20 h. 4 mL of the starter culture was inoculated into YPD medium for an additional 48 h at 28°C, 220 rpm. The cells were harvested by centrifugation at

4,300 rpm, 4°C for 5 min, and the cell pellet was resuspended in 4 mL TEGM buffer. Glass beads (0.5 mm in diameter) were added and the cell walls were disrupted by vortexing for 1 min separated by 1 min intervals on ice for a total of 12 min. Then the microsomal fractions were obtained as described previously.[3] The microsomal fractions were resuspended in 50 mM Tris-HCl (pH 7.5, 50 mM NaCl, 10% glycerol, v/v).

Construction of a plasmid for expression of *neoG* in *S. cerevisiae*

To construct the plasmid for neoG expression in Saccharomyces cerevisiae, the vector XW551,[4] containing a uracil auxotrophic selection marker, was first digested with NheI and NdeI. *neoG* was amplified using primers containing overlapping sequences from the cDNA of strain AN-*neoABDFGH*. The digested vector and amplified *neoG* fragment were assembled using NEBuilder® HiFi DNA Assembly Master Mix (New England Biolabs) and subsequently transformed into *E. coli* TOP10 for plasmid propagation. Plasmids were isolated from *E. coli* using the Zyppy™ Plasmid Miniprep Kit (Zymo Research) and verified by restriction enzyme digestion and DNA sequencing. The resulting construct, XW551-neoG, in which *neoG* expression is driven by the ADH2 promoter, was then transformed into *S. cerevisiae* strain RC01.

In vitro characterization of the activities of NeoA and NeoG

Enzymatic assays of NeoA were performed in 50 mM NaPB (pH 7.6) with a final volume of 100 μL containing 10 μM NeoA with 250 μM compound **11** and 10 mM NADPH. The reactions were incubated at 28°C overnight and then quenched with 300 μL CH_3CN. The mixtures were centrifuged at 17,000 g for 5 min. LCMS analysis of clarified supernatants was performed on a Shimadzu 2020 EV LC–MS with a reversed-phase column (Phenomenex Kinetex 1.7 μm C18 100 Å, LC Column 100 × 2.1 mm) using positive-and negative-mode electrospray ionization with a linear gradient of 5–95% CH_3CN-H_2O with 0.1% FA in 15 min followed by 95% CH_3CN for 3 min with a flow rate of 0.3 ml/min.

Assays of microsomes containing NeoG were performed in 50 mM NaPB (pH 7.6) with a final volume of 500 μL containing 50 μL microsomal fractions with 50 μM **12** and 500 μM NADPH. The reactions were incubated at 28°C for 13 h and extracted with 550 μL EtOAc. The EtOAc extracts were evaporated to dryness and then redissolved in methanol for LC-MS analysis. The samples were analyzed by an Agilent 1260 Infinity II LC equipped with an InfinityLab Poroshell 120 EC-C18 column (2.7 μm, 3.0 × 50 mm) and a 6545 QTOF high resolution mass spectrometer (Dual AJS ESI ion source - positive mode, Vcap voltage 3.5kV, nozzle voltage 1kV, gas and sheath gas temperature 350 ℃, drying and sheath gas flow 11 L/min, nebulizer 60 psig. MS TOF - fragmentor, skimmer and Oct 1 RF Vpp voltages 175, 65 and 750 V respectively, m/z range 100-3000 m/z at 1 spectra/s, 9949 transients/spectrum, resolution 40.000 at m/z 2722) using the following gradient: 1% CH_3CN-H_2O 2 min, then 1−95% CH_3CN-H_2O (both with 0.1% formic acid (FA), v/v) in 9 min followed by 95% CH_3CN-H_2O for 6 min at a flow rate of 0.8 mL/min.

ALS biochemical assay (*Sc*ALS)

Saccharomyces cerevisiae acetohydroxyacid synthase (*Sc*ALS) lacking the N-terminal mitochondrial-targeting sequence (amino acids 58-687) was overproduced and isolated from *Escherichia coli* as an N-terminally His_6-tagged protein. Assays (50 µL final volume) contained 100 nM His_6-*Sc*ILV2 (58-687), 8 mM pyruvate, 1 mM thiamine pyrophosphate, and 10 µM flavin adenine dinucleotide, 10 mM MgCl2, 1 mM DTT, 1% DMSO (± inhibitor) in 500 mM K_2HPO_4 buffer at pH 7.5. Following a 30 min pre-incubation of the enzyme and inhibitor in the reaction mixture at room temperature, reactions were initiated with the addition of pyruvate. *Sc*ALS assays were carried out at 30˚C for 60 min. The reaction was quenched with the addition of 5 µL of 10% H_2SO_4 (v/v in deionized water). Reactions were incubated at 60˚C at 800 rpm in an incubating microplate shaker for 15 min to convert the product of the ALS reaction, acetolactate, to acetoin. Subsequently, the concentration of acetoin was determined by the addition of 7.5 µL 6 M NaOH, 25 µL 0.5% creatine (w/v in deionized water), and 25 µL 4% 1-naphthol (w/v in 2.5 M NaOH). This mixture was incubated at 60˚C at 800 rpm in an incubating microplate shaker for 15 min. 40 µL of this reaction mixture was transferred to a half area UV-star 96-well plate (Grenier) and the absorbance at 525 nm was measured on a Spark multimode plate reader (Tecan). The percent inhibition was calculated by the comparison of the signal to DMSO (0% inhibition) and no enzyme (100% inhibition) controls. IC_{50} values were calculated from the dose response curves using Prism 9 (four parameter, variable slope).

ALS biochemical assay (*Af*ALS)

Aspergillus fumigatus acetohydroxyacid synthase (*Af*ILV2) lacking the N-terminal mitochondrial-targeting sequence (amino acids 112-752) was overproduced and isolated from *E. coli* as an N-terminally His_6-tagged protein. Assays (50 µL final volume) contained 200 nM His_6-*Af*ALS, 8 mM pyruvate, 1 mM thiamine pyrophosphate, and 10 µM flavin adenine dinucleotide, 10 mM MgCl2, 1 mM DTT, 1% DMSO (± inhibitor) in 200 mM KH2PO4 buffer at pH 7.5. Following a 30 min pre-incubation of the enzyme and inhibitor in the reaction mixture at room temperature, reactions were initiated with the addition of pyruvate. *Af*ALS assays were carried out at 30˚C for 120 min. The reaction was quenched with the addition of 5 µL of 10% H_2SO_4 (v/v in deionized water). Reactions were incubated at 60˚C at incubating microplate shaker for 15 min to convert the product of the ALS reaction, acetolactate, to acetoin. Subsequently, the concentration of acetoin was determined by the addition of 7.5 µL 6 M NaOH, 25 µL 0.5% creatine (w/v in deionized water), and 25 µL 4% 1-naphthol (w/v in 2.5 M NaOH). This mixture was incubated at 60˚C in an incubating microplate shaker for 15 min. 40 µL of this reaction mixture was transferred to a half area UV-star 96-well plate (Grenier) and the absorbance at 525 nm was measured on a Spark multimode plate reader (Tecan). The percent inhibition was calculated by the comparison of the signal to DMSO (0% inhibition) and no enzyme (100% inhibition) controls. IC_{50} values were calculated from the dose response curves using Prism 9 (four parameter, variable slope).

Expression and purification of *Sc*ALS

S. cerevisiae ILV2 (the gene or *Sc*ALS) lacking the N-terminal mitochondrial-localization tag (amino acids 58-687) was codon optimized for expression in *E. coli* and synthesized by

Genscript. The gene was cloned into a pET28a vector to afford a TEV-protease cleavable N-terminal His_6-tagged translational fusion protein. *E. coli* BL21 (DE3) cells were transformed with pET28a-ScILV2 and transformants were selected on LB agar plates supplemented with 50 µg/mL kanamycin. A single colony was used to inoculate 7 mL of LB supplemented with kanamycin and cultures were grown for 18-20 h at 37 ˚C and 180 rpm. The entire overnight culture was used to inoculate 750 mL of kanamycin-supplemented LB in baffled 2 L flasks and cultures were grown at 37˚C and 180 rpm until OD_{600} reached 0.6. Cultures were then placed on ice for 15 min before *Sc*ILV2 expression was induced with the addition of 0.4 mM IPTG. Cultures were grown for an additional 18 h before cells were harvested by centrifugation at 4000 x g for 15 min. Cell pellets were resuspended in 35 mL of ice cold lysis buffer (50 mM Tris pH 7.5, 500 mM NaCl, 5% glycerol, 5 mM imidazole) supplemented with 1 mg/mL lysozyme and complete EDTA-free protease inhibitor cocktail (Roche). Cell suspensions were lysed by sonication for 5 min (2 sec on, 4 sec off; 35% power) using a Qsonica sonicator and insoluble debris was removed by centrifugation at 20,000 x g for 30 min at 4˚C. The soluble fraction was applied to 2 mL of TALON fast flow resin (Cytiva) pre-equilibrated with lysis buffer. The resin was washed with 20 column volumes of lysis buffer and bound protein was eluted in 30 mL of elution buffer (200 mM KH_2PO_4 pH7.5, 10 µM FAD, 0.5 mM TCEP, 125 mM imidazole, 10% glycerol). Protein was concentrated in a 30 kDa MWCO Amicon centrifugal filter (Millipore) and subjected to size exclusion chromatography with a superdex 16/600 200 pg column (Cytiva) equilibrated with 200 mM KH_2PO_4 pH 7.5, 10 µM FAD, 0.5 mM TCEP, 10% glycerol. Fractions containing *Sc*ALS were pooled and concentrated to 10-20 mg/mL in a 30 kDa MWCO Amicon centrifugal filter (Millipore). Purity was assessed by coomassie-stained SDS-PAGE and protein concentration was determined by absorbance at 280 nm and Bradford.

Expression and purification of *Af*ALS

A fumigatus ILV2 (the gene for *Af*ALS) lacking the N-terminal mitochondrial-localization tag (amino acids 112-752) was codon optimized for expression in *E. coli* and synthesized by Genscript. The gene was cloned into a pET28a vector to afford a TEV-protease cleavable N-terminal His_6-tagged translational fusion protein. *E. coli* BL21 (DE3) cells were transformed with pET28a-*Af*ILV2 and transformants were selected on LB agar plates supplemented with 50 µg/mL kanamycin. A single colony was used to inoculate 50 mL of LB supplemented with kanamycin and cultures were grown for 18-20 h at 37˚C and 180 rpm. The entire overnight culture was used to inoculate 1 L of kanamycin-supplemented TB in baffled 2 L flasks and cultures were grown at 37˚C and 180 rpm until OD_{600} reached 1. Cultures were then placed on ice for 15 min before *Af*ALS expression was induced with the addition of 0.3 mM IPTG. Cultures were grown for an additional 20 h before cells were harvested by centrifugation at 4000 x g for 15 min. Cell pellets were resuspended in 35 mL of ice cold lysis buffer (50 mM K_2HPO_4, pH 8.0, 300 mM NaCl, 5 mM imidazole, 10 µM FAD) supplemented with 1 mg/mL lysozyme and complete EDTA-free protease inhibitor cocktail (Roche). Cell suspensions were lysed by sonication for 10 min (2 sec on, 5 sec off; 45% power) using a Qsonica sonicator and insoluble debris was removed by centrifugation at 20,000 x g for 30 min at 4 ˚C. The soluble fraction was applied to 2 mL of TALON fast flow resin (Cytiva) pre-equilibrated with lysis buffer. The resin was washed with 20 column volumes of lysis buffer and bound protein was eluted in 30 mL of elution buffer (500 mM K_2HPO_4, pH 8, 10 µM FAD, 0.5 mM TCEP, 300 mM imidazole, 10%

glycerol). Protein was concentrated in a 30 kDa MWCO Amicon centrifugal filter (Millipore) and subjected to size exclusion chromatography with a superdex 16/600 200 pg column (Cytiva) equilibrated with 300 mM K_2HPO_4, pH 8, 10 µM FAD, 0.5 mM TCEP. Fractions containing *Af*ALS were pooled and concentrated to 10-20 mg/mL in a 30 kDa MWCO Amicon centrifugal filter (Millipore). Purity was assessed by coomassie-stained SDS-PAGE and protein concentration was determined by absorbance at 280 nm and Bradford.

Forward genetic screening of 1

Ethyl methanesulfonate (EMS) mutagenesis was performed on *S. cerevisiae* to facilitate the raising of resistant clones. Briefly, overnight yeast cultures were grown in YPD broth at 30°C with shaking at 180 rpm. Cells were harvested by centrifugation, washed once with sterile water and twice with 0.1 M NaH_2PO_4, and 10^8 cells added to 1.5 mL eppendorf tubes containing 1.7 mL 0.1 M NaH_2PO_4. Mutagenesis was initiated by addition of 25 µL EMS to each tube followed by incubation for 1 h at 30°C with agitation, after which the reaction was quenched with 8 mL 5% sodium thiosulfate. Mutagenized cells were washed and resuspended in sterile water to prepare for spreading on selection plates.

Selection plates were prepared by spreading 30 µL of DMSO containing increasing concentrations of **1** dispensed into each well of a 6-well agar plate. Concentrations tested were 5 µM, 10 µM, 20 µM, 40 µM, and 80 µM. The solution was spread evenly over the wells and plates were dried overnight. Mutagenized cells were then plated onto drug-containing agar and incubated at 30°C for 4–5 days until resistant colonies appeared.

Antifungal screening of 1

Antifungal screening was performed by Micromyx, LLC (Kalamazoo, Michigan, USA). Test organisms were either reference strains from the American Type Culture Collection (ATCC; Manassas, VA), the Centers for Disease Control and Prevention (CDC; Atlanta, GA), or clinical isolates from the Micromyx (MMX) collection. Upon receipt at Micromyx, the isolates were streaked under suitable conditions onto agar medium appropriate to each organism. The yeast isolates were incubated for 24 hr at 35ºC and the fungi were incubated for 7 to 13 days at 35ºC, as appropriate. Colonies harvested from these growth plates were resuspended in the appropriate medium containing a cryoprotectant. Aliquots of each suspension were then frozen at -80ºC.

Prior to testing, the yeast isolates were streaked from frozen vials onto Sabouraud Dextrose Agar (SAB; Becton Dickenson [BD]/BBL; Sparks, MD, Lot No. 2228660) and were incubated at 35°C for 24 hr. Additional fungal isolates were streaked from frozen vials onto potato dextrose agar (BD/BBL; Sparks, MD, Lot No. 2046481) and incubated under optimal conditions for growth.

All MIC measurements were performed in RPMI1640 from Hyclone Laboratories (Logan, UT; Cat. No. SH3001104; Lot No. AC10257966A) buffered with MOPS to pH-7 from Millipore (Billerica, MA; Cat No. 475898; Lot No. 3462216).

MIC values were determined using a broth microdilution procedure described by CLSI. [5,6]. Automated liquid handlers (Multidrop 384, Labsystems, Helsinki, Finland; Biomek 3000 and

Biomek FX, Beckman Coulter Fullerton CA) were used to conduct serial dilutions and liquid transfers.
Solutions of **1** were added in an 11 point 2x serial dilution in DMSO with a maximum tested concentration of 32 µg/ml in the culture medium. Drug solution was added at 1% v/v into 200 µl of culture in all wells.
A standardized inoculum of each test organism was prepared in appropriate media per CLSI methods.[5,6] The plates were then inoculated with 10 µL of the diluted inoculum using the Biomek 3000 from low to high drug concentration, resulting in a final concentration of approximately 0.5 to 2.5 x 10^3 CFU/mL for yeast and 0.2 to 2.5 x 10^4 CFU/mL for the other fungi. An un-inoculated plate was incubated for the purpose of assessing solubility of the drug in the test medium.
The plates were stacked 3 to 4 high, covered with a sterile lid on the top plate, and incubated aerobically at 35ºC for 24, 48, and 72 h. For yeast, both the MIC based on complete inhibition and 50% inhibition were reported at 24 and 48 h. For other fungal isolates, the MIC values were recorded at 24 and 48 hr and were based on complete inhibition and 50% inhibition.

Intact protein LCMS analysis

10 µM of recombinant *S. cerevisiae* ALS was incubated with 10 µM compound **1** in buffer (50 mM Tris pH 7.5, 100 mM KCl, 10 uM FAD, 1 mM TPP, 10 mM $MgCl_2$ (with 2.5% DMSO from compound)) at 22 ºC for 30 min. Unreacted **1** was removed by passing the reaction mixture through a 30 kDa MWCO filter (Millipore). The collected protein was then denatured and reduced by incubation with 6 M urea and 10 mM DTT at 37ºC for 1 h followed by 20 mM iodoacetamide alkylation for 30 min in dark. This reaction mixture then underwent buffer exchange into 100 mM Tris-HCl pH 8.0 and 10 mM $CaCl_2$ buffer using a 30 kDa MWCO filter. The protein was then incubated with sequencing grade chymotrypsin (Promega) (15:1,w/w) for 2 h at 37 ºC.
After digestion, the peptide mixture was immediately analysed by LC-MS/MS using a Waters I-class UPLC equipped with an ACQUITY UPLC BEH C18 column (2.1x50 mm, 1.7 µm, Waters) at 50ºC coupled to a Waters Synapt G2Si MS. The sample was injected and subjected to an LC gradient starting from 0.5% mobile phase B (ACN with 0.1% formic acid) for 5 min with linear increase to 40% B over 35 min. Mobile phase A was 0.1% formic acid water. Flow rate was maintained at 0.3 ml/min. MS/MS data was acquired in positive ionization mode with data dependent MS/MS acquisition. Peptide mapping was performed with PEAKs Studio 8.0 (Bioinformatics Solutions Inc.) followed by manual review to identify peptides putatively modified by covalent addition of **1**.

Protein expression and Purification for Cryo-EM

*Af*ALS(residues S112-K752) with an N-terminal His_6 tag followed by a TEV protease cleavage site was cloned in a pET28a vector and transformed into *E. coli* BL21 (DE3) cells for protein expression. These expression stains were grown at 37°C in Terrific Broth to mid-exponential phase (OD_{600} = 0.6) followed by induction with 0.4 mM isopropyl β-D-1-thiogalactopyranoside (IPTG) at 16°C for 18 hours.

Cells were harvested and resuspended in buffer (50 mM K_2HPO_4, pH 8.0, 300 mM NaCl, 10 μM FAD, 5 mM imidazole). The slurry was then lysed by sonication and clarified by centrifugation. The supernatant was then applied to a TALON column and washed with wash buffer (50 mM K_2HPO_4, pH 8.0, 300 mM NaCl, 10 μM FAD, 10% glycerol, 0.5 mM TCEP). *Af*ALS wesswas then eluted with elution buffer (500 mM K_2HPO_4, pH 8, 10 μM FAD, 0.5 mM TCEP, 200 mM imidazole, 10% glycerol). The eluate was further purified by size exclusion chromatography (Superdex 200 Increase 10/300 GL, Cytiva) with formulation buffer. Purity of the proteins was analyzed by SDS-PAGE after each step and the purified proteins were concentrated and stored at -80°C.

Cryo-EM sample preparation, data collection and processing

The *Af*ALS complex with **1** was prepared by mixing 1mg/mL *Af*ALS with 0.7 mM **1** followed by incubation on ice for 0.5 h. Both Apo and **1** bound-*Af*ALS were diluted to 0.5mg/mL with formulation buffer for cryoEM grid freezing. 3 μl of sample was applied to Ultra Au 300 1.2/1.3 (Quantifoil, Inc) pretreated by glow discharging. The grid was blotted at 4°C with 100% humidity and plunge-frozen in liquid ethane using FEI Vitrobot Mark IV (Thermo Fisher). The grids were stored in liquid nitrogen before screening and data collection.
Images were acquired on a Krios G4 Cryo-Transmission electron microscope (Thermo Fisher) equipped with Falcon 4i Direct Electron Detector in counting mode, at a calibrated magnification of 165,000X with the physical pixel size corresponding to 0.743 Å. Detailed data collection statistics have been indicated in **Table S2**. Motion correction of each micrograph, contrast transfer function (CTF) estimation, particle picking, 2D classification, ab initio 3D reconstruction, heterogeneous refinement, homogeneous refinement,, and local refinement were carried out by standard pipeline in cryoSPARC[7], 3D classification and 3D auto-refine were carried out in RELION.[8] For **1**-bound *Af*ALS, 3,065,801 initial particles were extracted from 6014 movies, and after iterative 2D classification 411,943 particles were selected for heterogeneous refinement, from which a class of 263,024 particles was further selected for homogeneous refinement. The particles were then imported to RELION for RELION SIRM reconstruction. The generated model, which still had preferred orientation, was then further processed by 3D classification, and a subset of 125,472 particles was selected for 3D auto-refine. The particles and volume were then imported back to cryoSPARC for final polishing. Homogeneous refinement and local refinement plus CTF refinement generated a final reconstruction with C2 symmetry at a resolution of 2.36 Å, based on the Fourier shell correlation (FSC) cutoff at 0.143 between the two half maps. The Apo *Af*ALS reconstruction was obtained through the similar image processing workflow, and a final reconstruction with C2 symmetry at a resolution of 2.76 Å was obtained from 64,577 particles. The final map was of excellent quality consistent with the resolution (**Figure S12**).

Model building and refinement

A homogenous *Af*ALS structure was obtained by SWISS model[9] using the *Sc*ALS crystal structure (PDB ID 5IMS[10]) as the template. Then the *Af*ALS model was docked into the cryo-EM map using Chimera.[11] Both Apo and **1**-bound *Af*ALS structures were further built and refined in COOT.[12] The structure of **1** was built by eLBOW in PHENIX and docked into the map for further

refinement. Both structures were further refined in PHENIX[13] with secondary structure restraints.[14] The final model with good geometry and fit to the map (**Figure S12B**) was validated using the comprehensive cryo-EM validation tool implemented in PHENIX[13] (**Table S2**). All structural figures were generated using PyMol[15] and ChimeraX.[16]

Construction of *A. fumigatus* AF293 *ilv2Δ* and the reconstituted strain

The *A. fumigatus ilv2Δ* (Afu3g10310) mutant was generated in the reference strain AF293[17] using CRISPR-Cas9 technology as described previously.[18] Briefly, two crRNAs adjacent to PAM motifs (g201: AATTGGCCTTTGGCCGCGCT and g202: AGTGATCATGTAGACGCTTT) that target Cas9 cleavage in the upstream and downstream of the ilv2 gene were designed. On the day of transformation, crRNA-tracrRNA duplex was generated by mixing equimolar concentrations of each crRNA with tracrRNA in duplex buffer, boiling for 5 minutes, and then allowing them to anneal by cooling to room temperature. This resulting duplex was combined with the Cas9 enzyme to generate the Cas9-RNP complex. We generated the repair construct by amplifying the hygromycin expression cassette with 35bp overhang primers (RAC 7458 and RAC 7459, **Table S6**) adjacent to the Cas9 targeted sites. Next, the Cas9-RNP complex and 2 μg of repair construct were transformed into AF293 protoplasts.[19] The transformants were selected on glucose minimal media (GMM)[20] with 1.2 M soribitol (SMM), hygromycin (175 μg/mL), and 5 mM each of isoleucine and valine.

Replacement of *ilv2* was confirmed using junction PCRs (RAC 7462/RAC6355, and RAC6562/RAC7463, **Table S6**) and by checking for the absence of the open reading frame (using RAC7460 and RAC7461, **Table S6**) in the mutant. The mutant's auxotrophy was confirmed by its ability to grow only on GMM supplemented with 5mM each of isoleucine and valine (**Figure S10**).

For the generation of the reconstituted strain, the *ilv2* locus (including 949bp of the promoter and 652bp of the terminator/3'UTR region) was amplified using primers RAC7498 and RAC7499 (**Table S6**), and the resulting product was integrated into a plasmid containing pyrathimaine (*ptrA*) selection marker using HiFi (NEB), such that the selection marker lies downstream of *ilv2*. The repair construct was amplified from the plasmid using RAC7502/RAC7503 (**Table S6**), which included a 35-bp overhang on each primer flanking the Cas9 cut site at the *aft4* safe haven site.[21] The sequence for the crRNA that targets the *aft4* site is g65 - TCTCCTTCATAAGCGACCAG. The Cas9-RNP complex and 2 μg of repair construct were transformed into Afu3g10310 protoplasts, and the transformants were selected on SMM containing 100 μg/L pyrithiamine hydrobromide. Integration of the gene at the *aft4* site was confirmed by junction PCRs (RAC6583/RAC7503, RAC6558/RAC6584, **Table S6**) and a PCR amplifying a region within the ORF of the gene (RAC7460 and RAC7461, **Table S6**).

To confirm the restoration of gene expression, RT-qPCR was performed on four candidates, using AF293 and Afu3g10310 as controls. Briefly, 10^5 conidia/ml of each strain were cultured in GMM containing 5 mM each of isoleucine and valine. After 16 hours, the adhered biofilms were scraped and resuspended in Trisure reagent. All samples were bead-beaten for 1 minute, and manufacturer instructions were followed thereafter. 500 ng of RNA was used for cDNA

synthesis. cDNA was then diluted 1:4 with water and 2 µL per reaction was used for qPCR. The gene expression was normalized to housekeeping genes (tubulin and actin), and fold change was calculated relative to wild-type.

Assessing the pathogenicity of *A. fumigatus* AF293 variants in a mouse model of invasive aspergillosis

All strains used in the animal experiment were grown on GMM supplemented with 5 mM each of isoleucine and valine for 3 days at 37°C, 5% CO_2. On the day of inoculation, the conidia were collected in 0.01% v/v Tween 80 in water, washed, counted, and then resuspended in PBS for inoculation.

Outbred 20-24 g female CD-1 mice (Charles River Laboratory, Raleigh, NC, USA) were housed in autoclaved cages (3-4 per cage) with HEPA-filtered air, food, and autoclaved water available *ad libitum*. For immunosuppression, the mice were subcutaneously administered 40 mg/kg Kenalog-10 (triamcinolone acetonide; Bristol-Myer Squibb, Princeton, NJ).[22] After 24 hrs, the mice were intranasally administered with 2×10^6 conidia of each strain in 40 µl sterile PBS or PBS alone and monitored for survival.

To determine the fungal burden in the lungs, the mice were sacrificed 72 hours after inoculation. The lungs were harvested, flash frozen, and the relative fungal burden was assessed through qPCR quantitation of *A. fumigatus* 18S rDNA (the gene for the 18S rRNA), as previously described.[23,24]

Ethics statement

The animal protocol was approved by the Institutional Animal Care and Use Committee (IACUC; federal-wide assurance number A3259-01) at Dartmouth College. The experiments were carried out with strict adherence to the recommendations in Guide for the Care and Use of Laboratory Animals.[25]

Spectroscopic Analyses

HPLC purifications were performed using either a Biotage Selekt Flash Chromatography, a Thermo Vanquish Flex HPLC or an Agilent 1260 HPLC. HPLC-HRMS analyses were acquired using a Thermo Vanquish HPLC system coupled to a Thermo Orbitrap Exploris 120 high-resolution mass spectrometer at 120 K resolution for MS1 and 17.5 K resolution for MS/MS. All chromatographic methods used 0.1% aqueous formic acid (A) and acetonitrile with 0.1% formic acid (B) as mobile phases unless described otherwise. For elucidation of chemical structures, 1D and 2D NMR spectra were obtained on Bruker AV600 spectrometer (^{1}H 600 MHz, ^{13}C 150 MHz) equipped with a BBO cryoprobe. Chemical shifts were referenced using the solvent peak or TMS (δ 0).

Isolation of compounds:

Isolation of compounds 1, 13, 14, 15, 16, 17 and 19

A. nidulans expressing *sdtABCDFGH* were cultured in 2 L flasks containing 1 L LMM media (12 L total). Each liter of culture was extracted by adding a sealed nylon bag containing 10 gm each of Amberlite XAD7HP and Diaion HP20 resin and shaken at 150 rpm for 16 h at room temperature. Resin bags from each flask were collected using long tweezers, combined and extracted with ethyl acetate for 3 h. The organic extract was filtered and dried. The crude extract was subjected to flash chromatography using a C18 column (120 g, pore size), with a flow rate of 50 mL/min, 5% solvent B was used for 1.5 column volumes (CV), a gradient of 5-30% solvent B for 3.5 CV, a gradient of 30-100% solvent b for 3 CV, and a wash of 100% solvent B for 2 CV. Target fractions containing metabolites of interest were identified by HPLC-HRMS using a linear gradient of 5-100% B in 10 minutes. Fractions containing compounds **1**, **13** and **19** were combined and further fractionated by semi-preparative HPLC (XBridge BEH Shield RP18 OBD, 10 × 250 mm, 10μm) and an isocratic method with 43%B for 60 mins, yielding **1** (0.5 mg/L), **13** (0.1 mg/L) and **19** (0.1 mg/L). Fractions containing **14** and **16** were combined and further fractionated by preparative HPLC using a C18 column (Eclipse XBD-C18 21.2 x 250 mm, 7 um), with a gradient of 50-100%B over 34 min (0-5 min, 50%B; 5-25 mins, 50-60% B; 25-27 mins, 60-100%B ; 27-34 min, 100%B) at 25 mL/min, yielding **14** (3.0 mg/L) and **16** (0.5 mg/L). Fractions containing **15** were combined and further fractionated using the same C18 column, with a gradient of 50-100%B over 27 min (0-5 min, 50%B; 5-20 mins, 50-62% B; 20-22 min, 62-100%B ; 22-27 min, 100%B) at 25 mL/min, yielding **15** (1.0 mg/L). Fractions containing **17** were combined and further fractionated also using the same C18 column with a gradient of 50%-100%B over 35 min (0-5 min, 50%B; 5-25 min, 50%-68%B; 25-28 min, 68%-100%B; 28-35 min, 100%B) at 25 mL/min, yielding 17 (0.1 mg/L)

Isolation of compound 10

Aspergillus nidulans expressing *sdtBDF* were cultured in 2 L flasks containing 1 L LMM media (12 L total). Each liter of culture was extracted by adding a sealed nylon bag containing 10 grams each of Amberlite XAD7HP and Diaion HP20 resin and shaken at 150 rpm for 16 h at room temperature. Resin bags from each flask were collected using long tweezers, combined and extracted with ethyl acetate for 3 hours. The organic extract was filtered and dried. The crude extract was subjected to flash chromatography using a C18 column (120 g, pore size) with a flow rate of 50mL/min, 5% solvent B was used for 1.5 column volumes (CV), a gradient of 5-30% solvent B for 3.5 CV, a gradient of 30-100% solvent b for 3 CV, and a wash of 100% solvent B for 2 CV. Target fractions containing metabolites of interest were identified by LC-HRMS using a linear gradient of 5-100% B in 10 minutes, Fractions were further fractionated by preparative HPLC using a C18 column (Eclipse XBD-C18 21.2 x 250 mm, 7 um),with a gradient of 70-100%B over 35 minutes (0-5 min, 70%B; 5-25 mins, 70-90% B; 25-27 mins, 90-100%B ; 27-35 min, 100%B) at 25mL.min^{-1}, yielding **10** (2 mg/L)

Isolation of compounds 11 and 12

For isolation of **11**, spores of the *A. nidulans* expressing *neoBDFH* were inoculated into 5 L CD-ST agar media and grown for 4 days at 28°C. The culture was exhaustively extracted with EtOAc, and the organic solvent was evaporated to dryness under vacuum. The resulting crude extract was fractionated by flash column chromatography subjected to an acetonitrile-water gradient (5:95 to 100:0). Fractions containing **11** were purified by semi-preparative HPLC using a C18 column (AR-II) and a gradient of 70-95% B over 30 min to yield **11** (0.4 mg/L).
For isolation of **12**, spores of the *A. nidulans* expressing *neoABDFH* were inoculated into 5 L CD-ST agar media and grown for 4 days at 28°C. The culture was exhaustively extracted with EtOAc, and the organic solvent was evaporated to dryness under vacuum. The resulting crude extract was fractionated by flash column chromatography subjected to an acetonitrile-water gradient (5:95 to 100:0). Fractions containing **12** were purified by semi-preparative HPLC using a C18 column (AR-II) and a gradient of 70-95% B over 30 min to yield **12** (0.4 mg/L).

Structure elucidation of isolated compounds

Purified HB-35018 (**1)** was isolated as a off-white solid. HRMS m/z 386.2328 for the $[M+H]^+$ ion was consistent with a molecular formula $C_{23}H_{31}NO_4$. NMR data was acquired for **1** in CD_3OD (**Table S7**, **Figures S13-S18**). ^1H, ^{13}C and ^1H-^{13}C HSQC showed the presence of 23 carbons and 29 observable protons. Of the 23 carbons, seven are sp^2 hybridized, among them 2 carbonyls at δ 209.0 and δ 211.0, one carboxyl at δ 173.5 and four olefinic carbons at δ 126.1, δ 130.6, δ 130.7 and δ 137.5. The remaining 16 (sp^3) carbons were identified as one aminomethine, one oxymethylene, four methines, five methylenes, three methyls and two quaternary carbons. COSY cross-peaks allowed the determination of spin systems for H-2-H-3, H-5-H-9, H-11-H_3-14 and H-4’-H_3-7’. A weak COSY correlation between H-3-and H-8 allowed the establishment of the bicyclic decalin scaffold and suggested a *cis*-decalin configuration based on J coupling constants. HMBC correlations from H_3-15 to C-2’, C-2, C-3 and C-4, and from H_3-16 to C-9, C-10 and C-11 allowed the exact placement of methyl groups. The HMBC correlation from H-11 to C-1, from H_3-15 to C-2’ and from H-2 to both C-1 and C-2’ provided the basis for establishing a rare hexahydro-acenaphthene-1-one tricyclic core. HMBC correlations from H_2-5’ to C-3’ and C-4’, and from H-4’ to C-1’ and C-3’ indicated an alkyl tetramic acid scaffold. While there is no direct NMR evidence, the only possible connection between these two ring systems would be through a carbocyclic spiro formation at the quaternary carbon C-2’. The observation of NOESY correlation among H-3, H-8 and H_3-15, as well as between H-2 with both Ha-12 and Ha-13 allowed the assignment of relative configurations for C-2, C-3, C-4, C-8 and C-11. The absolute configuration was determined by X-ray analysis, confirmed as 2R, 3R, 4R, 8R, 11R, 2’R, 4’R.
Compounds **13** and **19** were also obtained as off-white solids. HRMS indicated the same molecular formula of **1**, $C_{23}H_{31}NO_4$. NMR data indicated a scaffold identical to **1** for both compounds. The main differences observed were NOE correlations from H-4’ to H-5 and H_3-15 in both **13** and **19**. This spatial proximity could be attributed to a direct stereochemical change at the C-4’carbon, or by an inversion of the stereocenter at the spirocyclic carbon C-2'. Careful analysis of the ^1H NMR shifts in **13** and **19** revealed that while there is minimal impact to other proton chemical shifts in **19**, there is a noticeable upfield shift for H-5 and downfield shift for

H_3-15 for compound **13**. The absolute configuration of **1** indicates that the keto carbonyl at C-3’ faces the same side as H_3-15, while the amide carbonyl at C-1’stacks with H-5. An inversion in the spirocyclic carbon stereocenter changes the overlap between the carbonyl carbons C-1’and C-3 and the protons at H-5 and H_3-15. As such, we have designated compound **13** as the 2'S isomer of **1**, and **19** as the 4'S isomer of **1**.

Unfortunately, despite extensive effort, the 2'S, 4'S isomer of **1** was not obtained in sufficient amount for NMR characterization. However, a set of four intermediates (**14-17**) were isolated, representing all four possible stereoisomers. They were all isolated as off-white solids and showed a HRMS m/z of 400.2118 as the $[M+H]^+$ ion, indicating a molecular formula of $C_{23}H_{29}NO_5$. As expected, strong agreement in 1D chemical shifts and 2D correlations for all compounds were observed, similar to **1** and its isomers, with the major difference identified as the presence of a carboxylic acid at C-7’. Analysis of the chemical shifts of H-5, H_3-15 and H-4’and the presence or absence of NOE among them allowed the identification of **14** as the C-7’COOH analog of **19 (**2'S, 4'R), **16** as the C-7’COOH analog of **1 (**2'S, 4'S), **17** as the C-7’COOH analog of **13 (**2'R, 4'S)**,** and **15** as the 2'R, 4'R isomer.

Supporting Tables

Table S1: Allele frequencies of all mutations observed in *S. cerevisiae* ALS from clones raised to be resistant to **1**.

Mutation (S.cerevisiae)	Number of clones expressing this mutation	Frequency	Residue number in *Af*ILV2
A117V	1	4.3%	A176
V191L	1	4.3%	V250
P192S	3	13.0%	P251
P192L	15	65.2%	P251
A200V	1	4.3%	S259
W586R	1	4.3%	W648
S596F	1	4.3%	S658

Table S2: Cryo-EM data collection, refinement and validation statistics

Data collection and processing	apo AfALS	*AfALS + 1*
Magnification	165,000	165,000
Voltage (kV)	300	300
Electron exposure (e–/Å2)	65	65
Defocus range (μm)	-1 - -2.4	-1 - -2.4
Pixel size (Å)	0.743	0.743
Symmetry imposed	C2	C2
Initial particle images (no.)	3,284,254	3,065,801
Final particle images (no.)	64,577	125,472
Map resolution (Å)<br>FSC threshold	2.76<br>0.143	2.36<br>0.143
Map resolution range (Å)	2.5 – 3.5	2.0-2.7
**Refinement**		
Initial model used (PDB code)	5IMS	5IMS
Model resolution (Å)<br>FSC threshold	2.74<br>0.143	2.55<br>0.143
Model resolution range (Å)	2.74	2.55
Model composition<br>Nonhydrogen atoms<br>Protein residues<br>Ligands	 9122<br>1164<br>4	 9092<br>1154<br>6
B factors (Å2)<br>*Protein*<br>*Ligand*	 56.68<br>52.63	 83.69<br>90.87
R.m.s. deviations<br>Bond lengths (Å)<br>Bond angles (°)	 0.009<br>1.079	 0.002<br>0.635
Validation<br>MolProbity score<br>Clashscore<br>Poor rotamers (%)	 2.54<br>20.06<br>4.18	 1.75<br>7.12<br>1.48
Ramachandran plot<br>Favored (%)<br>Allowed (%)<br>Disallowed (%)	 96.02<br>3.98<br>0.00	 96.51<br>3.49<br>0.00

Table S3: Physical chemical and pharmacokinetic parameters for **1**

**Calculated properties**	MW | ClogP | tPSA (Å^2)	386 | 2.7 | 83
**Solubility**	PBS kinetic solubility, 5% DMSO (μM)	260
**Buffer stability**	PBS pH 7.4 $t_{1/2}$ (min)	141
**Plasma stability**	Plasma $t_{1/2}$ (min) Mouse | Human	88 | 79
**Liver microsome stability**	Liver microsome $t_{1/2}$ (min) Mouse | Human	3 | 3

Table S4: MIC values for **1** against a panel of pathogenic molds. Values for both >50% and 100% growth inhibition measured at both 24 and 48 hours are provided.

**Isolate**	**MIC (μg/mL)**			
	**24 hr**		**48 hr**	
	**>50%**	**100%**	**>50%**	**100%**
Aspergillus fumigatus ATCC MYA-3626	4	4	>32	>32
Aspergillus fumigatus CDC 0731	2	16	>32	>32
Aspergillus fumigatus CDC 0732	2	8	>32	>32
Aspergillus fumigatus CDC 0733	4	8	>32	>32
Aspergillus fumigatus CDC 0734	4	8	>32	>32
Aspergillus fumigatus CDC 0735	4	8	32	>32
Aspergillus fumigatus CDC 0736	4	8	16	>32
Aspergillus fumigatus CDC 0737	2	16	16	>32
Aspergillus fumigatus CDC 0738	1	2	32	>32
Aspergillus fumigatus CDC 0739	1	4	16	32
Aspergillus fumigatus CDC 0740	1	4	16	32
Aspergillus flavus ATCC 204304	>32	>32	>32	>32
Aspergillus niger ATCC 9508	1	2	8	32
Aspergillus terreus ATCC MYA-3628	>32	>32	>32	>32
Fusarium oxysporum ATCC 48112	>32	>32	>32	>32
Fusarium solani ATCC MYA-3636	>32	>32	>32	>32
Rhizopus oryzae ATCC 11886	0.5	1	8	16
Mucor circinelloides ATCC 24905	0.12	0.5	4	16
Scedosporium apiospermum ATCC MYA-3635	1	8	>32	>32
Scedosporium prolificans ATCC 11274	2	8	>32	>32

Table S5: Identity of residues shown to cause resistance across fungal species. Column titles are representative of the wild-type *S. cerevisiae* ALS.

Species	MIC	Ala_{117}	Val_{191}	Pro_{192}	Ala_{200}	Trp_{586}	Ser_{596}
S. cerevisiae resistance mutations	ND	Val	Leu	Ser, Leu	Val	Arg	Phe
A. fumigatus	2-16	Ala	Val	**Pro**	Ser	Trp	Ser
Aspergillus flavus	>32	Thr	Val	Ala	Ala	Trp	Ser
Aspergillus niger	2	Ala	Val	**Pro**	Ser	Trp	Ser
Aspergillus terreus	>32	Ala	Val	Ala	Ala	Trp	Ala
Fusarium oxysporum	>32	Ala	Val	Val	Ala	Trp	Ala
Fusarium solani	>32	Ala	Val	Val	Ala	Trp	Ala
Rhizopus oryzae	1	Ala	Val	**Pro**	Ala	Trp	Ser
Mucor circinelloides	0.5	Ala	Val	**Pro**	Ala	Trp	Ser
Scedosporium apiospermum	8	Ala	Val	Val	Ala	Trp	Ala
Scedosporium prolificans	8	Ala	Val	Val	Ala	Trp	Ala
A. Nidulans	ND	Ala	Val	Val	Ser	Trp	Ser
SdtC	ND	Ala	Val	Ala	Ala	Trp	Ala

Table S6: Primers used in the construction and validation of *A. fumigatus ilv2Δ* (Afu3g10310)

Primer name	Binds to	Sequence
RAC7458	Repair forward	ACATCGGAGTTCGCTGTCCAATAATGGCGTTAAGGTAC CGGTCGCCTCAAACAATGCTCT
RAC7459	Repair reverse	GAAAGATGAAAGATTAGTAAGCAACGTTGCAAAGCCGG TCTGAGAGGAGGCACTGATGCG
RAC7460	ORF forward	GAGTCATCCCTCGCTTACCA
RAC7461	ORF reverse	GCAATCTCGAAAGCCTCCTG
RAC7462	5' utr forward	AGGTCCATACTTGTGCTCTGT
RAC7463	3' utr reverse	AATGTCCGACAGAACCAGGG
RAC6355	Hygromycin reverse	cagaaagaacgcatccatca
RAC 6562	Hygromycin forward	gactgaggaatccgctcttg
RAC7498	ilv2p forward	AAGAACCGGTGAATTTGGCT
RAC7499	Ilv2t reverse	CCGCGGCTTTCAAGGATTT
RAC7502	Recon repair fwd	TCAGGTGCATCTTCCAGTTCTGGATATAGCATATAGATCT ATCTACCCCGCTTCTGATGC
RAC6321	Recon repair rev	TTTGGCCTCCATACTCCCCTGATCTCAATCCAGTTGAGA Acggccagtgccaagctctag
RAC6583	*aft4 site fwd*	GCCTCGATAACTGCCTTCAC
RAC7503	*ilv2 rev*	CTTTCTGTGTGCGGCCTATC
RAC6584	*aft4 site rev*	TCAGGCCGGGTTATAAGAGA
RAC6558	*ptrA fwd*	gcatgaaccggatttgtctt
RAC007	tubulin fwd	ATAATGTTCAGACCGCCCTCTGCT
RAC008	tubulin rev	GACGGATGTGGAATTGCCCACAAA
RAC158	actin fwd	TCACTGCCCTTGCTCCCTCGTC
RAC159	actin rev	GCACTTGCGGTGAACGATCGAA

Table S7: NMR spectroscopic data for **1**

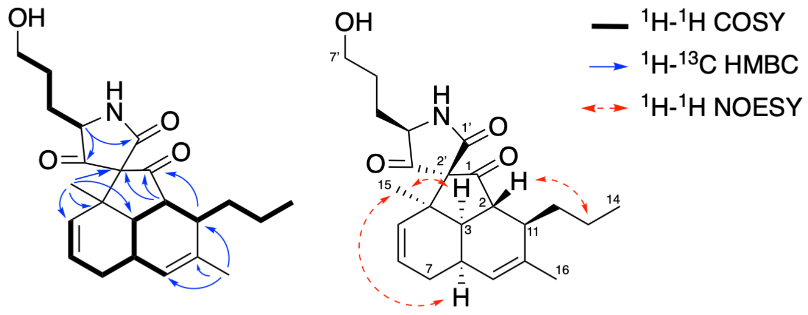

	Found (MeOD)	
Position	δ_C	δ_H, m(J in Hz)1
1	210.2	-
2	49.6	2.59, dd(14.1,10.4)
3	46.0	2.30. ddd(14.2, 4.3, 1.7)
4	47.1	-
5	131.1	5.80, ddt (10.4, 3.1, 1.6)
6	125.0	5.63, ddd(10.4, 5.3, 2.4)
7	29.0	Ha: 2.36, dddd (18.8, 7.0, 5.3, 1.7)/ Hb: 1.73, ddt(18.9, 10.1, 2.7)/
8	29.6	2.50, m
9	130.8	5.75, d(2.8)
10	137.4	-
11	42.7	2.43, m
12	32.0	Ha: 1.93, m/ Hb: 1.59, m
13	17.8	Ha: 1.24, m/ Hb: 1.03, m
14	14.9	0.89, t(7.2)
15	24.7	1.18, s
16	20.8	1.66, s
1’	172.1	-
2’	76.2	-
3’	209.9	-
4’	64.0	3.92, dd(8.3,5.2)
5’	30.1	1.60, m/1.81, m
6’	29.7	1.65, m
7’	62.2	3.58, t(6.1)

In MeOD, 600 MHz for ^{1}H and 150 MHz for ^{13}C NMR; Chemical shifts are reported in ppm. All shifts are determined by ^{1}H, ^{13}C, COSY, HSQC, HMBC and NOESY correlations.

Table S8: NMR spectroscopic data for **10**

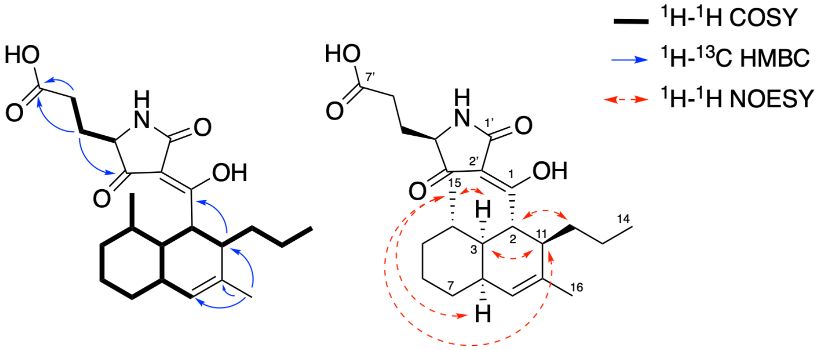

Position	δC	δH, m(J in Hz)
1	195.1	-
2	47.9	3.8, m
3	52.5	1.18 ,m
4	40.0	1.38 ,m
5	38.0	Ha: 1.69, m/ Hb: 1.02, m
6	27.2	Ha: 1.73, m/ Hb: 1.41, m
7	34.0	Ha: 1.93, m/ Hb: 1.22, m
8	41.6	1.75, m
9	131.1	5.41, s
10	138.3	-
11	43.0	2.34, m
12	32.6	Ha: 1.46, m/ Hb: 1.35, m
13	23.1	1.35, m
14	14.7	0.86, m
15	21.1	0.84, m
16	20.0	1.71, m
1'	177.1	-
2'	102.9	-
3'	197.3	-
4'	62.2	3.91, m
5'	28.1	Ha: 2.07, m/ Hb: 1.88, m
6'	29.8	2.34, m
7'	176.2	-

In MeOD, 600 MHz for ^1H and 150 MHz for ^{13}C NMR; Chemical shifts are reported in ppm. All shifts are determined by ^1H, ^{13}C, COSY, HSQC, HMBC and NOESY correlations.

Table S9: NMR spectroscopic data **for 11**

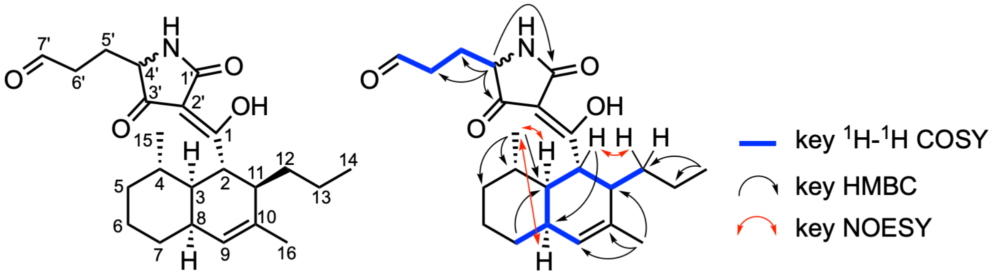

11

**Position**	**δC**	**δH, m(J in Hz)**
1	192.8	-
2	40.7	4.07 m
3	43.0	1.86 m
4	31.2	1.66 m
5	27.6	1.51 m
6	20.7	1.40 m
7	30.6	1.15 m
8	32.6	2.25 m
9	129.8	5.53 m
10	134.7	-
11	43.3	2.52 m
12	32.6	Ha: 1.51, m/ Hb: 1.35, m
13	18.0	Ha: 1.43, m/ Hb: 1.03, m
14	14.8	0.80 m
15	19.5	1.00 m
16	21.2	1.63 s
1'	176.3	-
2'	104.8	-
3'	196.3	-
4'	61.7	3.88 m
5'	24.6	Ha: 2.06, m/ Hb: 1.90, m
6'	39.1	Ha: 2.49, m/ Hb: 2.44, m
7'	202.6	9.67 m

In $CDCN_3$, 500 MHz for ^1H and 125 MHz for ^{13}C NMR; Chemical shifts are reported in ppm. All shifts are determined by ^1H, ^{13}C, COSY, HSQC, HMBC and NOESY correlations.

Table S10: NMR spectroscopic data for **12**

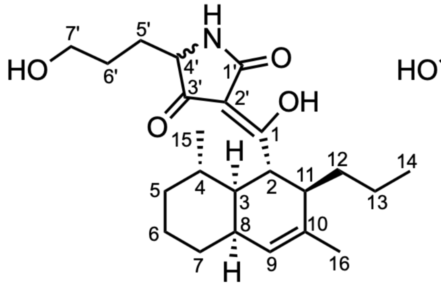

12

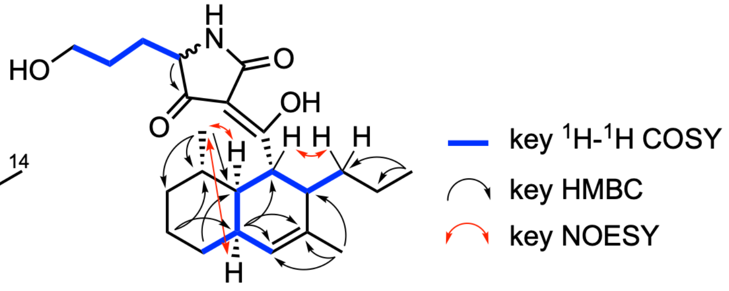

Position	δC	δH, m(J in Hz)
1	192.5	-
2	40.3	4.08 t (10.7)
3	42.5	1.89 m
4	30.8	1.64[a]
5	27.4	1.54[a]
6	20.4	1.42 m
7	28.5	1.18 m
8	32.4	2.24 m
9	129.3	5.52 d (5.8)
10	134.1	-
11	42.9	2.54 m
12	32.2	Ha: 1.49, m/ Hb: 1.21, m
13	17.7	Ha: 1.43, m/ Hb: 1.05, m
14	14.8	0.81 t (7.1)
15	19.4	1.00 d (7.2)
16	21.2	1.63 s
1'	175.4	-
2'	104.1	-
3'	195.5	-
4'	62.4	3.87 m
5'	30.2	Ha: 1.95, m/ Hb: 1.69, m
6'	29.4	Ha: 1.68, m/ Hb: 1.58, m
7'	62.7	3.66 m

In CD_2Cl_2, 500 MHz for ^{1}H and 125 MHz for ^{13}C NMR; Chemical shifts are reported in ppm. All shifts are determined by ^{1}H, ^{13}C, COSY, HSQC, HMBC and NOESY correlations.

Table S11: NMR spectroscopic data for **13**

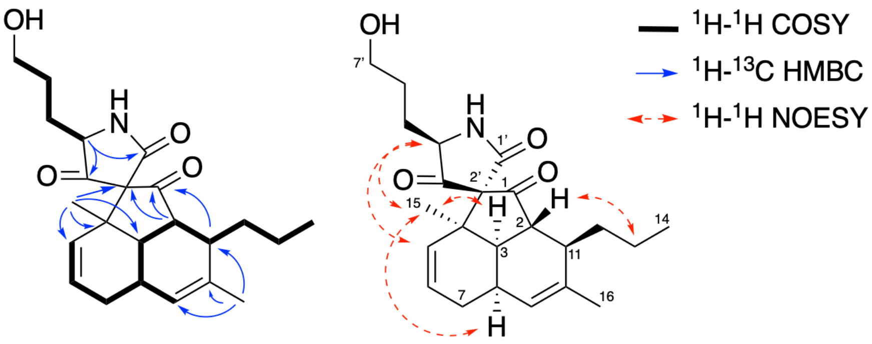

	Found (MeOD)	
Position	δ_C	δ_H, m(J in Hz)
1	211.0	-
2	49.7	2.46, m
3	46.0	2.34, m
4	48.0	-
5	130.7	5.4 d(10.5)
6	126.1	5.67 ddd(10.4, 5.3, 2.4)
7	29.1	Ha: 2.37, m/ Hb: 1.74, m
8	29.9	2.49, m
9	130.6	5.74, d (6.3)
10	137.5	-
11	42.3	2.43, m
12	32.0	Ha: 1.93, m/ Hb: 1.6, m
13	17.9	Ha: 1.20, m/ Hb: 1.03, m
14	14.9	0.89 t(7.2)
15	24.2	1.30, s
16	20.8	1.65, s
1’	173.5	-
2’	76.2	-
3’	209.0	-
4’	64.8	4.03, t (5.8)
5’	28.8	Ha: 1.88, m/ Hb: 1.72, m
06’	29.0	Ha: 1.64, m/ Hb: 1.60, m
7’	62.5	3.57, t (6.0)

In MeOD, 600 MHz for ^{1}H and 150 MHz for ^{13}C NMR; Chemical shifts are reported in ppm. All shifts are determined by ^{1}H, ^{13}C, COSY, HSQC, HMBC and NOESY correlations.

Table S12: NMR spectroscopic data for **14**

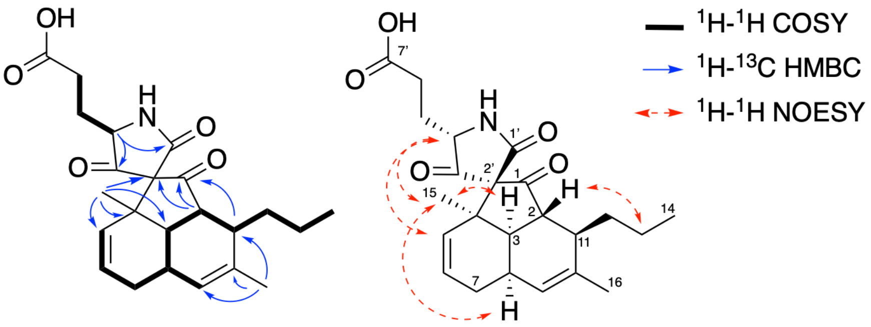

	Found (MeOD)	
Position	δ_C	δ_H, m(J in Hz)
1	211.1	-
2	49.5	2.53. m
3	46.0	2.26, dd(13.9, 1.5)
4	47.9	-
5	130.6	5.70, d(10.6)
6	125.5	5.65, ddd(10.4, 5.2, 2.2)
7	29.0	Ha: 2.34, t(6.01)/Hb: 1.73, ddt(19.0, 10.1, 2.6)
8	29.7	2.49, m
9	130.7	5.75, d(6.3)
10	137.5	-
11	42.4	2.44, m
12	32.0	Ha: 1.94, m/Hb: 1.61, ddt(14.1, 12.5, 4.2)
13	17.8	Ha: 1.24, m/Hb: 1.03, m
14	14.9	0.89, t(7.3)
15	24.2	1.19, s
16	20.8	1.66, s
1'	172.0	-
2'	76.0	-
3'	210.6	-
4'	63.8	4.05, t(5.9)
5'	27.8	Ha: 2.07, m/Hb: 1.94, m
6'	30.3	2.43, m
7'	176.38	-

In MeOD, 600 MHz for ^{1}H and 150 MHz for ^{13}C NMR; Chemical shifts are reported in ppm. All shifts are determined by ^{1}H, ^{13}C, COSY, HSQC, HMBC and NOESY correlations.

Table S13: NMR spectroscopic data for **15**

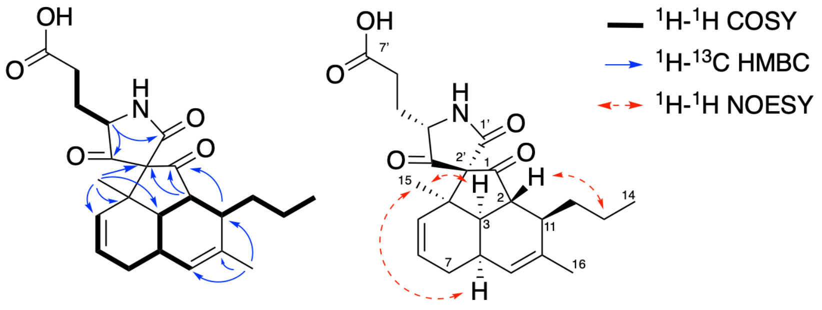

	Found (MeOD)	
**Position**	**δC**	**δH, m(J in Hz)**
1	210.6	-
2	49.7	2.44, m
3	46	2.37, m
4	47.6	-
5	130.6	5.44, m
6	125.7	5.68, m
7	28.9	Ha: 2.36, m; Hb: 1.73, ddt (18.5, 10.0, 2.1)
8	29.7	2.51, m
9	130.6	5.75, d(4.6)
10	137.4	-
11	42.6	2.45, m
12	32.1	Ha:1.92, m/Hb: 1.60, m
13	17.9	Ha: 1.20, m/Hb: 1.04, m
14	14.9	0.89, t(7.1)
15	24.4	1.34, br s
16	20.8	1.66, s
1’	173.3	-
2’	76.1	-
3’	208.4	-
4’	63.6	3.97, t(7.4)
5’	28.4	Ha: 1.98, m/Hb: 1.84, m
6’	31.0	2.46, m
7’	176.5	-

In MeOD, 600 MHz for ^1H and 150 MHz for ^{13}C NMR; Chemical shifts are reported in ppm. All shifts are determined by ^1H, ^{13}C, COSY, HSQC, HMBC and NOESY correlations.

Table S14: NMR spectroscopic data for **16**

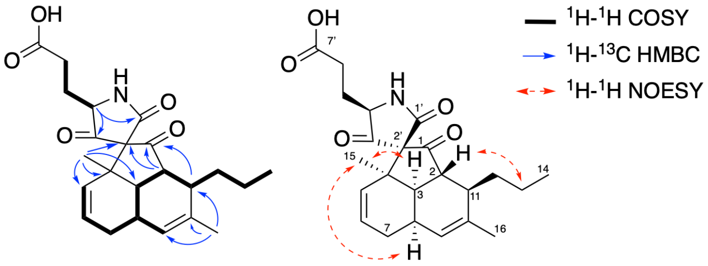

	Found (MeOD)	
Position	δ_C	δ_H, m(J in Hz)
1	210.1	-
2	49.6	2.59, m
3	46.0	2.28, m
4	47.2	-
5	131.0	5.80, d(10.5)
6	125.0	5.65, m
7	28.9	Ha: 2.36, m/Hb: 1.73 dd(18.9, 10)
8	29.6	2.5, m
9	130.7	5.75, d(5.93)
10	137.4	-
11	42.7	2.43, m
12	32.0	Ha: 1.93, m/Hb: 1.61, m
13	17.8	Ha: 1.23, dp(12.6, 5.9) /Hb: 1.03, m
14	14.9	0.89, t(7.1)
15	24.7	1.19, s
16	20.8	1.66, s
1’	172.0	-
2’	76.2	-
3’	209.6	-
4’	63.2	3.97, t(5.9)
5’	28.7	Ha: 2.03, m/Hb: 1.81, dt(14.0, 7.3)
6’	31.0	2.46, m
7’	176.3	-

In MeOD, 600 MHz for ^1H and 150 MHz for ^{13}C NMR; Chemical shifts are reported in ppm. All shifts are determined by ^1H, ^{13}C, COSY, HSQC, HMBC and NOESY correlations.

Table S15: NMR spectroscopic data for **17**

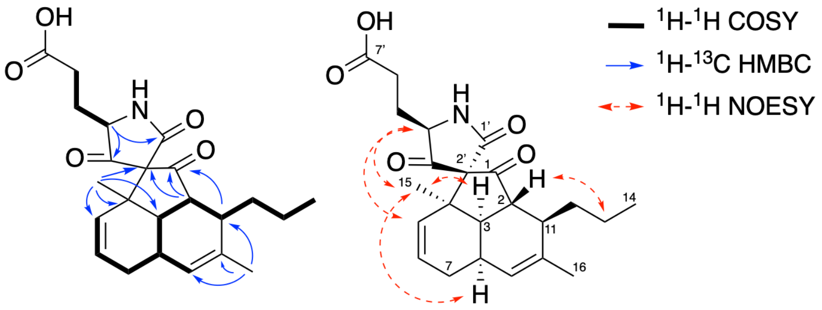

	Found (MeOD)	
**Position**	**δC**	**δH, m(J in Hz)**
1	211.1	-
2	49.7	2.47, m
3	45.9	2.34, m
4	48	-
5	130.5	5.44 d(10.5)
6	126	5.68 ddd(14.9, 10.4, 3.9)
7	29	Ha: 2.35, m/ Hb: 1.76, m
8	30.3	2.46, m
9	130.7	5.75, d (5.6)
10	137.5	-
11	42.3	2.46, m
12	32	Ha: 1.92, m/ Hb: 1.62, m
13	17.9	Ha: 1.22, m/ Hb: 1.05, m
14	14.9	0.90 t(7.2)
15	24.1	1.34, s
16	20.9	1.67, s
1’	173.4	-
2’	76.2	-
3’	208.9	-
4’	63.9	4.08, t (5.9)
5’	27.4	Ha: 2.07, m/ Hb: 1.95, m
6’	30.2	2.46
7’	176.4	-

In MeOD, 600 MHz for ^{1}H and 150 MHz for ^{13}C NMR; Chemical shifts are reported in ppm. All shifts are determined by ^{1}H, ^{13}C, COSY, HSQC, HMBC and NOESY correlations.

Table S16: NMR spectroscopic data for **19**

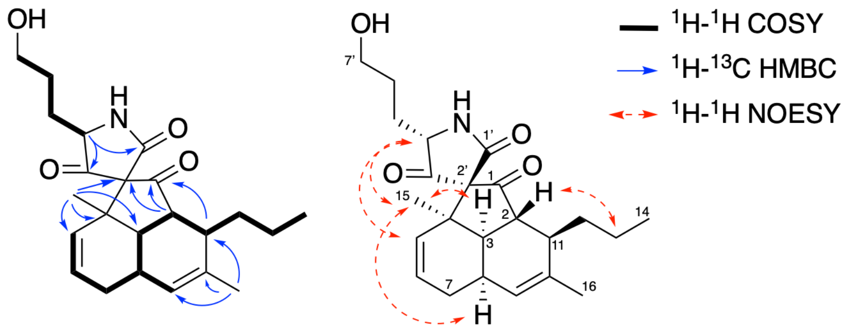

	Found (MeOD)	
Position	δ_C	δ_H, m(J in Hz)
1	210.8	-
2	49.4	2.51, m
3	45.9	2.26, dd(14.1, 3.5)
4	47.9	-
5	130.8	5.72, d(10.7)
6	125.5	5.65, m
7	29.1	Ha: 2.36, m/ Hb: 1.74, m
8	29.8	2.49, m
9	130.7	5.75, d(5.1)
10	137.5	-
11	42.5	2.44, m
12	32.0	Ha: 1.94, m/ Hb: 1.59, m
13	17.8	Ha: 1.24, m/ Hb:1.00, d(8.3)
14	14.9	0.89, t(7.1)
15	24.13	1.20, s
16	20.7	1.66, s
1’	172.9	-
2’	76.1	-
3’	211.0	-
4’	64.6	4.00, t(5.9)
5’	29.2	Ha: 1.87, m/ Hb: 1.72, m
6’	29.0	Ha: 1.65, m/ Hb: 1.59, m
7’	62.4	3.57, t(6.1)

In MeOD, 600 MHz for ^{1}H and 150 MHz for ^{13}C NMR; Chemical shifts are reported in ppm. All shifts are determined by ^{1}H, ^{13}C, COSY, HSQC, HMBC and NOESY correlations.

Supporting Figures

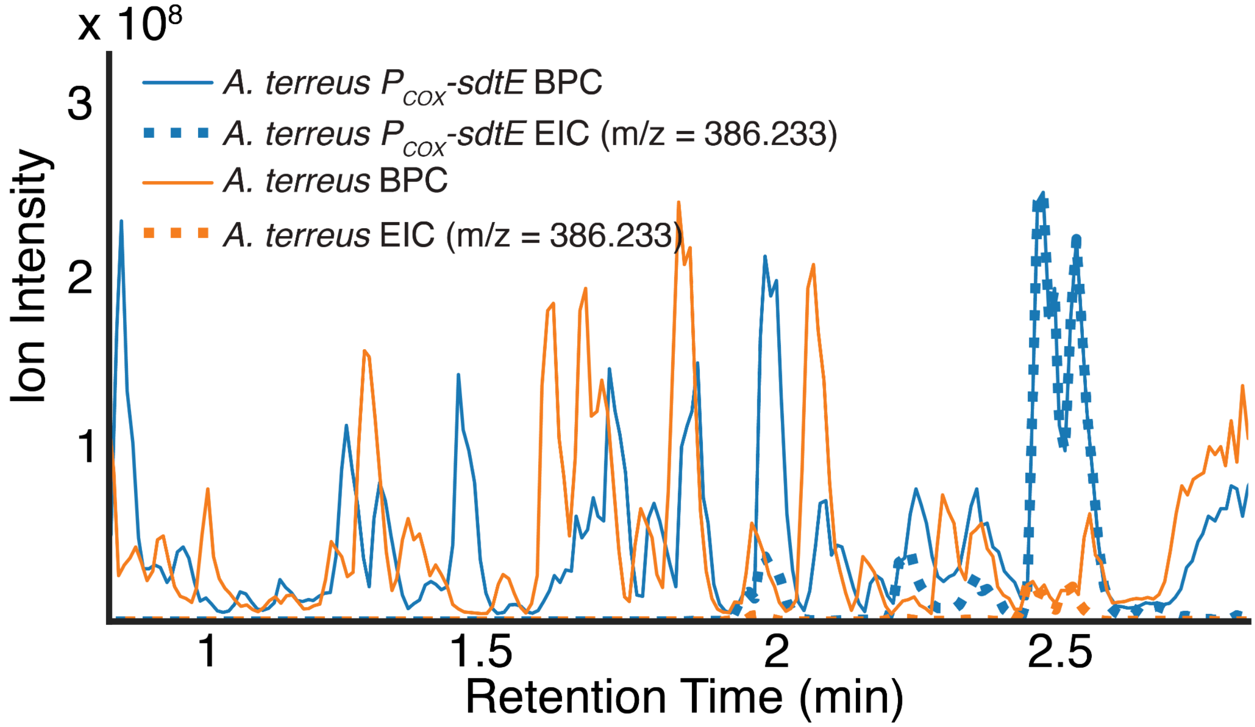

Figure S1: Base peak chromatograms (BPCs) and extracted ion chromatograms for wild-type *A. terreus* and *A. terreus* P_{cox}-*sdtE*. A representative trace chosen from 4 biological replicates of each strain is shown.

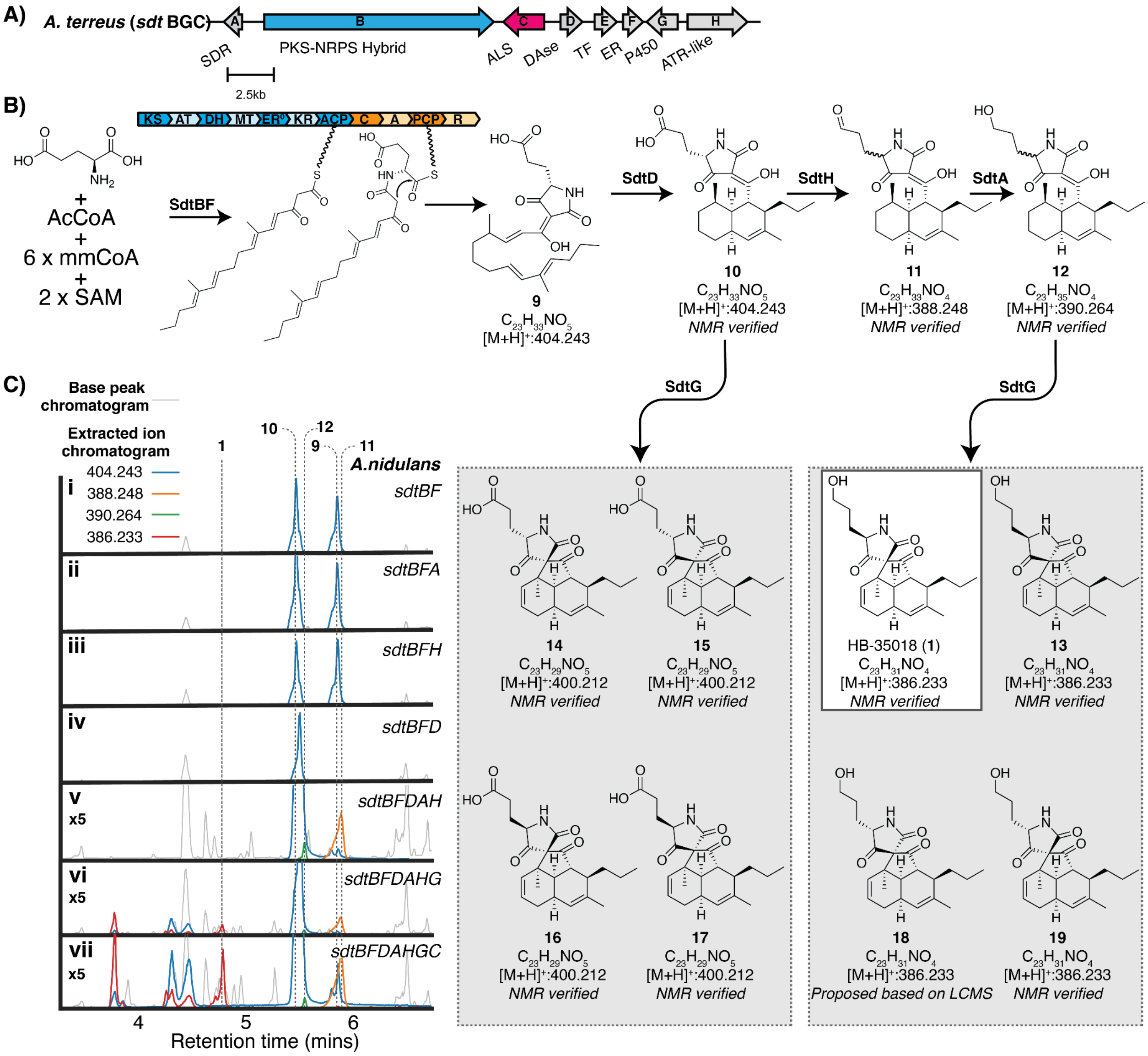

Figure S2: Detailed proposed biosynthesis of **1** inclusive of all isolated shunts and intermediates. Abbreviations: short chain reductase reductase (SDR), polyketide synthase/non-ribosomal synthetase hybrid (PKS/NRPS hybrid), Diels-Alderase (DAse), transcription factor (TF), enoyl-reductase (ER), cytochrome P450 (P450), adenylation-thiolation-reductase-like (ATR-like), ketosynthase (KS), acyltransferase (AT), dehydratase (DH), methyltransferase (MT), ketoreductase (KR), acyl carrier protein (ACP), condensation (C), adenylation (A), peptidyl carrier protein (PCP), reductase (R).

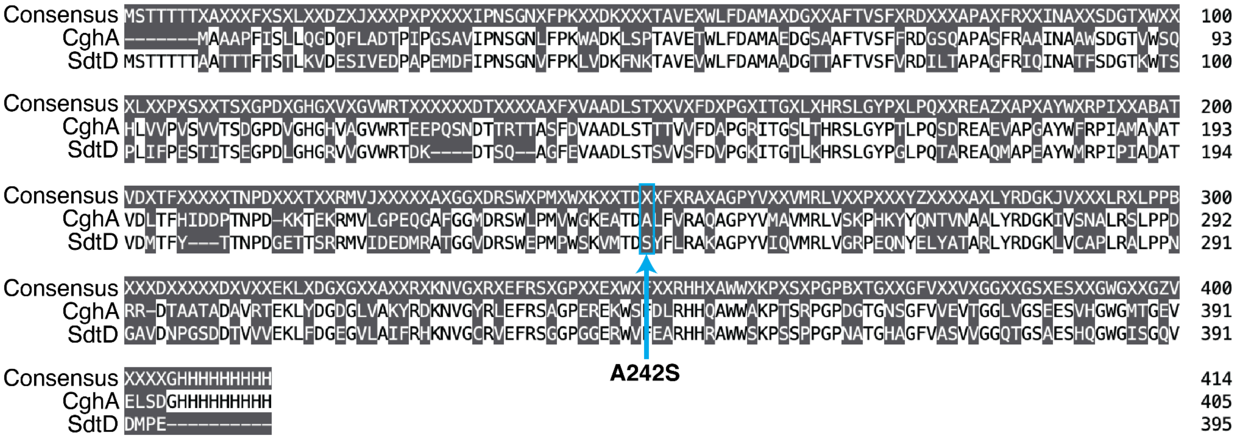

Figure S3: Sequence alignment of CghA and SdtD highlighting the A to S difference at position 242.

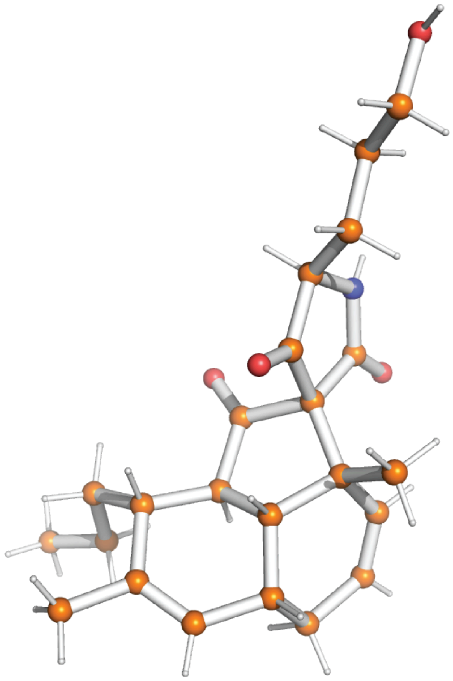

Crystal system	orthorhombic
Space group	$P2_12_12_1$
a [Å]	7.6508 ± 0.0005
b [Å]	9.8384 ± 0.0006
c [Å]	28.002 ± 0.002
α [º]	90
β [º]	90
γ [º]	90

Figure S4: Small molecule X-ray structure of **1.**

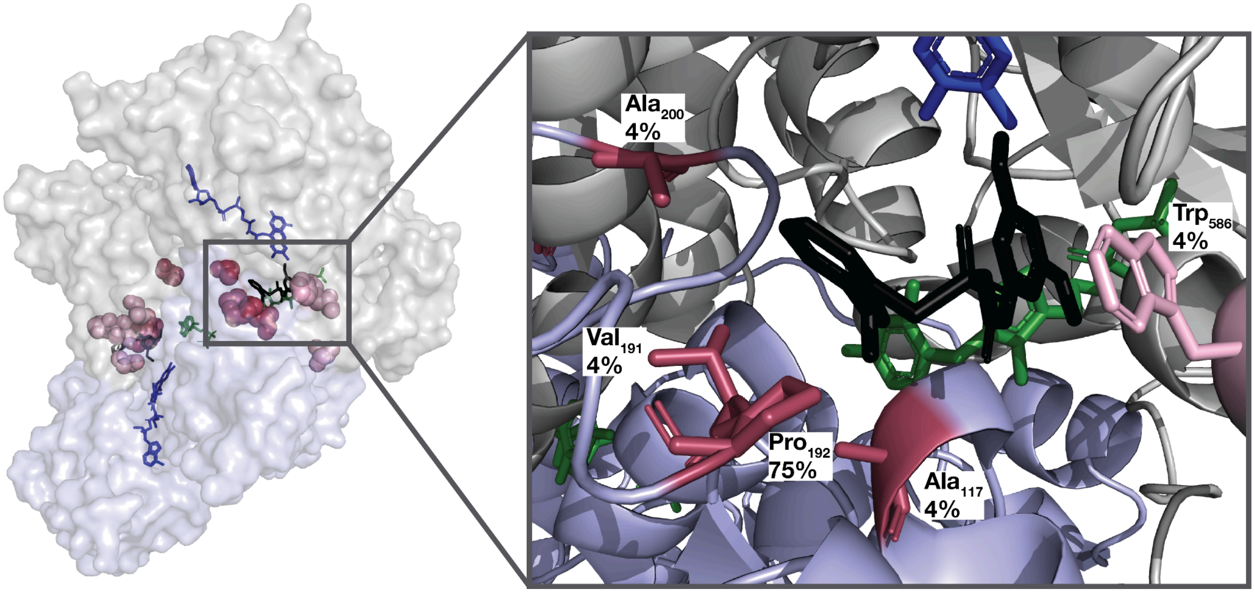

Figure S5: Mutations in ALS observed in *S. cerevisiae* clones resistant to **1**. Resistance mutations (shown in pink and raspberry) are mapped onto the crystal structure of **2** (black) bound to S. cerevisiae ALS (PDB:5FEM30, α-chain in gray, β-chain in blue). Essential thiamine pyrophosphate (TPP,green) and FAD (blue) cofactors shown. All mutations are annotated with the percentage of clones in which the residue was mutated.

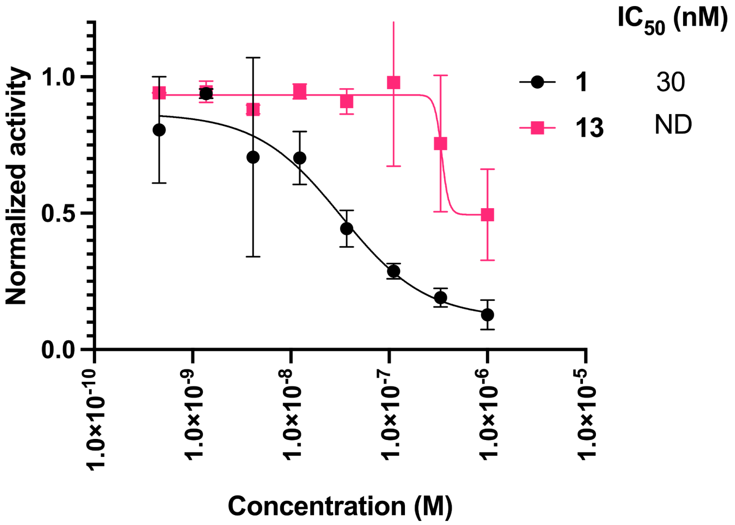

Figure S6: Inhibition of recombinant *S. cerevisiae* ALS by **1** and its C12 diastereomer **13**

A)

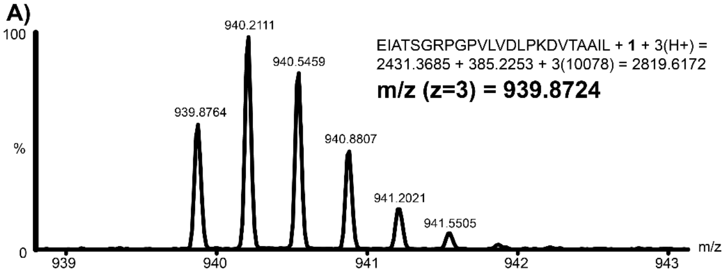

B)

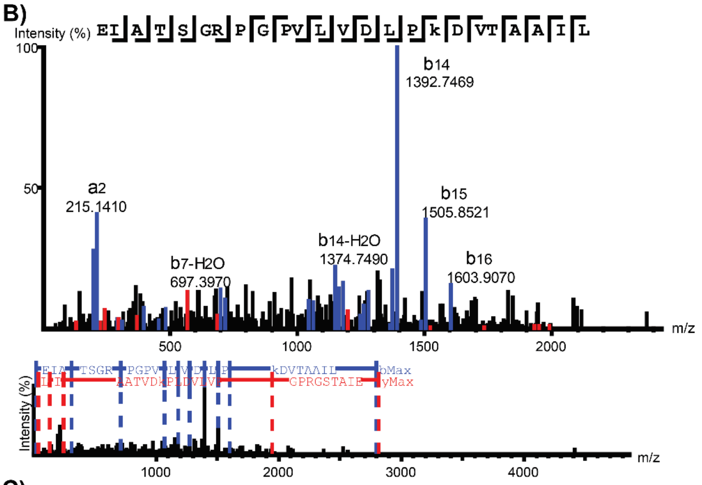

C)

#	b	b-H2O	a	a-NH3	Seq	y	y-H2O	z	#
1	130.0504	112.0399	102.0554	85.0284	E				24
2	243.1345	225.1239	215.1410	198.1230	I	2688.5586	2670.5481	2671.5315	23
3	314.1680	296.1620	286.1766	269.1496	A	2575.4744	2557.4639	2558.4473	22
4	415.2192	397.2040	387.2242	370.1973	T	2504.4373	2486.4268	2487.4102	21
5	502.2513	484.2560	474.2563	457.3000	S	2403.3896	2385.3792	2386.3625	20
6	559.2728	541.2622	531.2778	514.2508	G	2316.3577	2298.3472	2299.3306	19
7	715.4030	697.3970	687.3788	670.3519	R	2259.3362	2241.3257	2242.3091	18
8	812.4266	794.4161	784.4316	767.4047	P	2103.2351	2085.2246	2086.2080	17
9	869.4481	851.4375	841.4531	824.4261	G	2006.1824	1988.0790	1989.1554	16
10	966.5009	948.4903	938.5059	921.4789	P	1949.0940	1931.1504	1932.1541	15
11	1065.5601	1047.5861	1037.5742	1020.5472	V	1852.1082	1834.0977	1835.0812	14
12	1178.6530	1160.6429	1150.6160	1133.6313	L	1753.0397	1734.9520	1736.0127	13
13	1277.7070	1259.7040	1249.7271	1232.6997	V	1639.9556	1621.9451	1622.9286	12
14	1392.7469	1374.7490	1364.7679	1347.7267	D	1540.8872	1522.8820	1523.8602	11
15	1505.8521	1487.8690	1477.8378	1460.8108	L	1425.8602	1407.8497	1408.8333	10
16	1602.9041	1584.8750	1574.8905	1557.8635	P	1312.7762	1294.7657	1295.7493	9
17	2116.2058	2098.1953	2088.2109	2071.1838	K(+385.23)	1215.7234	1197.6650	1198.6964	8
18	2231.2327	2213.2222	2203.2378	2186.2107	D	702.4032	684.3800	685.3762	7
19	2330.3010	2312.2905	2302.3062	2285.2791	V	587.3763	569.3460	570.3493	6
20	2431.3486	2413.3381	2403.3538	2386.3267	T	488.3079	470.2973	471.2809	5
21	2502.3857	2484.3752	2474.3909	2457.3638	A	387.2602	369.1800	370.2332	4
22	2573.4229	2555.4124	2545.4280	2528.4009	A	316.2231	298.1430	299.1961	3
23	2686.5071	2668.4966	2658.5122	2641.4851	I	245.1880	227.1820	228.1590	2
24					L	132.1030	114.0913	115.0749	1

D)

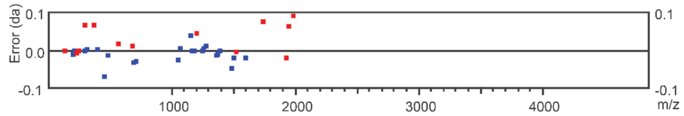

Figure S7: Peptide mapping of *S. cerevisiae* ALS treated with **1** localizes the site of covalent modification to the SGRPGPVLVDLPKDVTAAIL peptide. **A)** MS spectrum of the target peptide. **B)** Annotated MS2 spectrum of the target peptide. **C)** Peptide table from MS^2 analysis of the target peptide. **D)** Error map of the MS^2 analysis.

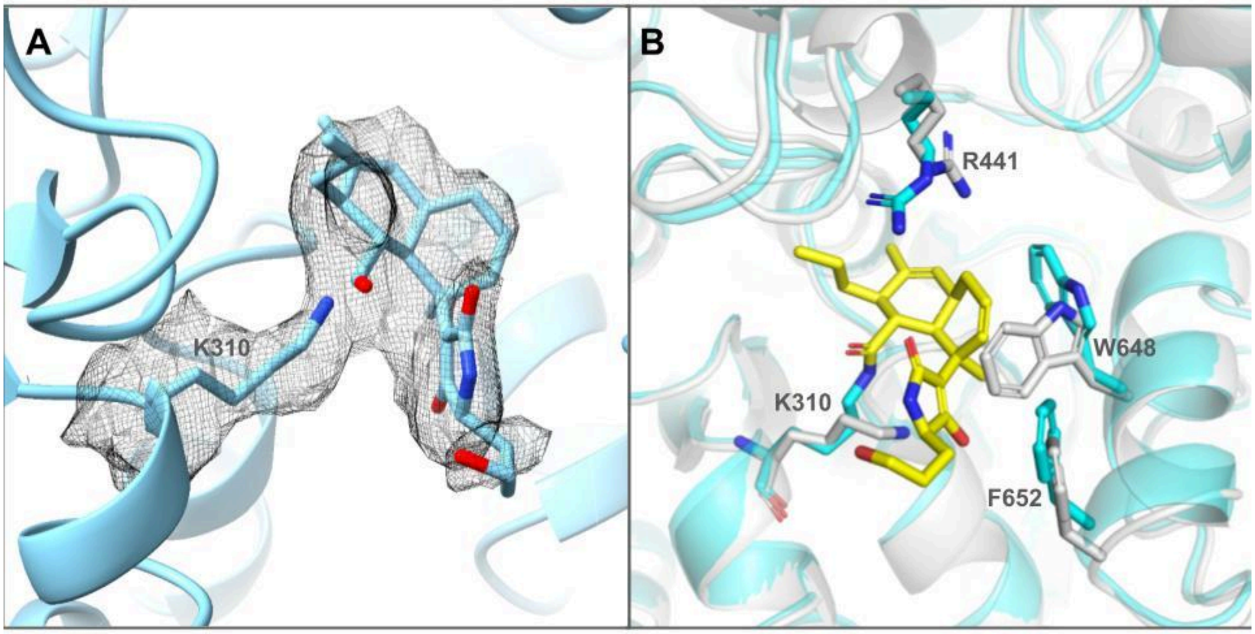

Figure S8: cryoEM density of compound **1** and the conformational changes it causes at the *Af*ALS substrate channel. **A)** The continuous cryoEM density between Af ALS K310 and compound 1 indicates a covalent bond formed between them. **B)** the superposition of Apo (gray) and compound **1** (yellow)-bound (cyan) Af ALS structures.

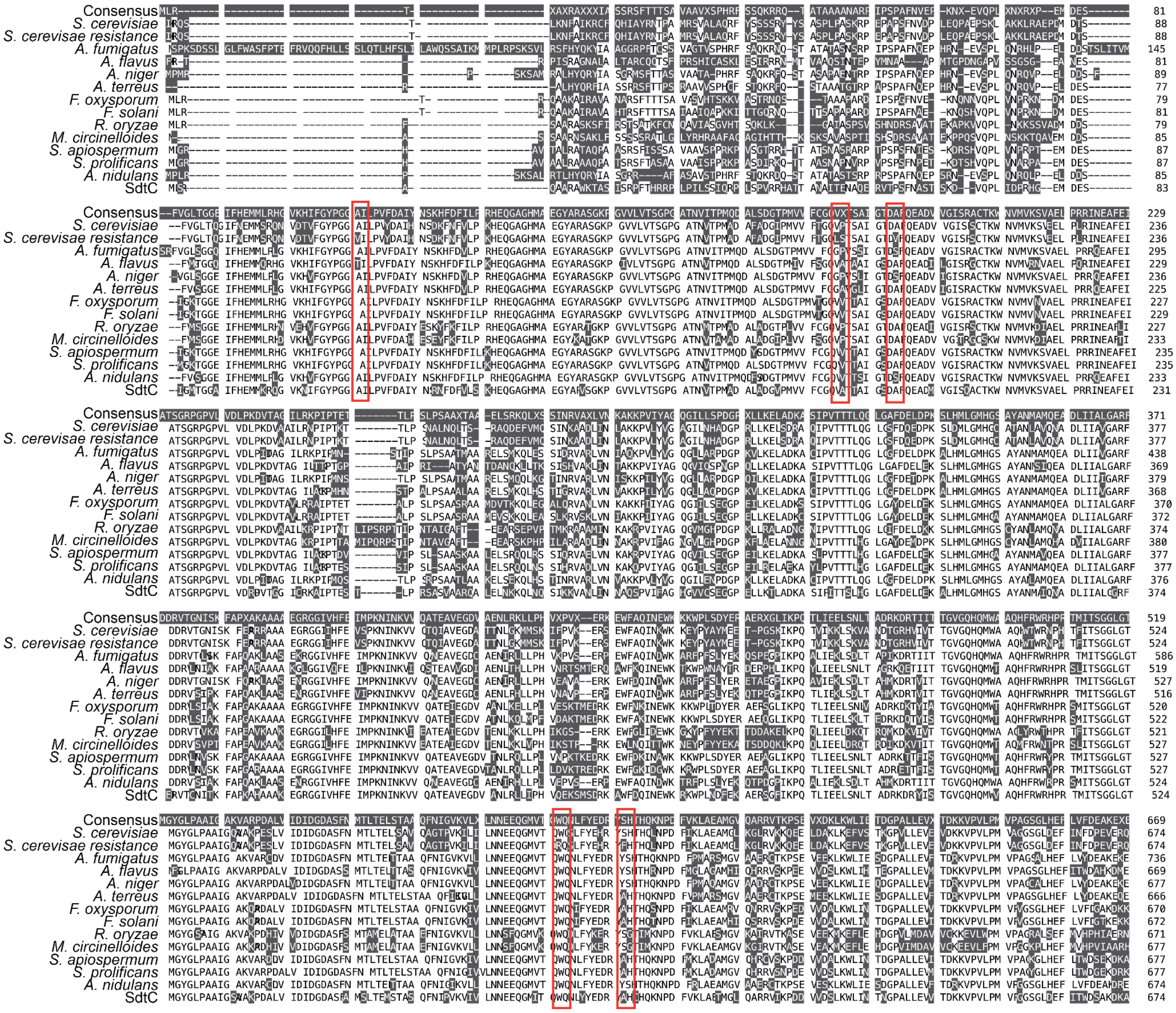

Figure S9: Multiple sequence alignment of ALS proteins derived from all fungi included in the antifungal screening panel in this study. Positions where mutations were observed in forward genetic screens of 1 are highlighted with orange boxes.

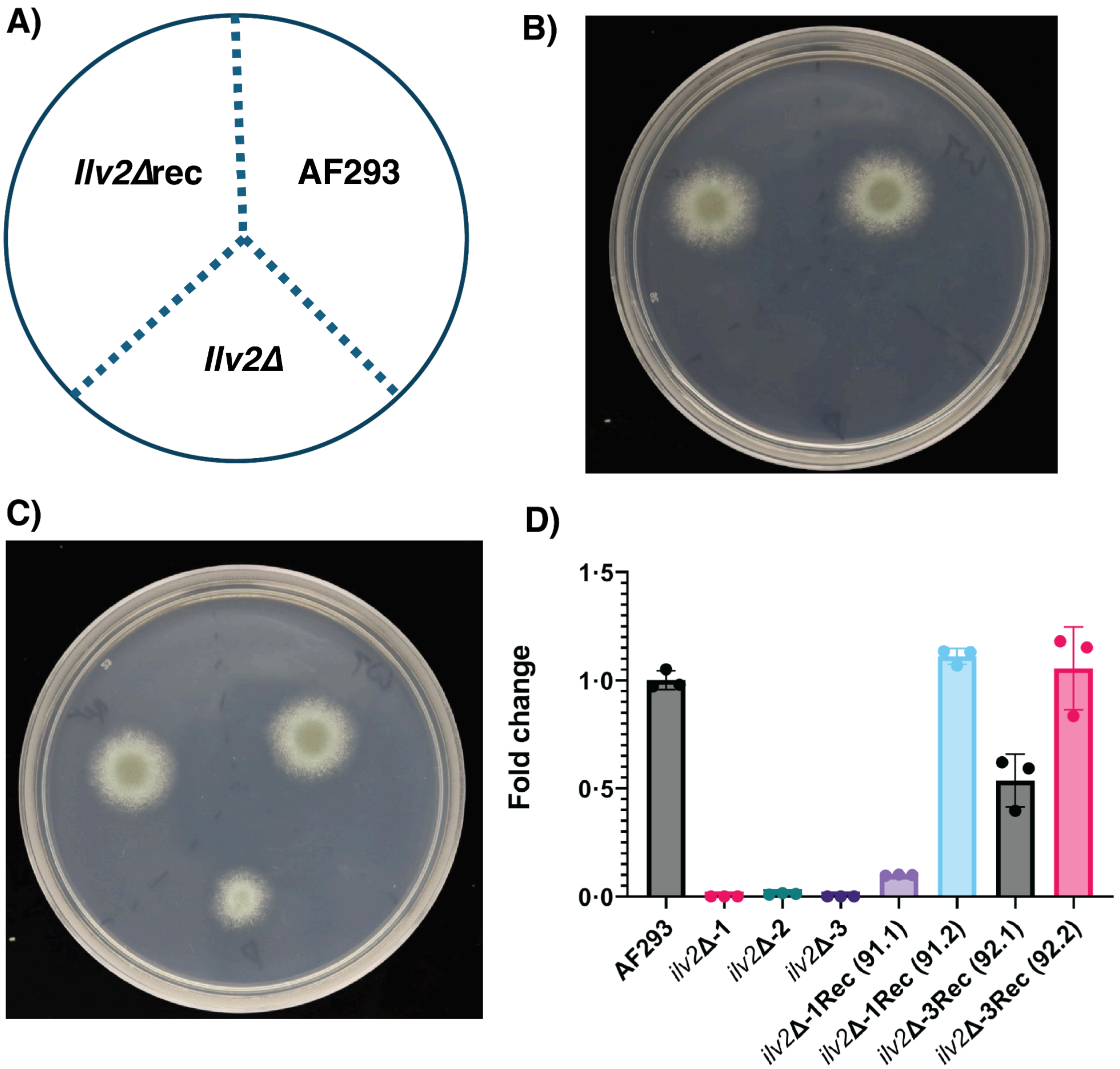

Figure S10: Validation of *A. fumigatus* mutants A) Plate map for growth plating experiments **B)** Growth of *A. fumigatus* strains on glucose minimal medium with no supplemented amino acids. **C)** Growth of strains on glucose minimal medium supplemented with 5 mM each of valine and isovaline. All images taken 48 hrs after plating of 1000 spores per strain **D)** RT-qPCR validation of *ilv2Δ* and *ilv2Δ*Rec strains*.* Clones *ilv2Δ-1* and *ilv2Δ*Rec (91.2) were taken forward for studies in mice.

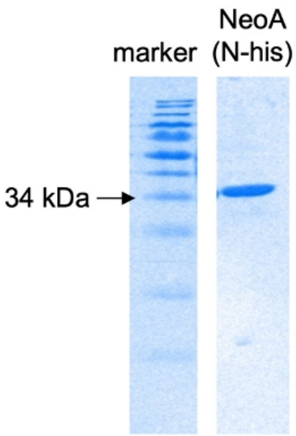

Figure S11: SDS page gel of recombinant NeoA

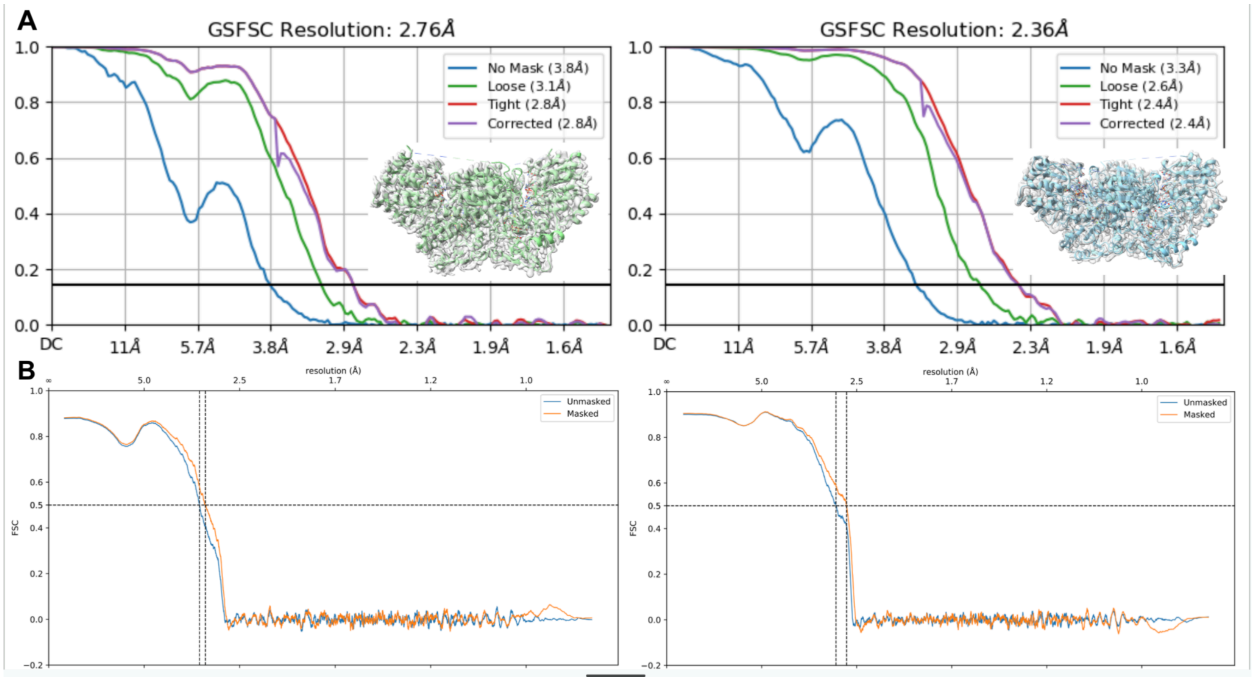

Figure S12: Quality of the cryo-EM reconstruction of the Apo and 1-bound *Af*ALS. A) The Fourier shell correlation (FSC) curves of the cryo-EM reconstructions. An overview of the model docked cryoEM map was shown as an inset. Left, Apo *Af*ALS; Right, **1**-bound *Af*ALS. **B)** The model-to-map FSC curves of Apo *Af*ALS (left) and **1**-bound AfALS (right) structures.

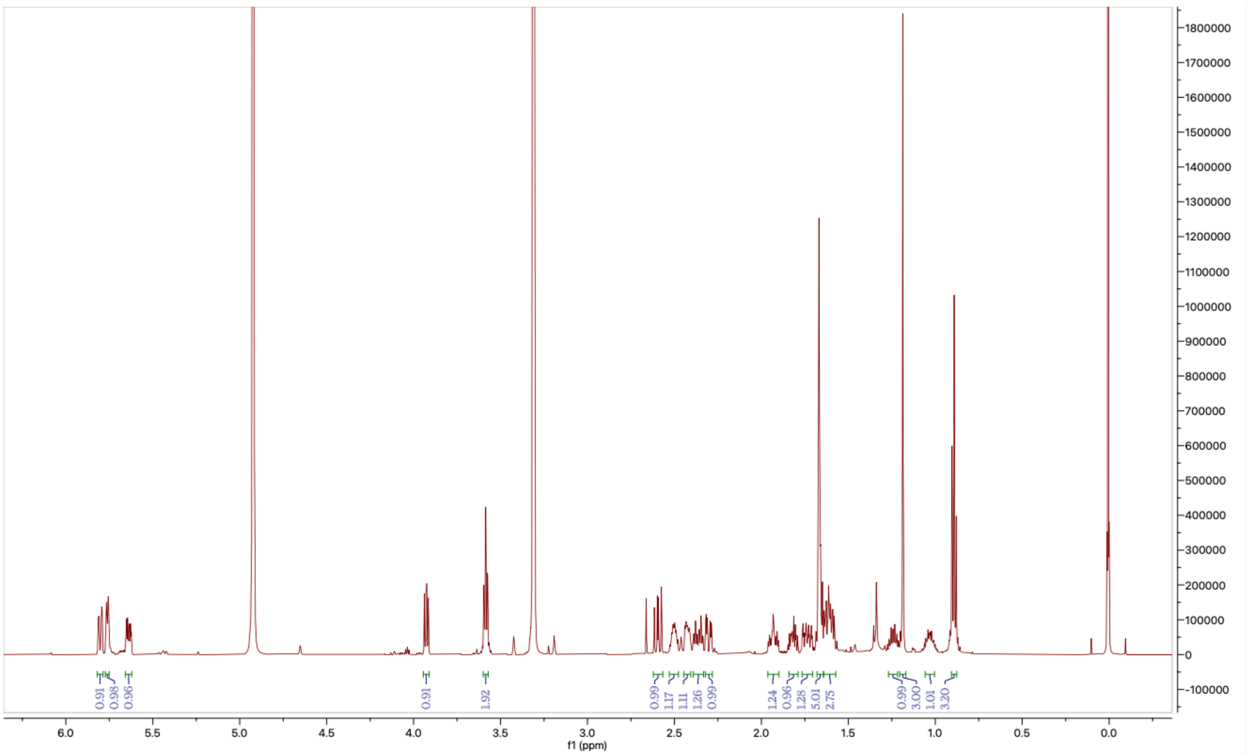

Figure S13: The ¹H NMR Spectrum of **1** in MeOD (600 MHz)

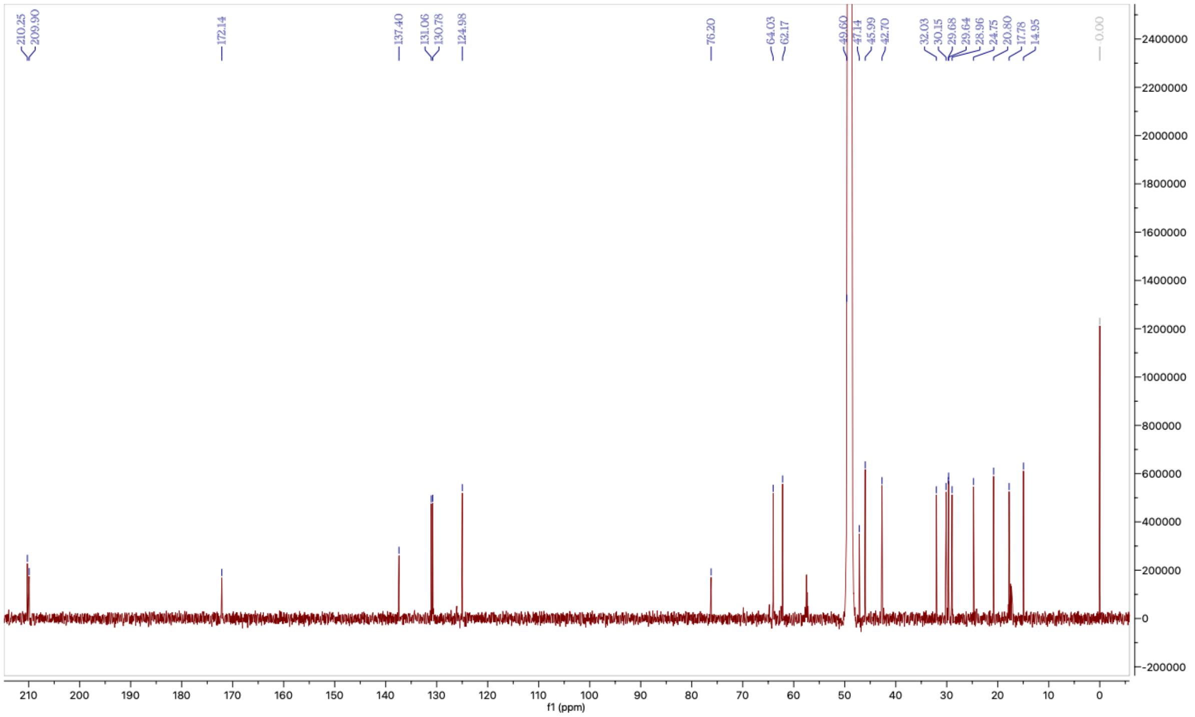

Figure S14: The ¹³C NMR Spectrum of **1** in MeOD (150 MHz)

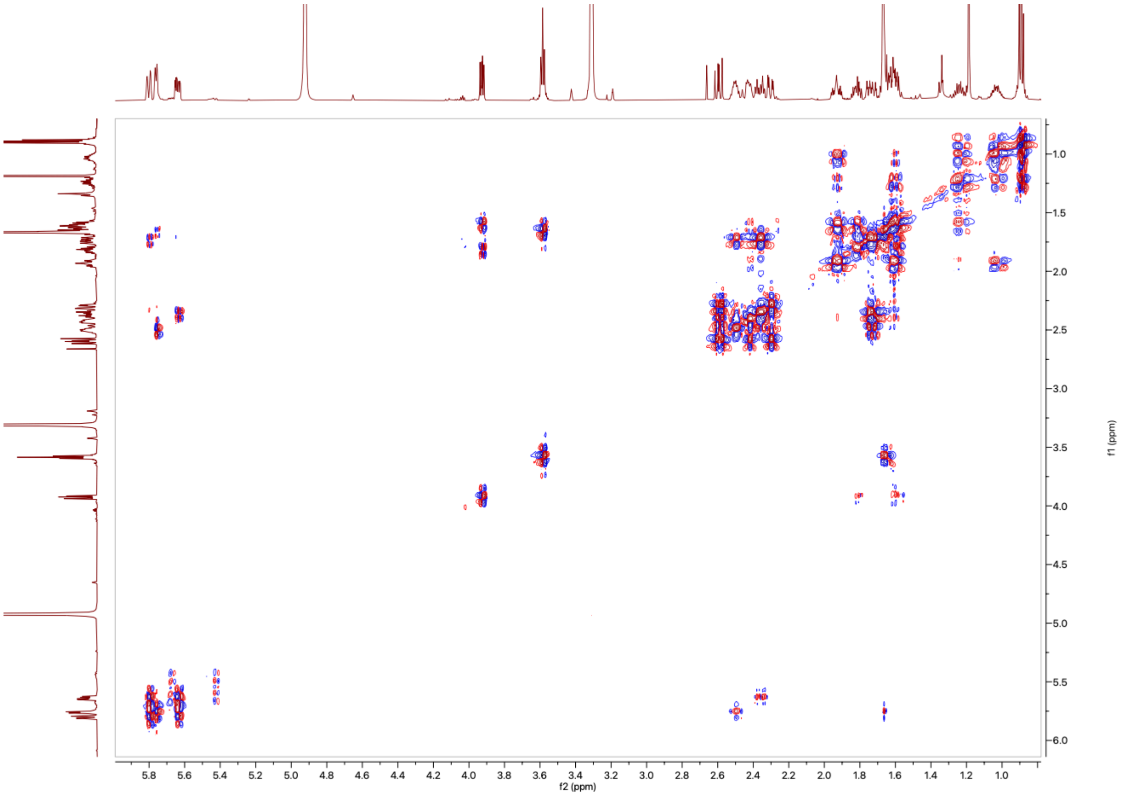

Figure S15: The ^{1}H-^{1}H COSY NMR Spectrum of **1** in MeOD (600 MHz)

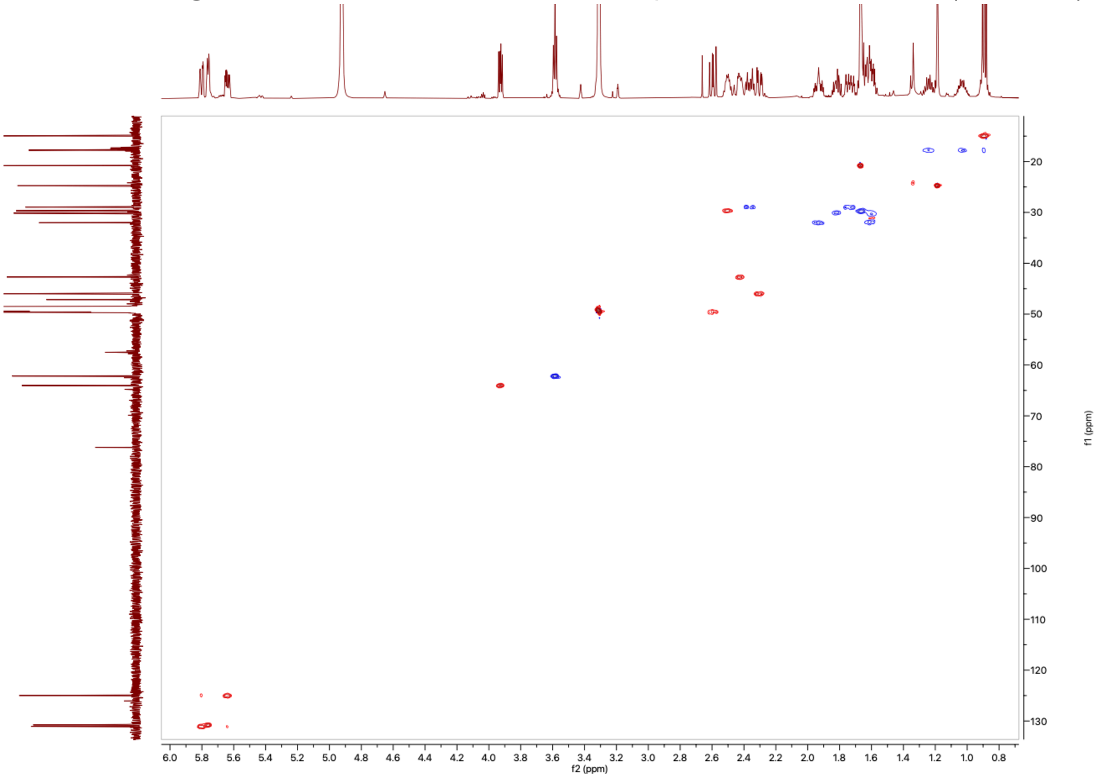

Figure S16: The ^{1}H-^{13}C HSQC NMR Spectrum of **1** in MeOD (600 MHz)

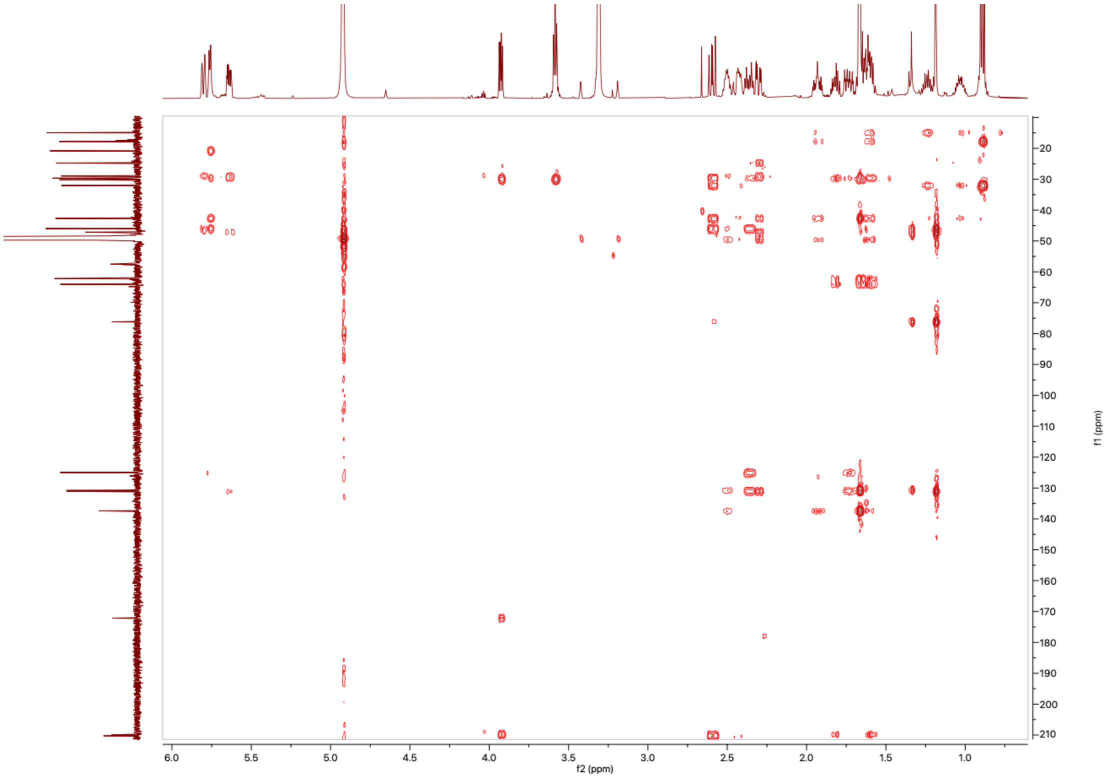

Figure S17: The ^{1}H-^{13}C HMBC NMR Spectrum of **1** in MeOD (600 MHz)

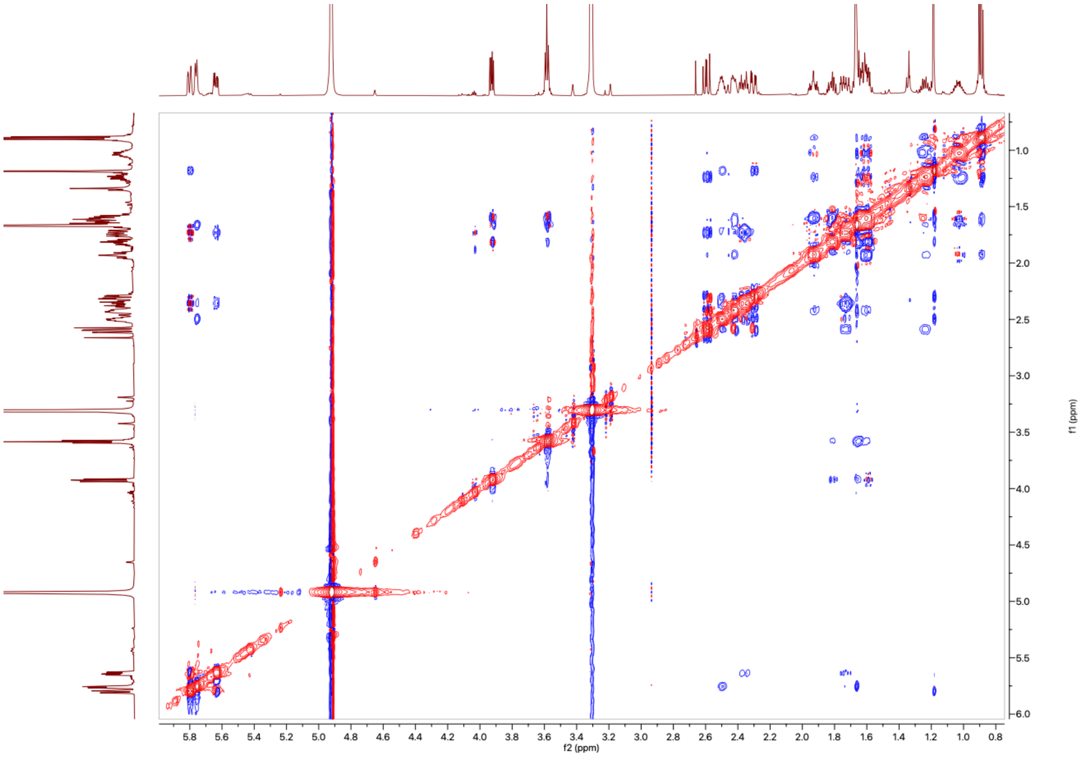

Figure S18: The ^{1}H-^{1}H NOESY NMR Spectrum of **1** in MeOD (600 MHz)

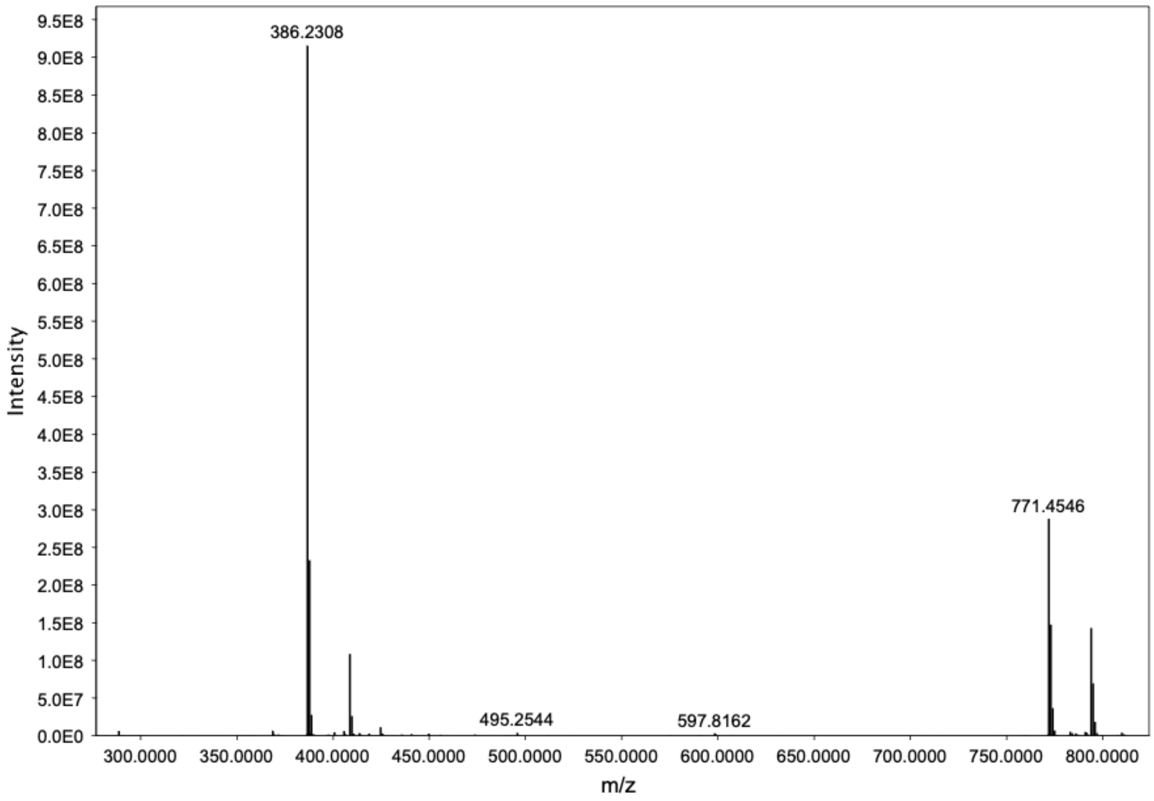

Figure S19: HRMS spectrum of **1**

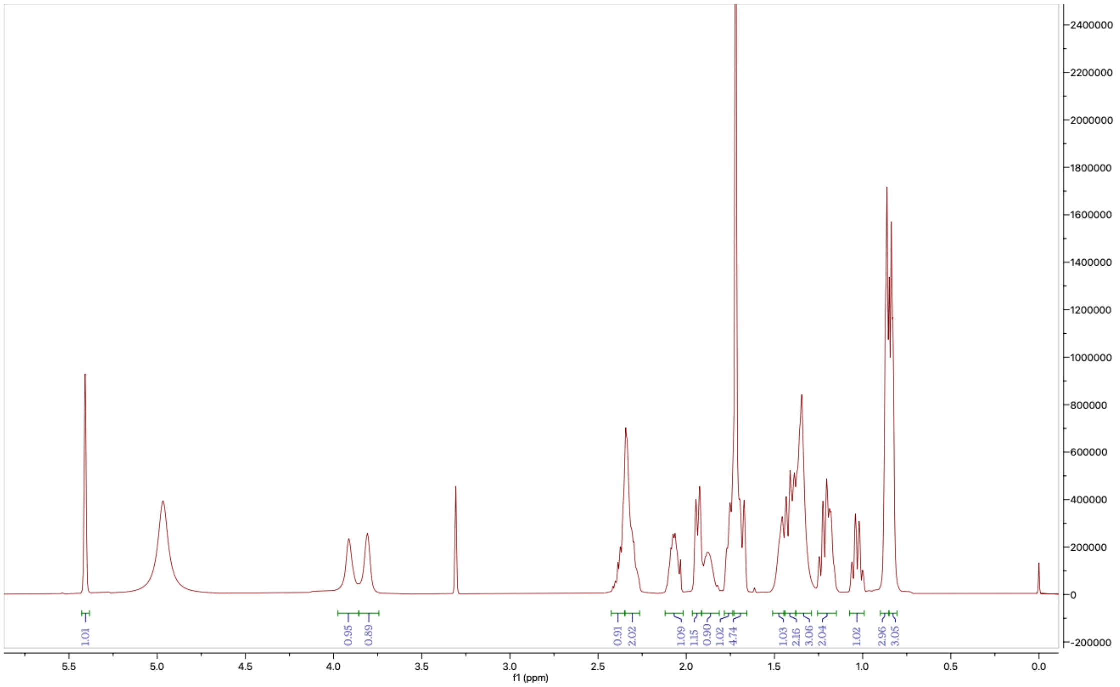

Figure S20: The ^{1}H NMR Spectrum of **10** in MeOD (600 MHz)

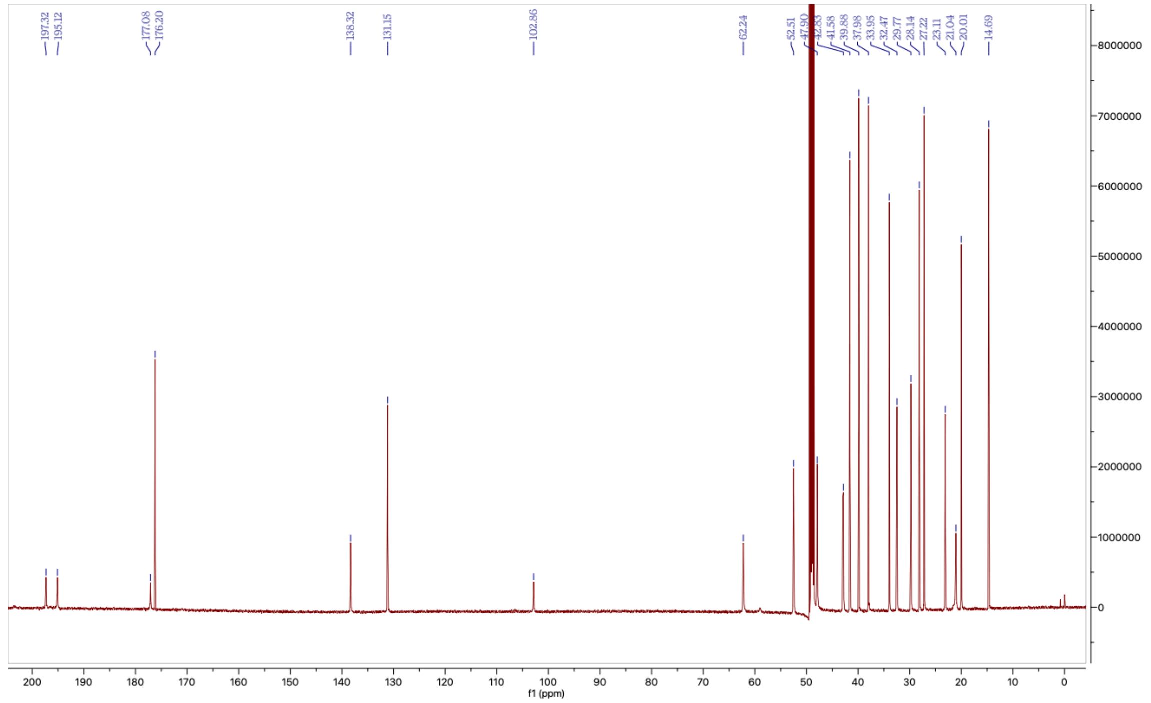

Figure S21: The ^{13}C NMR Spectrum of **10** in MeOD (600 MHz)

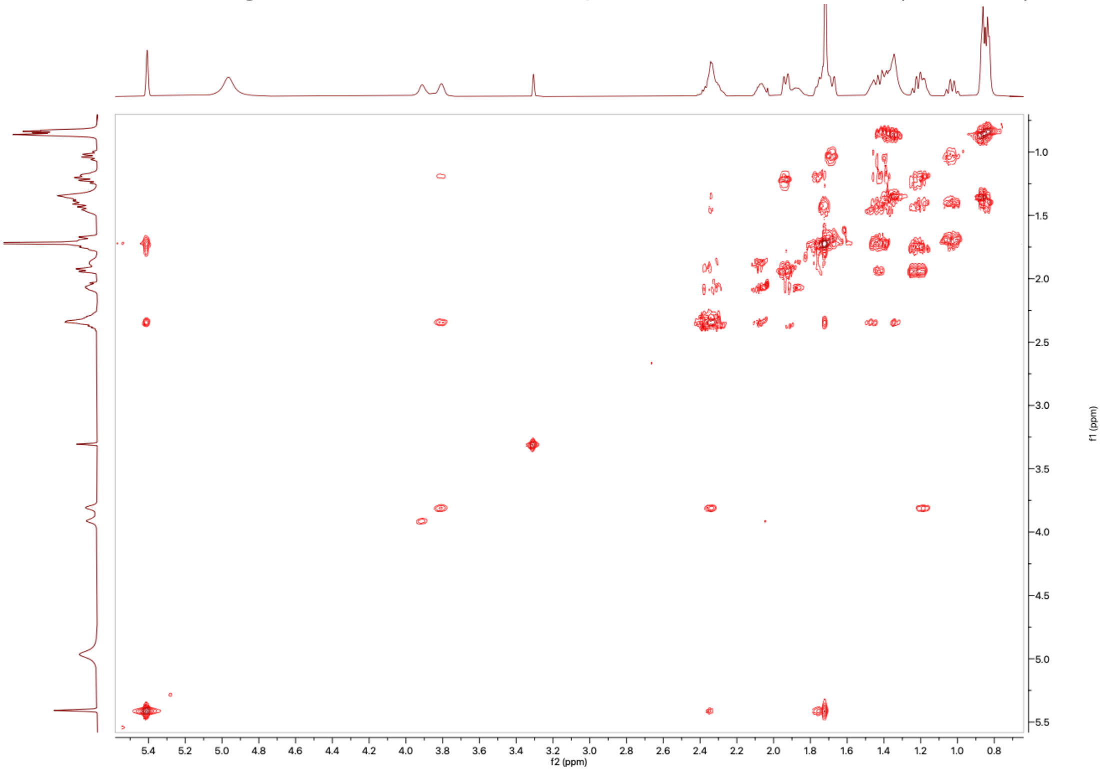

Figure S22: The ^{1}H-^{1}H COSY NMR Spectrum of **10** in MeOD (600 MHz)

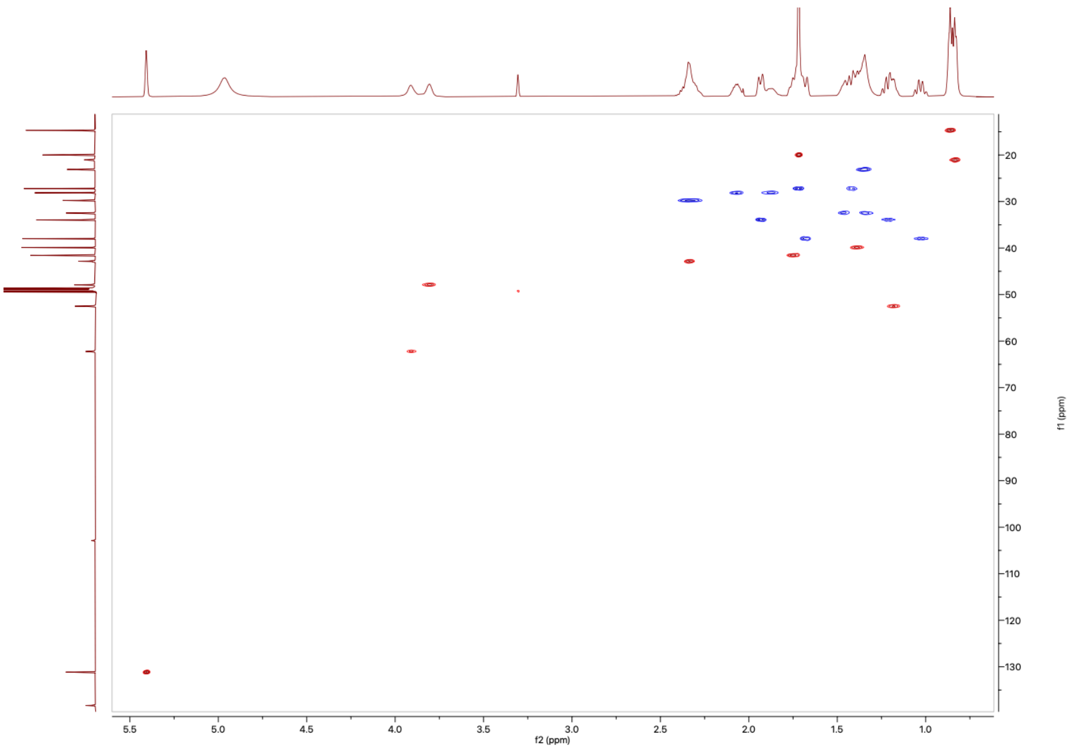

Figure S23: The ^1H-^{13}C HSQC NMR Spectrum of **10** in MeOD (600 MHz)

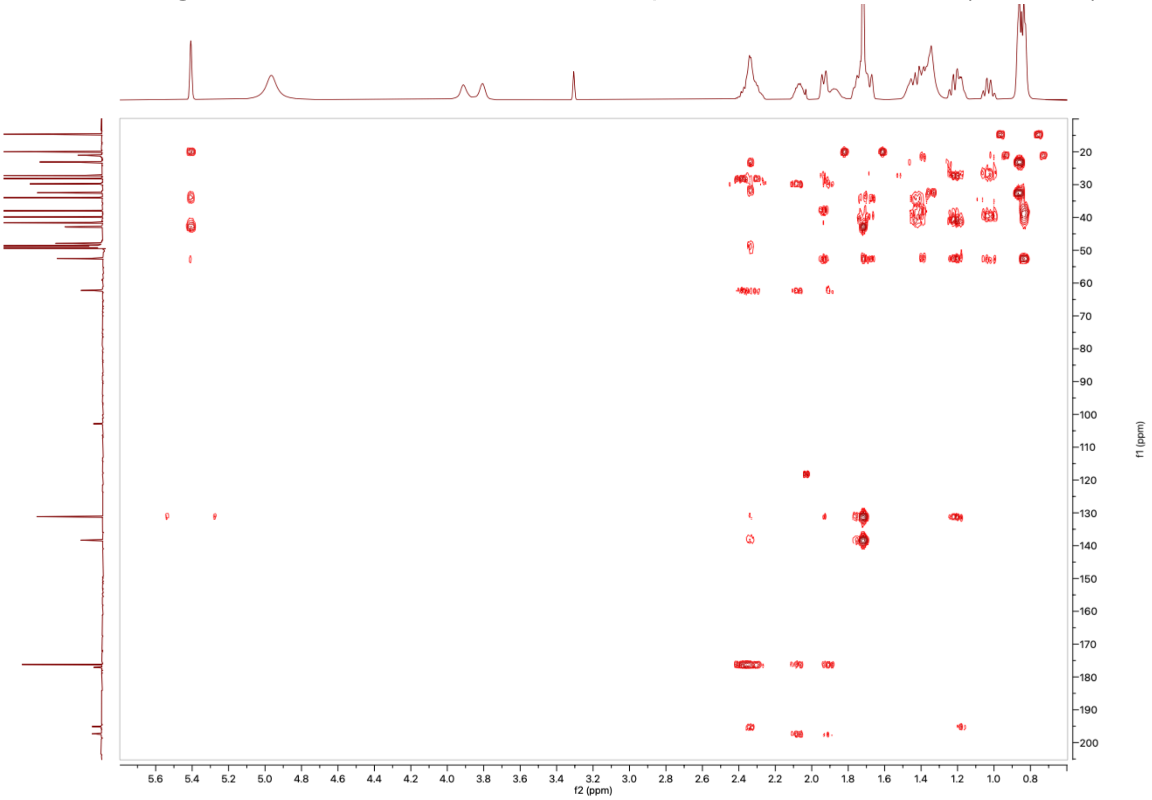

Figure S24: The ^1H-^{13}C HMBC NMR Spectrum of **10** in MeOD (600 MHz)

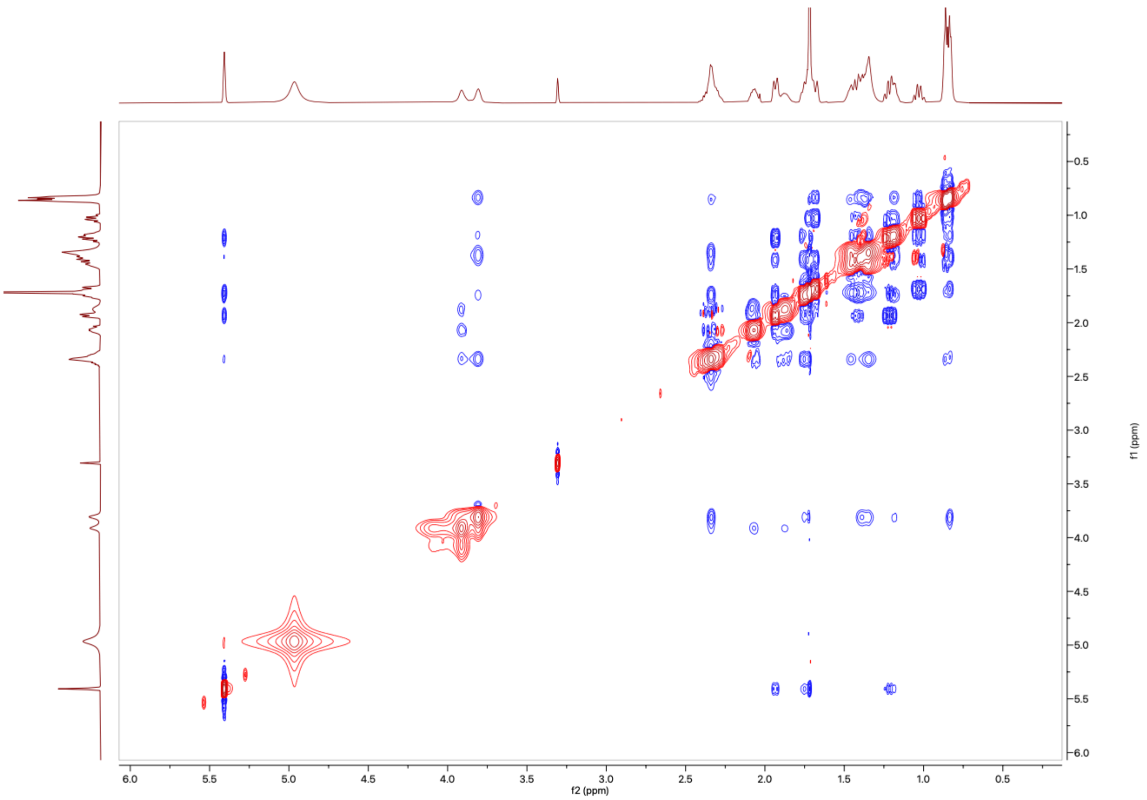

Figure S25: The ^1H-^1H NOESY NMR Spectrum of **10** in MeOD (600 MHz)

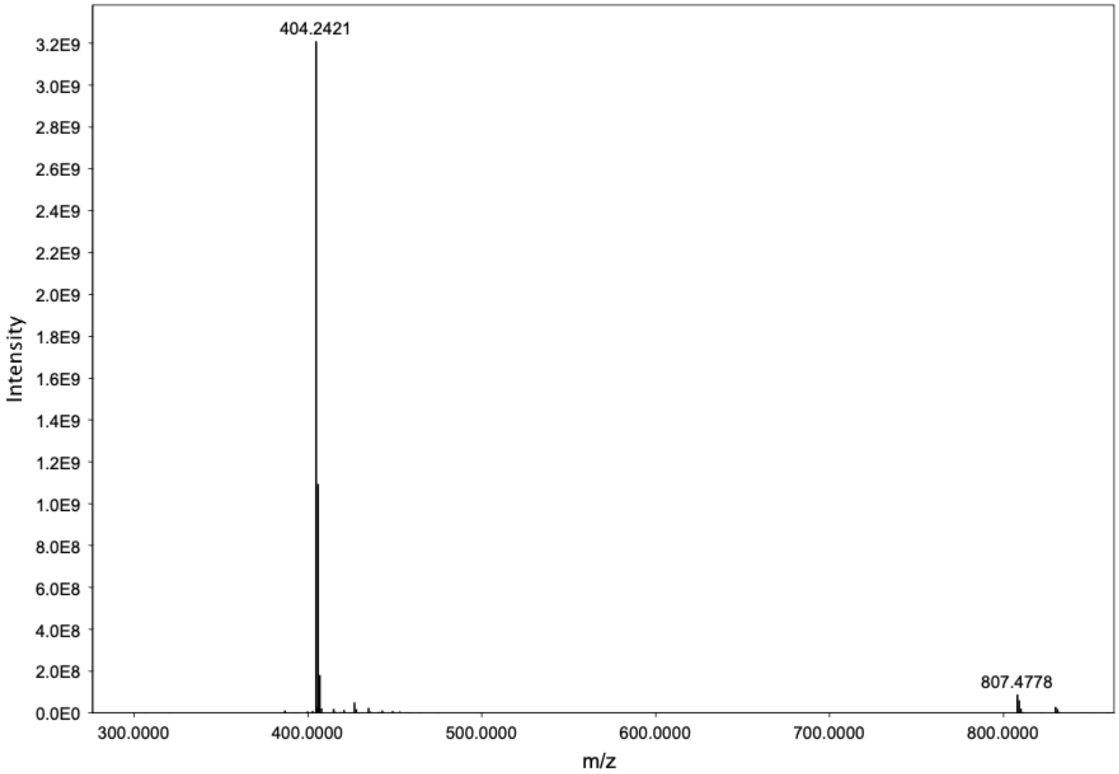

Figure S26: HRMS spectrum of **10**

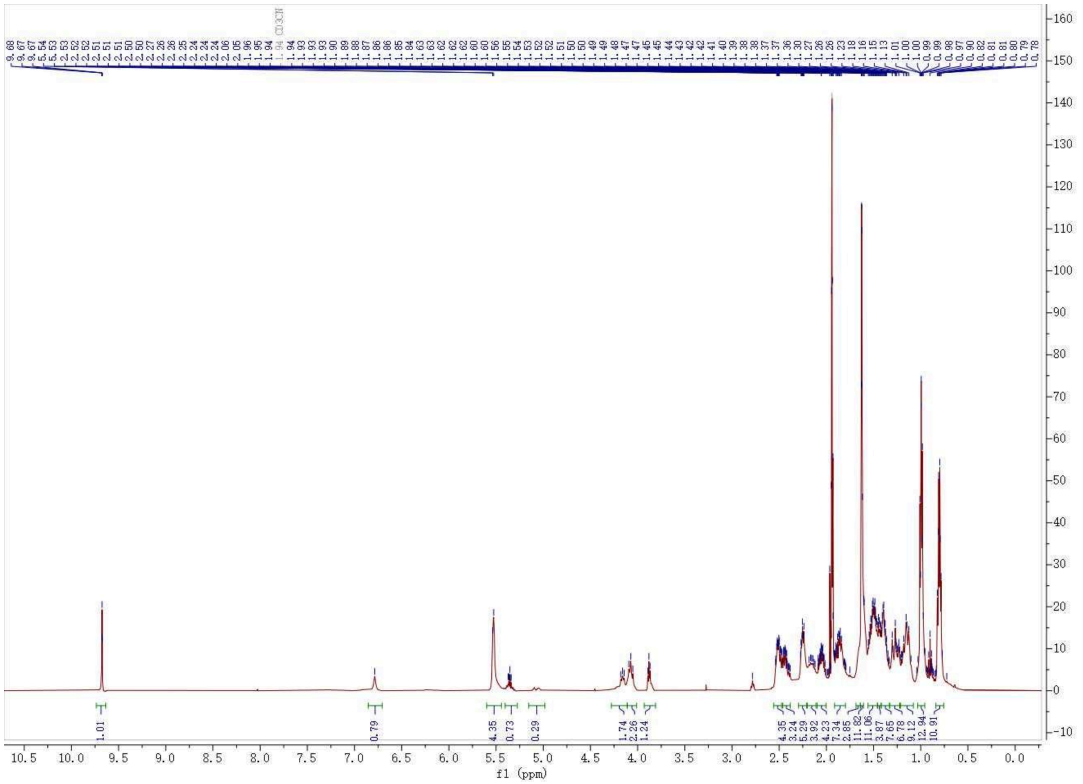

Figure S27: The ^{1}H NMR Spectrum of **11** in $CDCN_3$ (500 MHz)

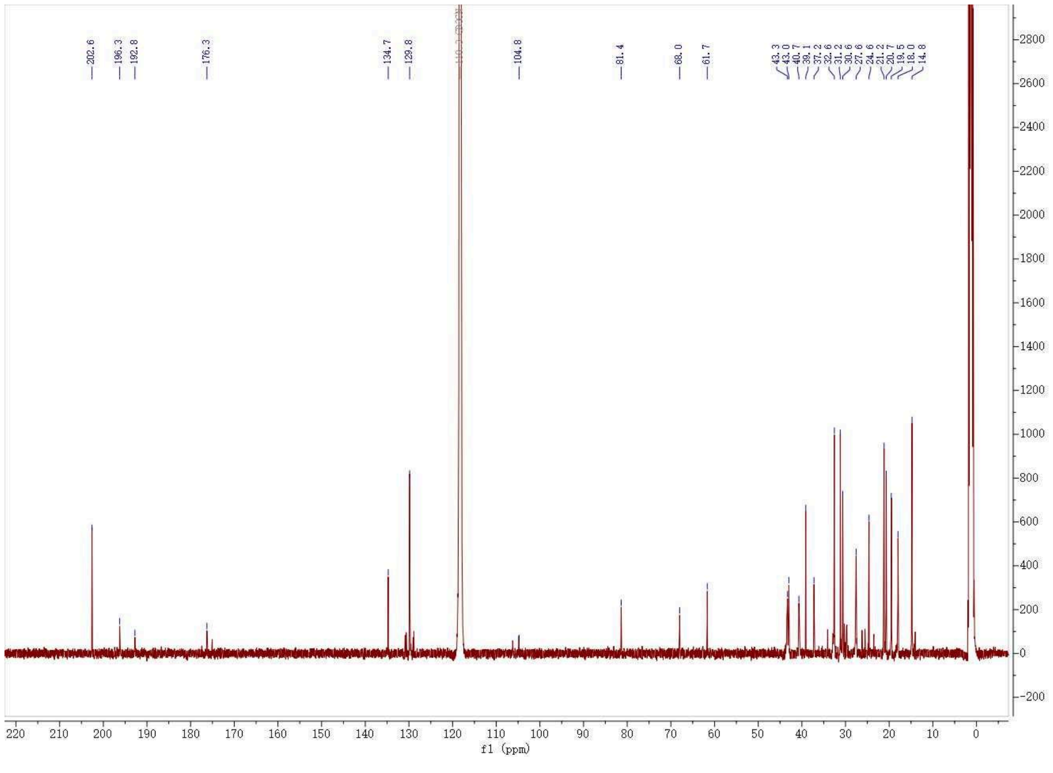

Figure S28: The ^{13}C NMR Spectrum of **11** in $CDCN_3$ (500 MHz)

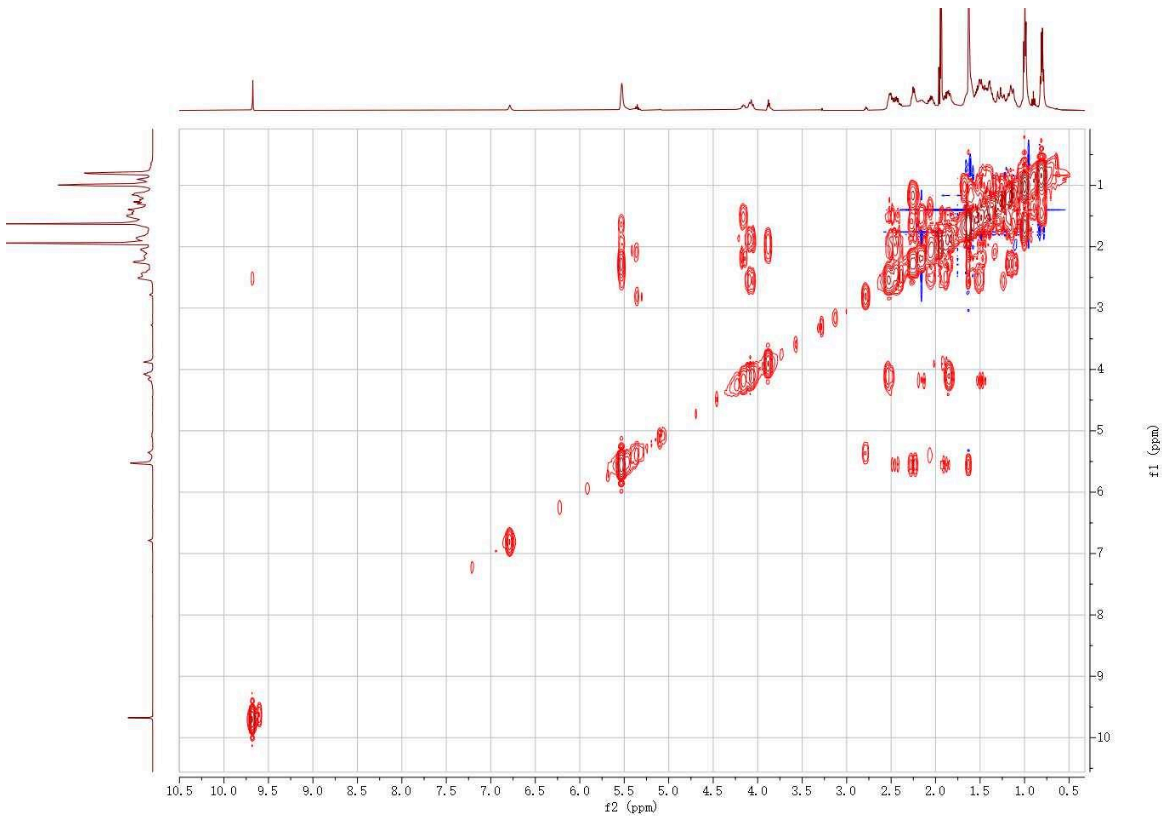

Figure S29: The ¹H-¹H COSY NMR Spectrum of **11** in $CDCN_3$ (500 MHz)

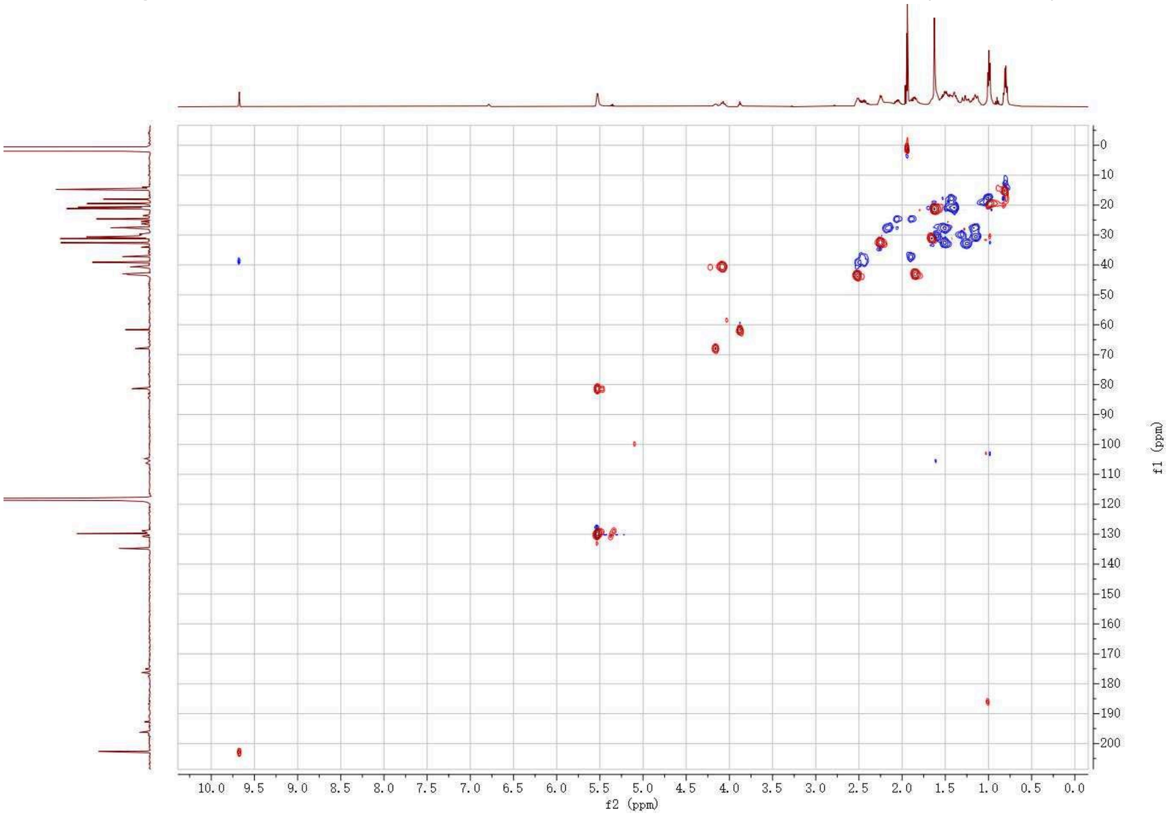

Figure S30: The ¹H-¹³C HSQC NMR Spectrum of **11** in $CDCN_3$ (500 MHz)

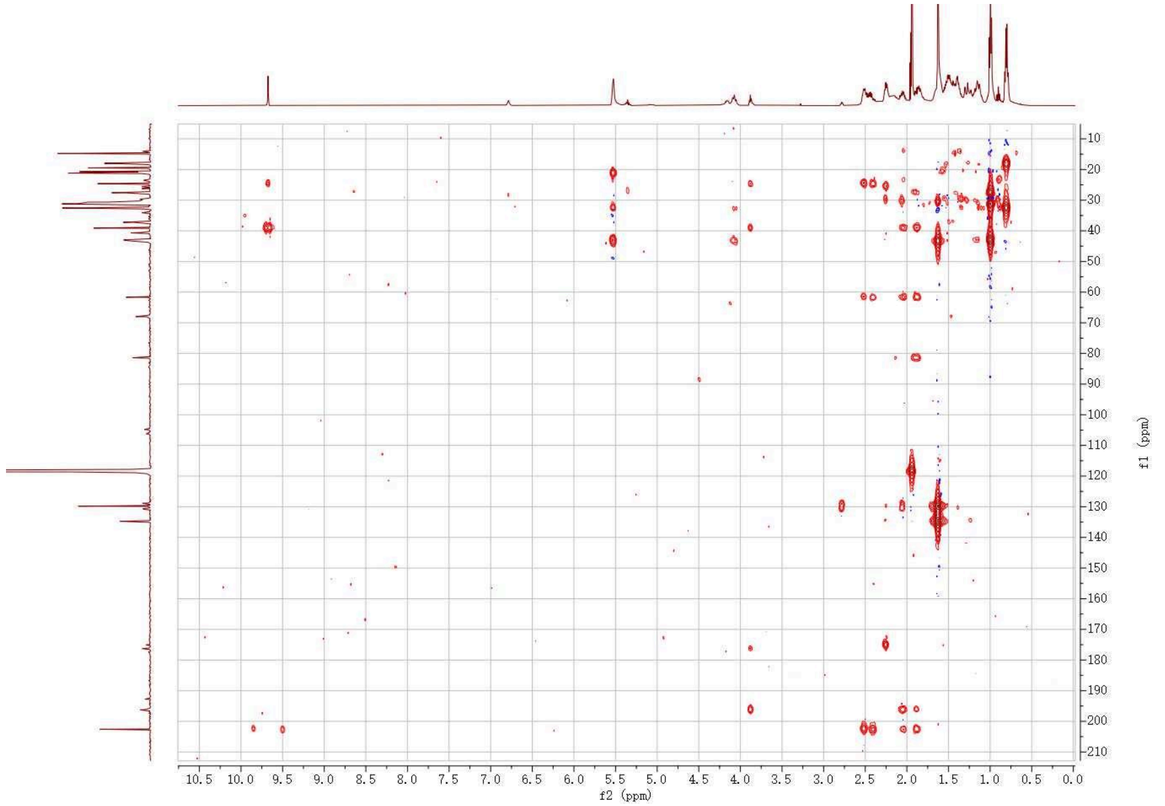

Figure S31: The ^{1}H-^{13}C HMBC NMR Spectrum of **11** in $CDCN_3$ (500 MHz)

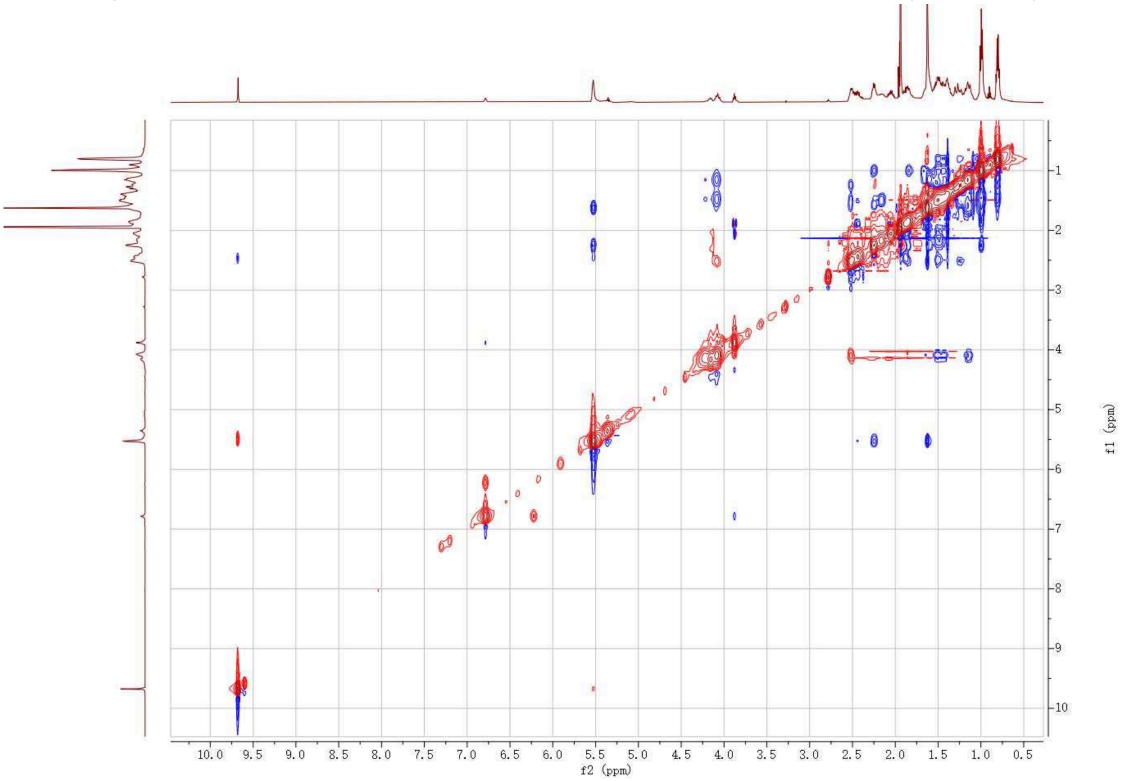

Figure S32: The ^{1}H-^{1}H NOESY NMR Spectrum of **11** in $CDCN_3$ (500 MHz)

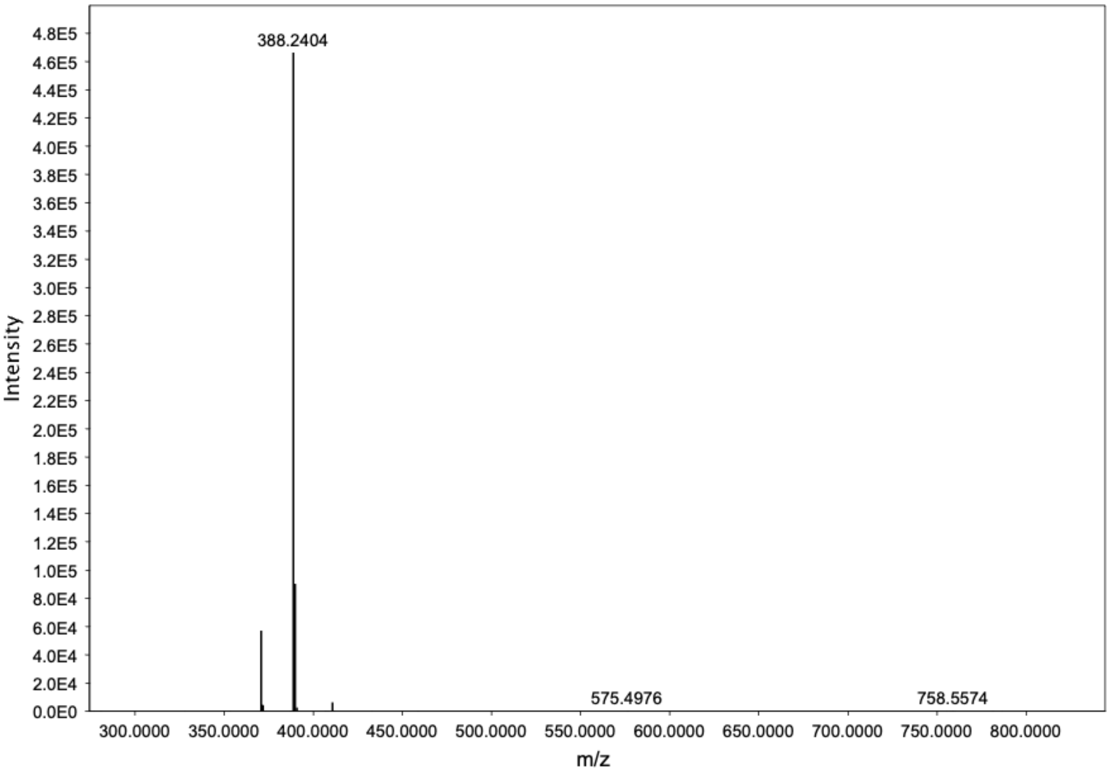

Figure S33: HRMS Spectrum of **11**

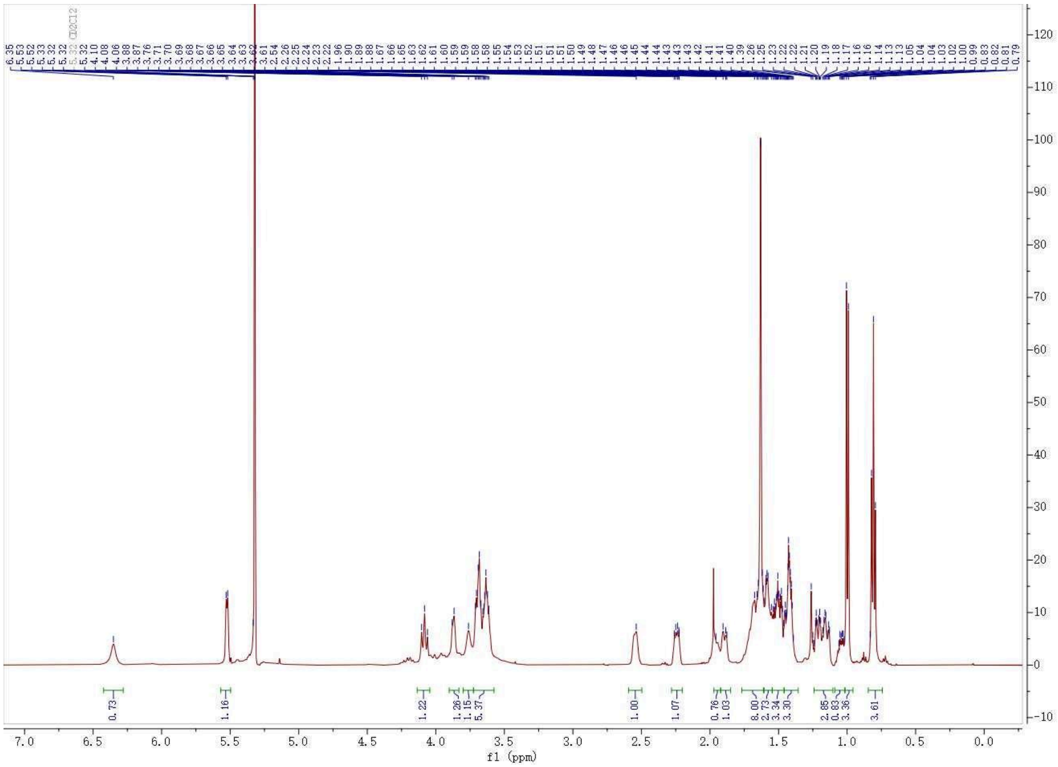

Figure S34: The ¹H NMR Spectrum of **12** in CD_2Cl_2 (500 MHz)

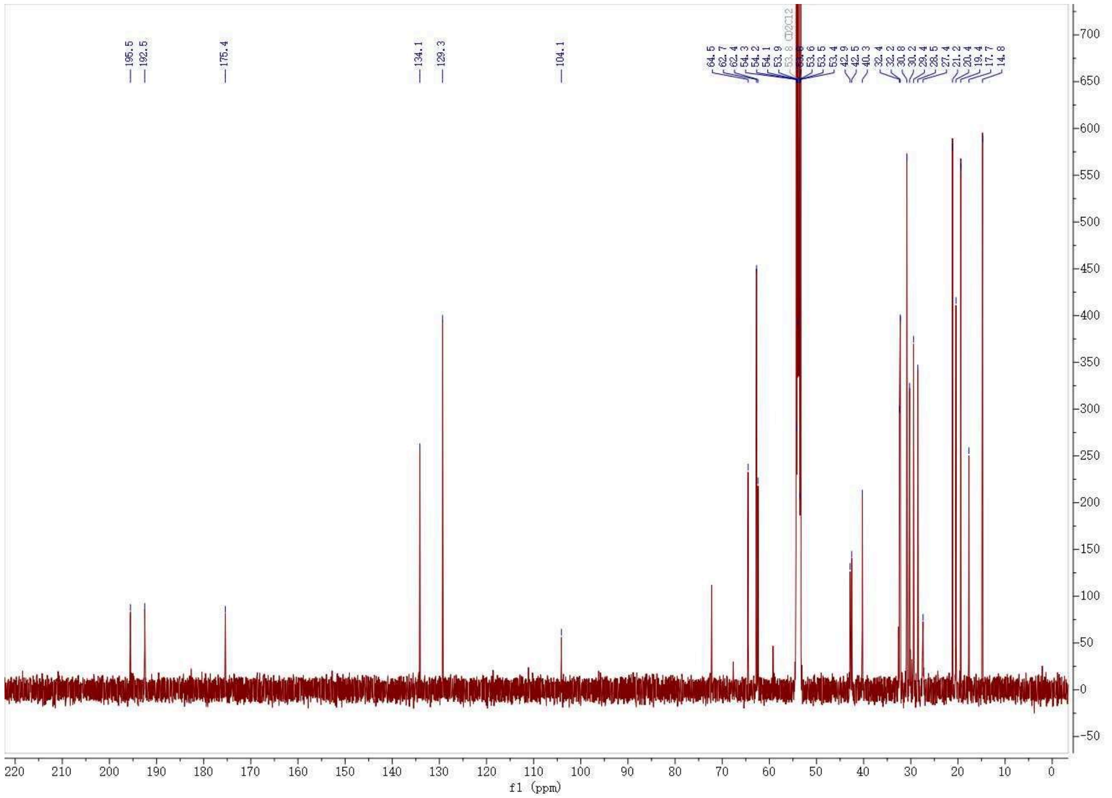

Figure S35: The ¹³C NMR Spectrum of **12** in CD_2Cl_2 (500 MHz)

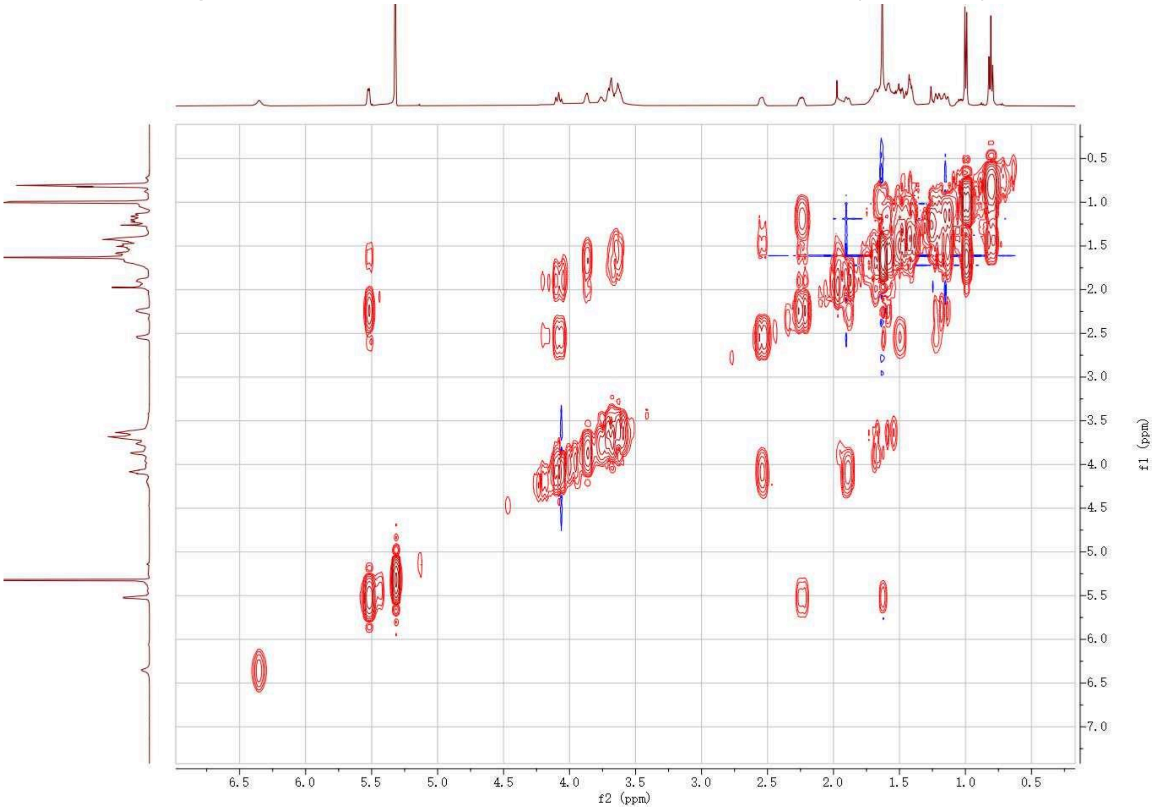

Figure S36: The ¹H-¹H COSY NMR Spectrum of **12** in CD_2Cl_2(500 MHz)

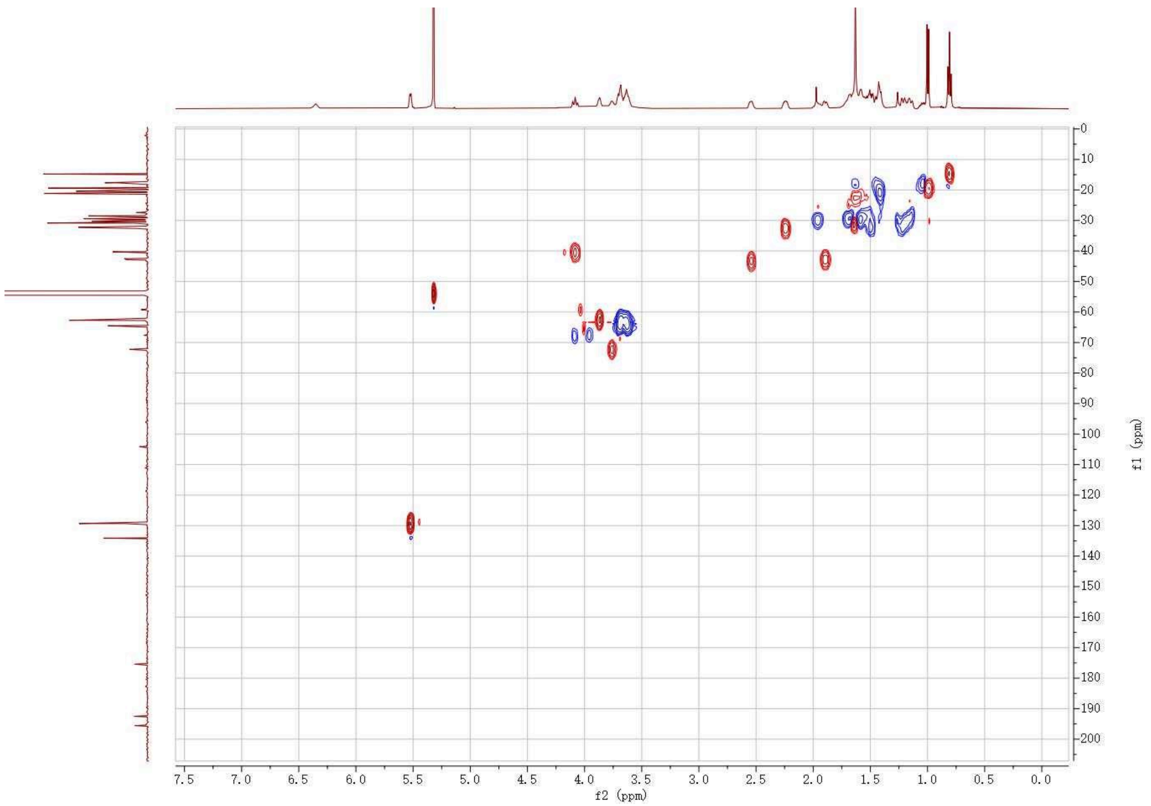

Figure S37: The ^{1}H-^{13}C HSQC NMR Spectrum of **12** in CD_2Cl_2 (500 MHz)

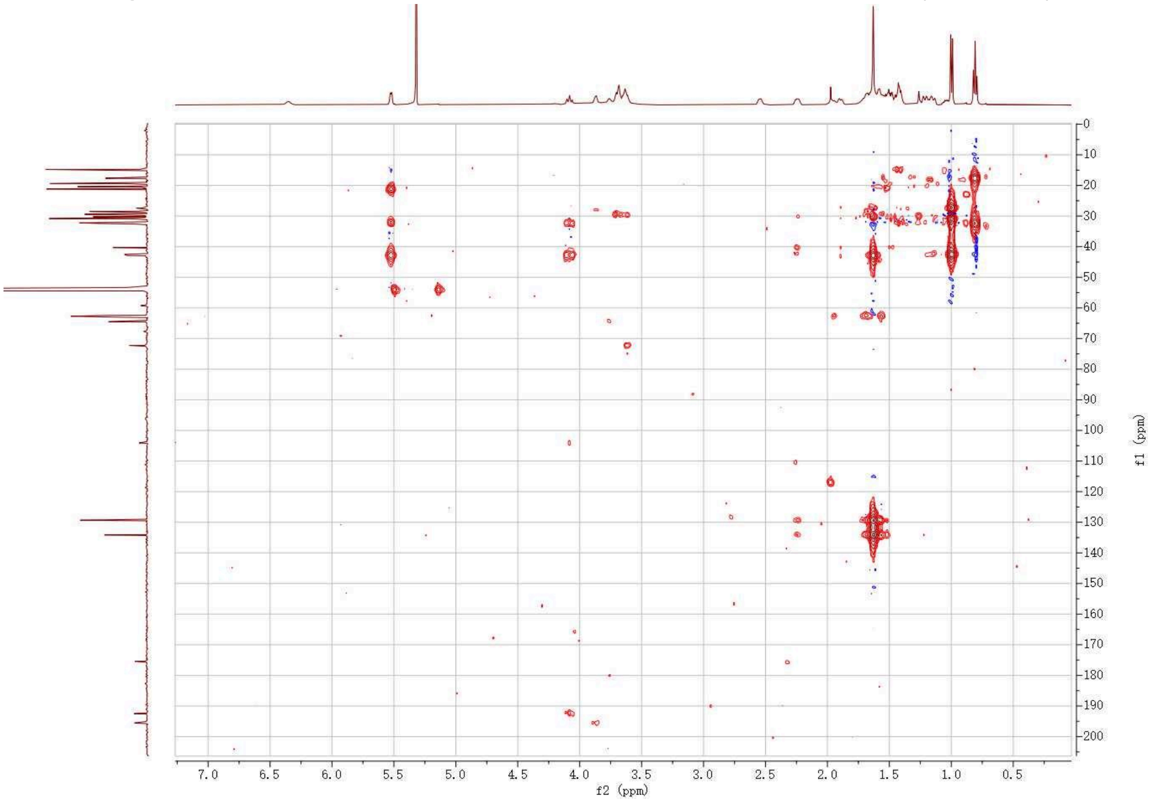

Figure S38: The ^{1}H-^{13}C HMBC NMR Spectrum of **12** in CD_2Cl_2 (500 MHz)

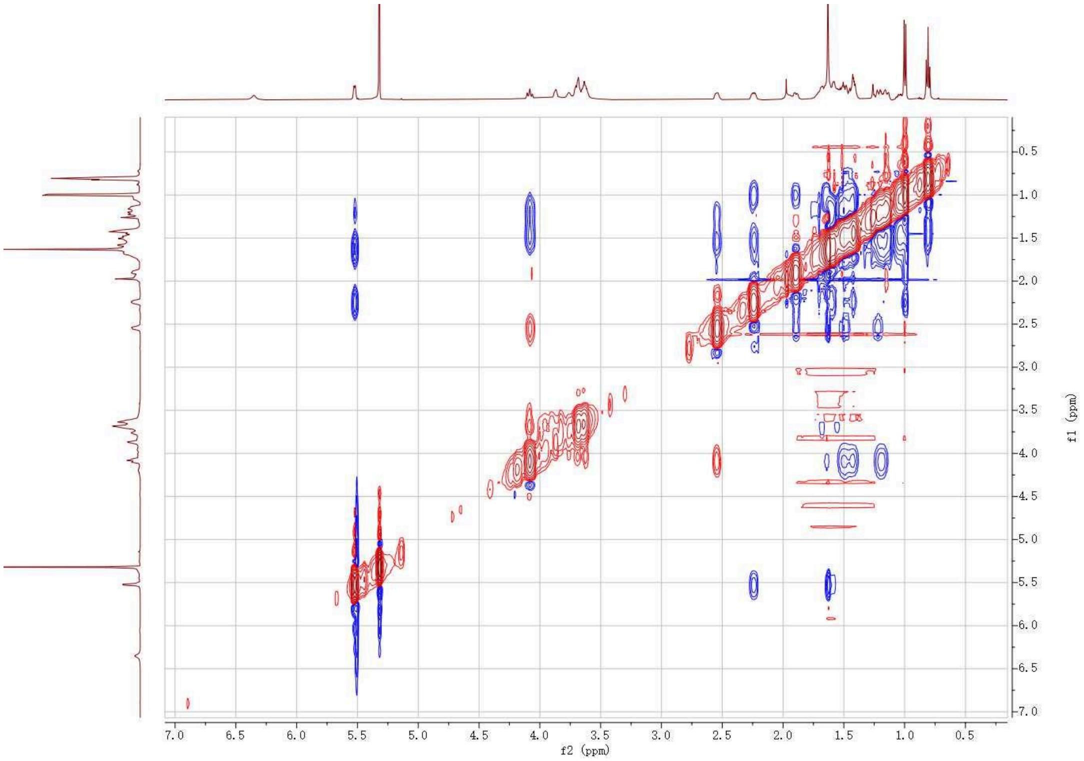

Figure S39: The ^{1}H-^{1}H NOESY NMR Spectrum of **12** in CD_2Cl_2 (500 MHz)

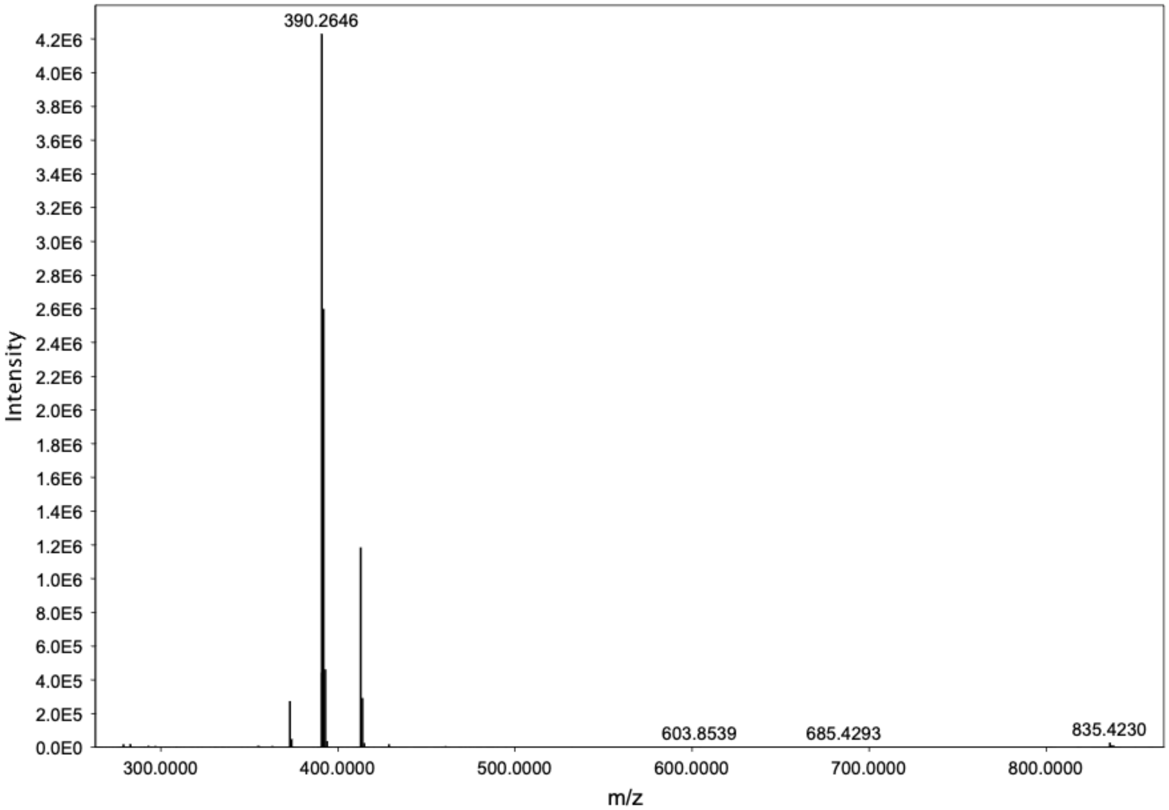

Figure S40: HRMS spectrum of **12**

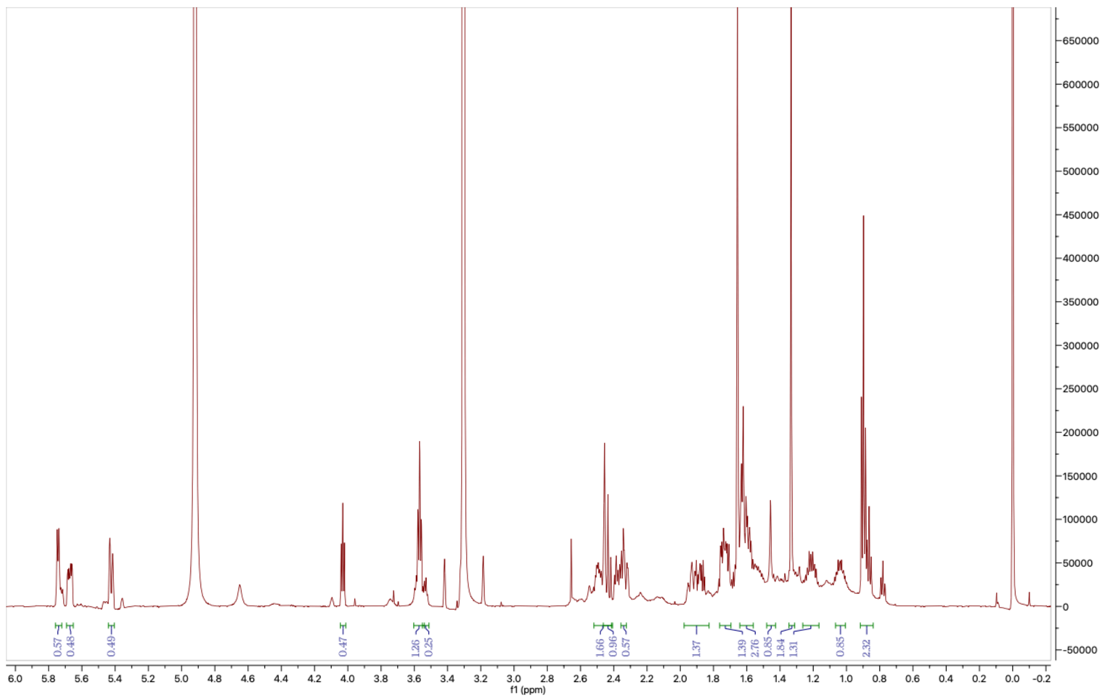

Figure S41: The ^{1}H NMR Spectrum of **13** in MeOD (600MHz)

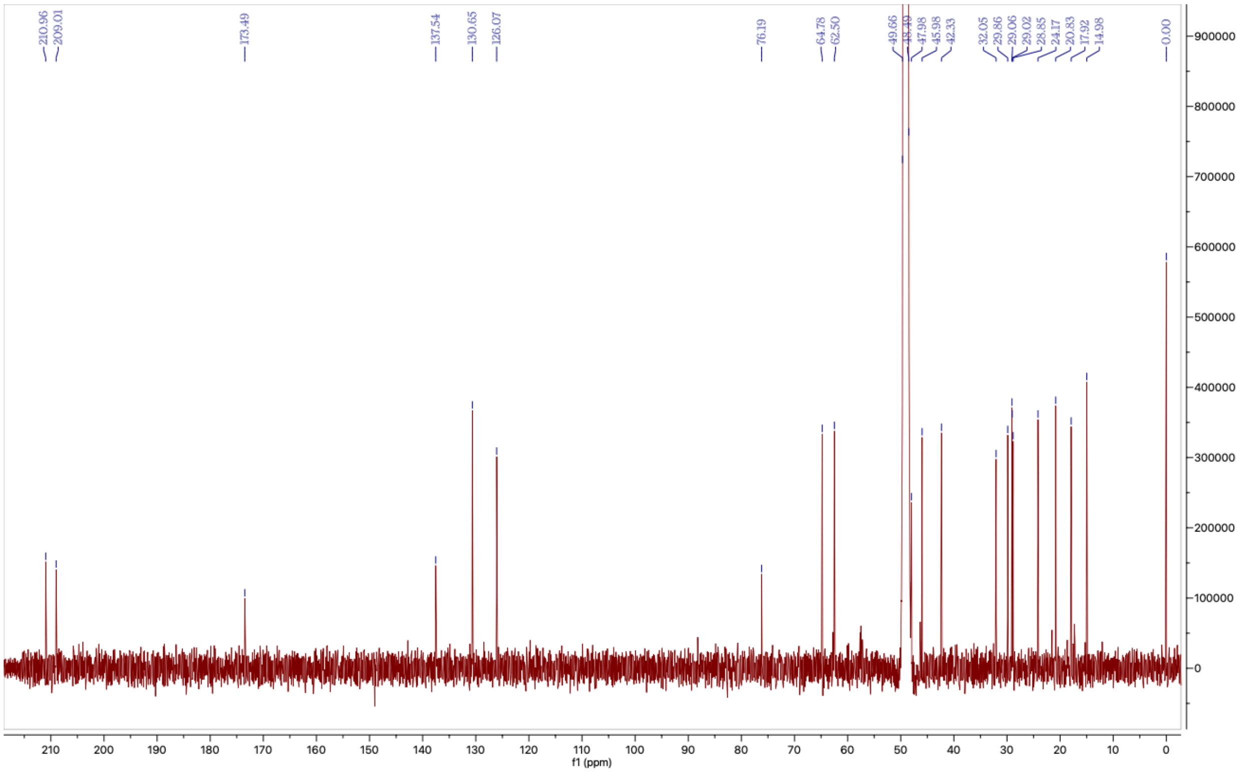

Figure S42: The ^{13}C NMR Spectrum of **13** in MeOD (150MHz)

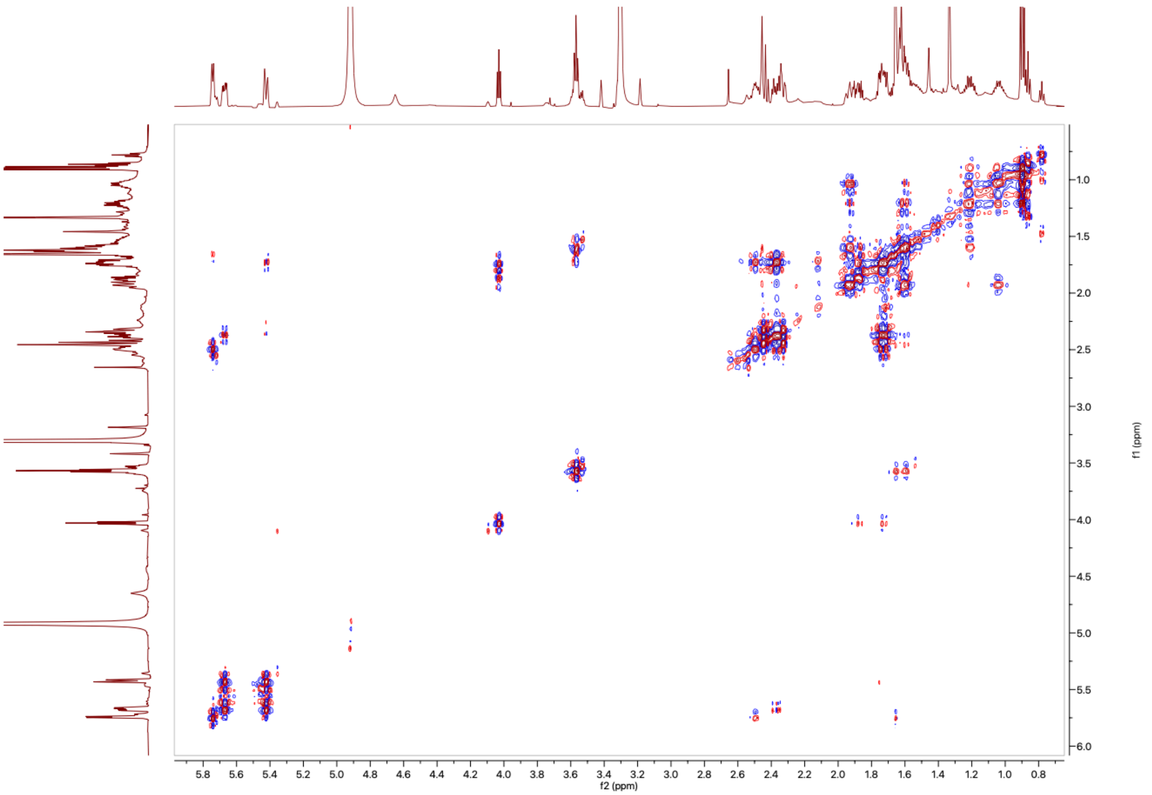

Figure S43: The ^{1}H-^{1}H COSY NMR Spectrum of **13** in MeOD (600 MHz)

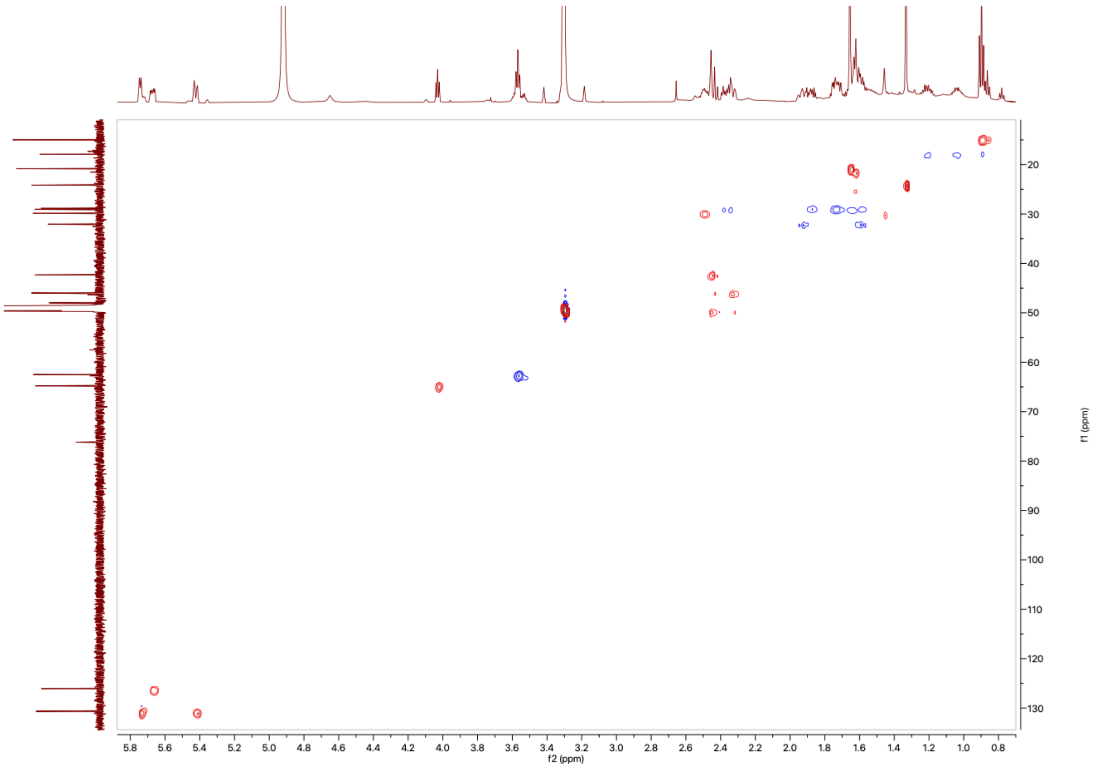

Figure S44: The ^{1}H-^{13}C HSQC NMR Spectrum of **13** in MeOD (600 MHz)

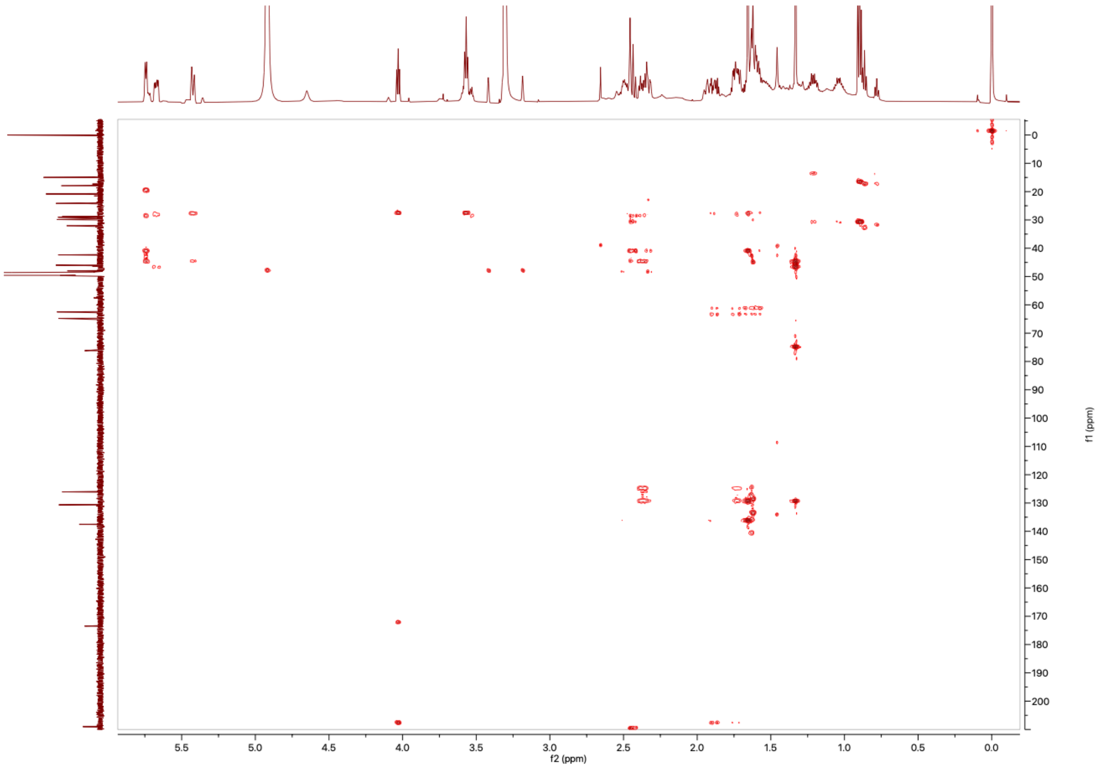

Figure S45: The ^{1}H-^{13}C HMBC NMR Spectrum of **13** in MeOD (600 MHz)

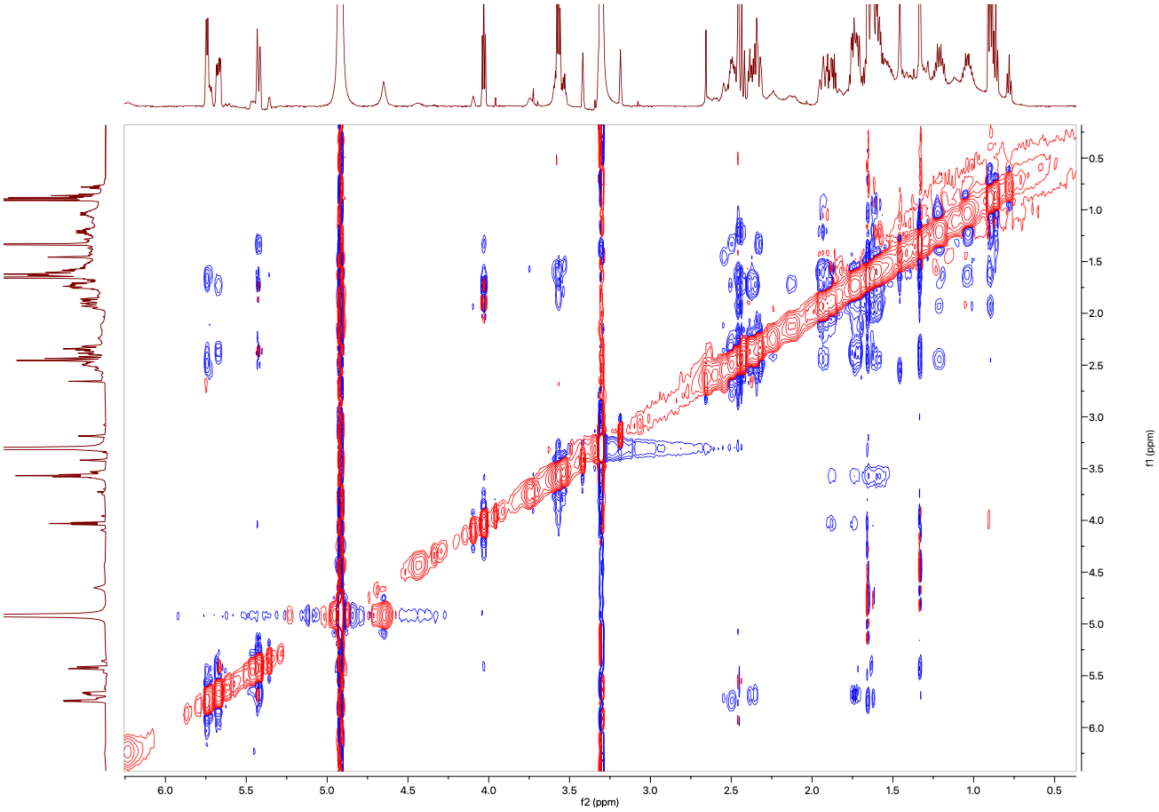

Figure S46: The ^{1}H-^{1}H NOESY NMR Spectrum of **13** in MeOD (600 MHz)

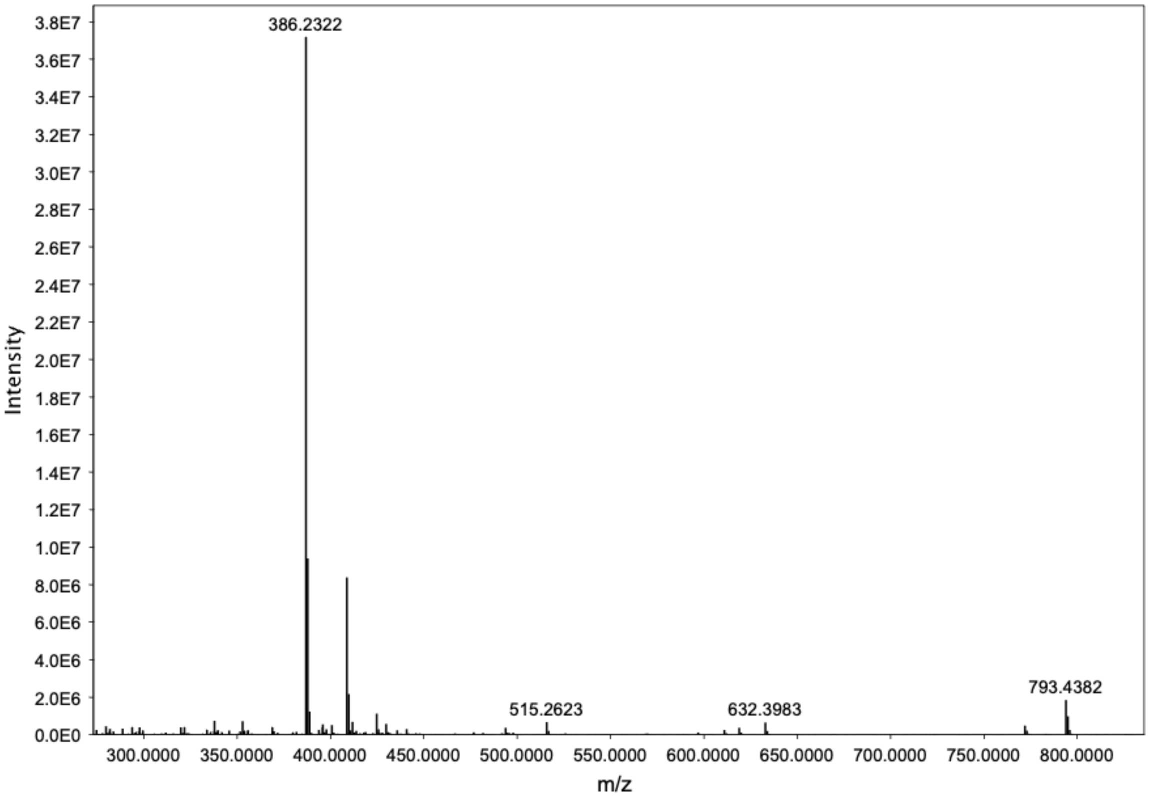

Figure S47: HRMS Spectrum of **13**

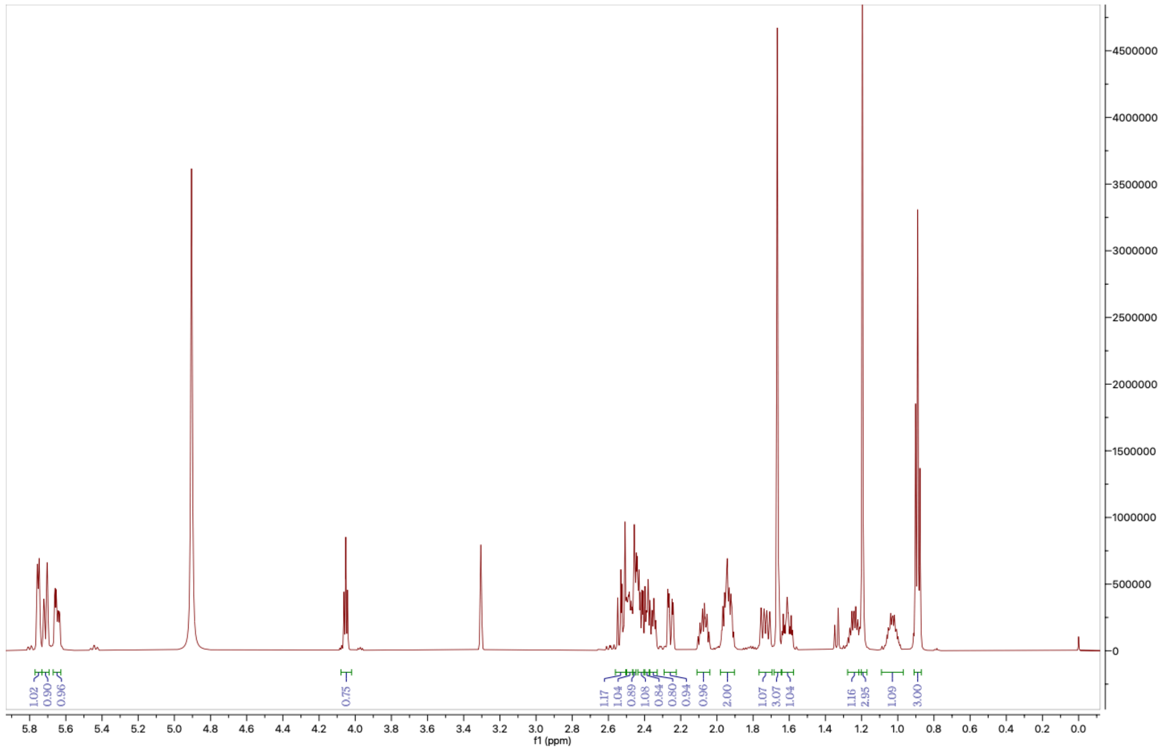

Figure S48: The ^{1}H NMR Spectrum of **14** in MeOD (600MHz)

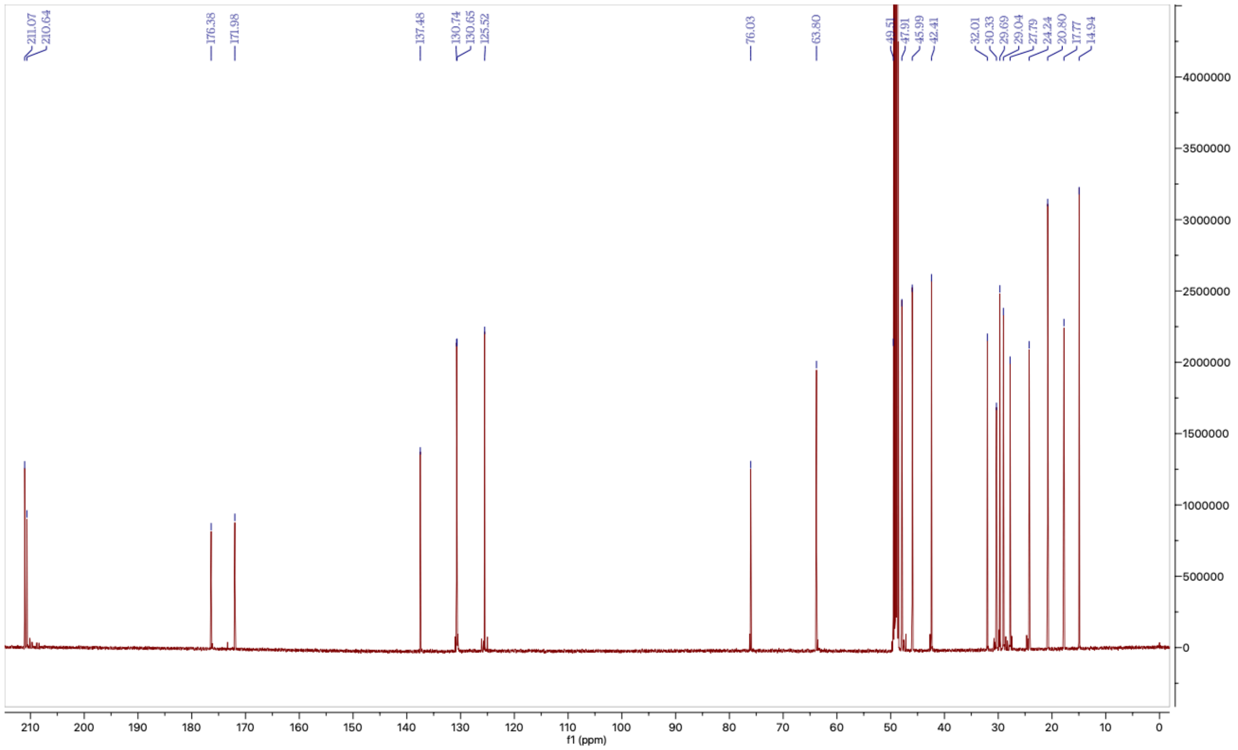

Figure S49: The ¹³C NMR Spectrum of **14** in MeOD (600MHz)

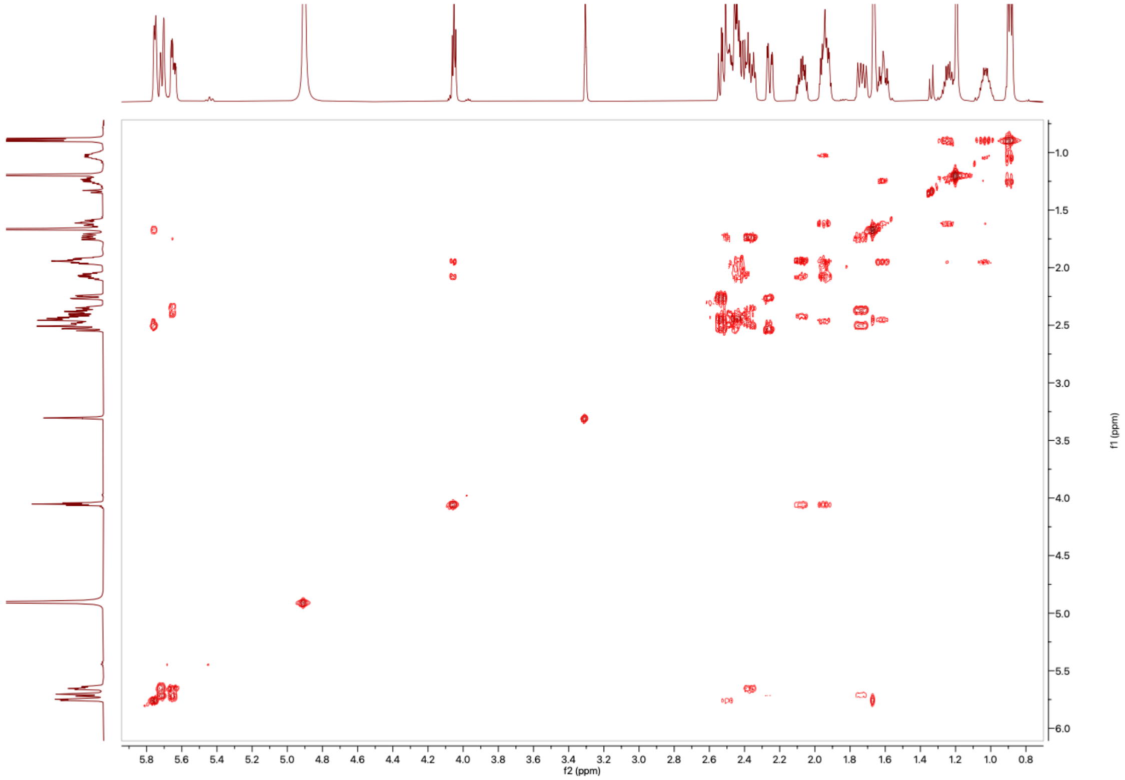

Figure S50: The ¹H-¹H COSY NMR Spectrum of **14** in MeOD (600 MHz)

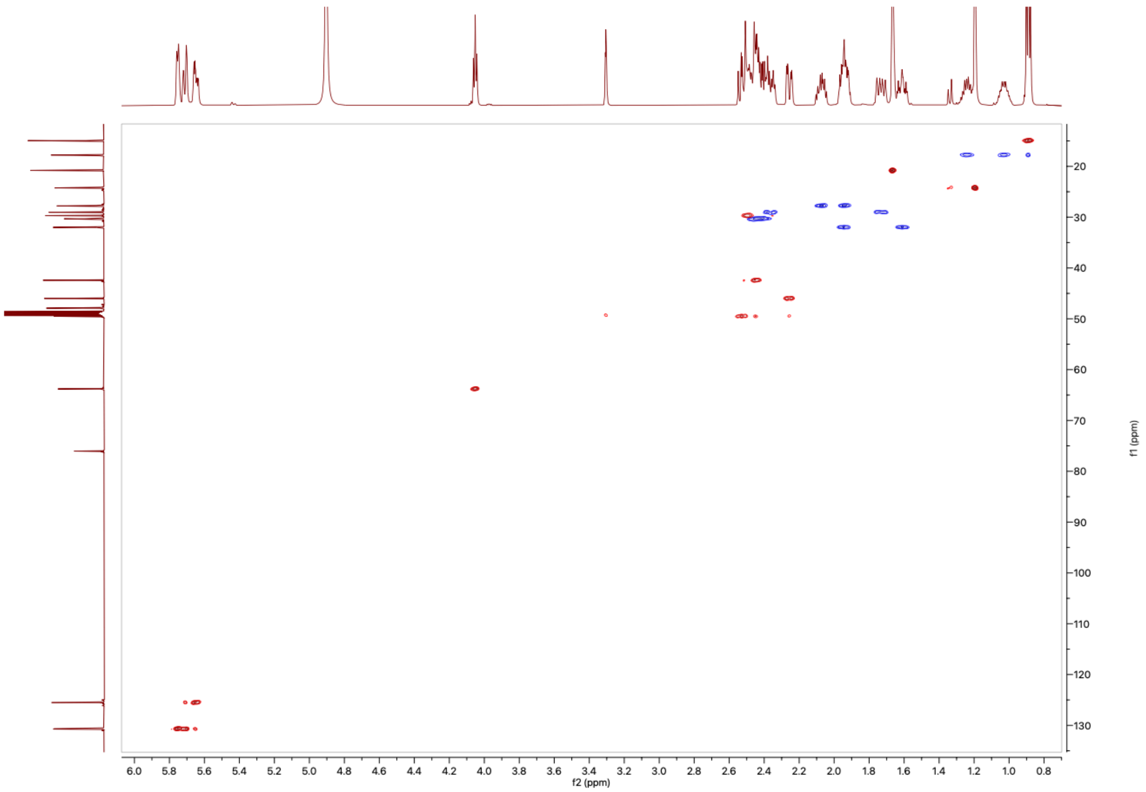

Figure S51: The ^{1}H-^{13}C HSQC NMR Spectrum of **14** in MeOD (600 MHz)

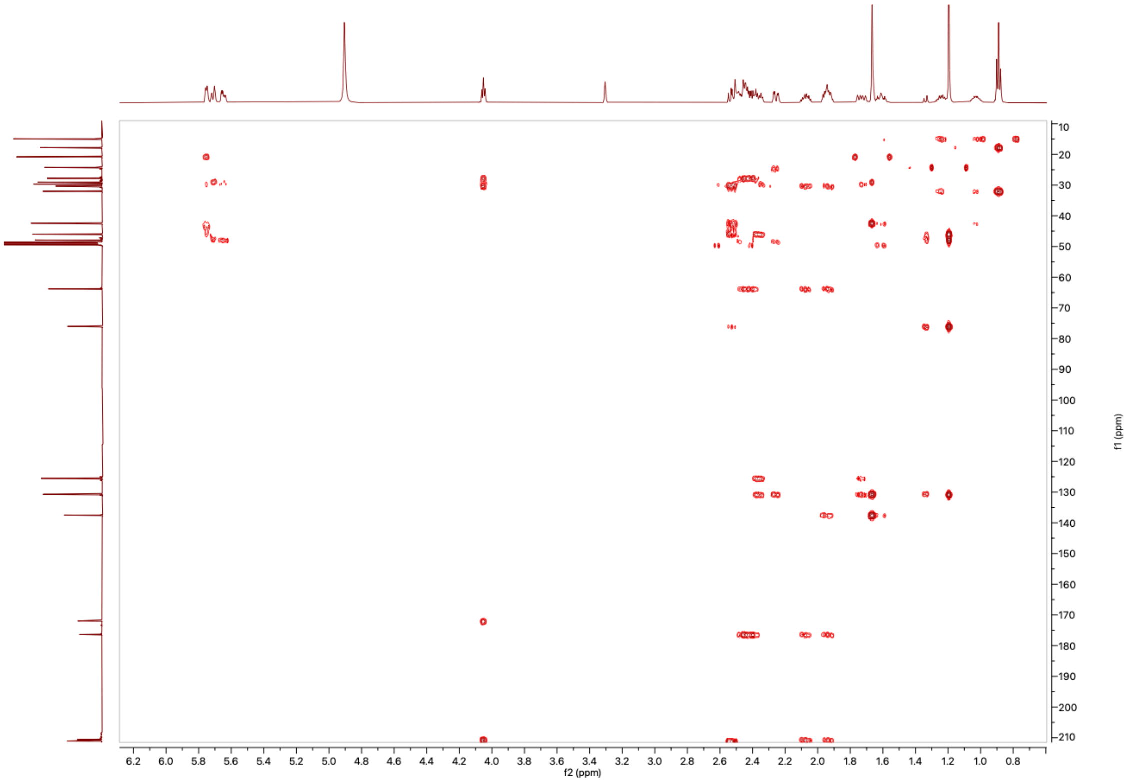

Figure S52: The ^{1}H-^{13}C HMBC NMR Spectrum of **14** in MeOD (600 MHz)

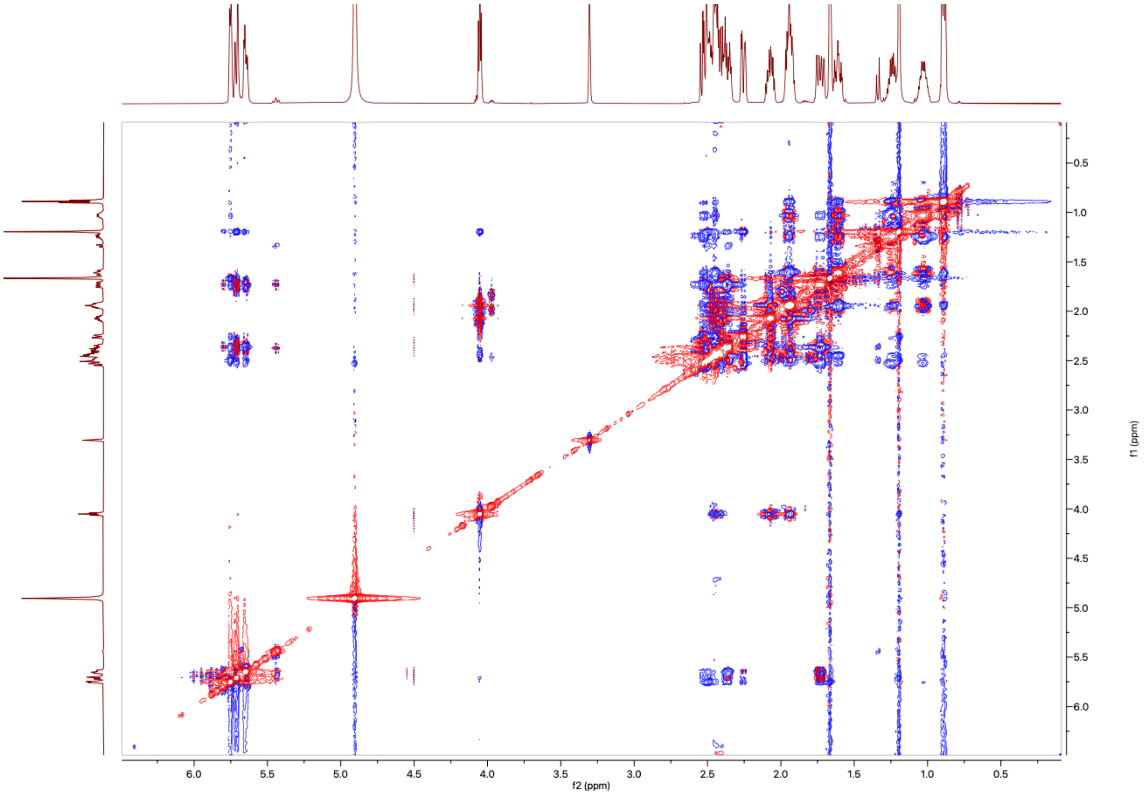

Figure S53: The ^1H-^1H NOESY NMR Spectrum of **14** in MeOD (600 MHz)

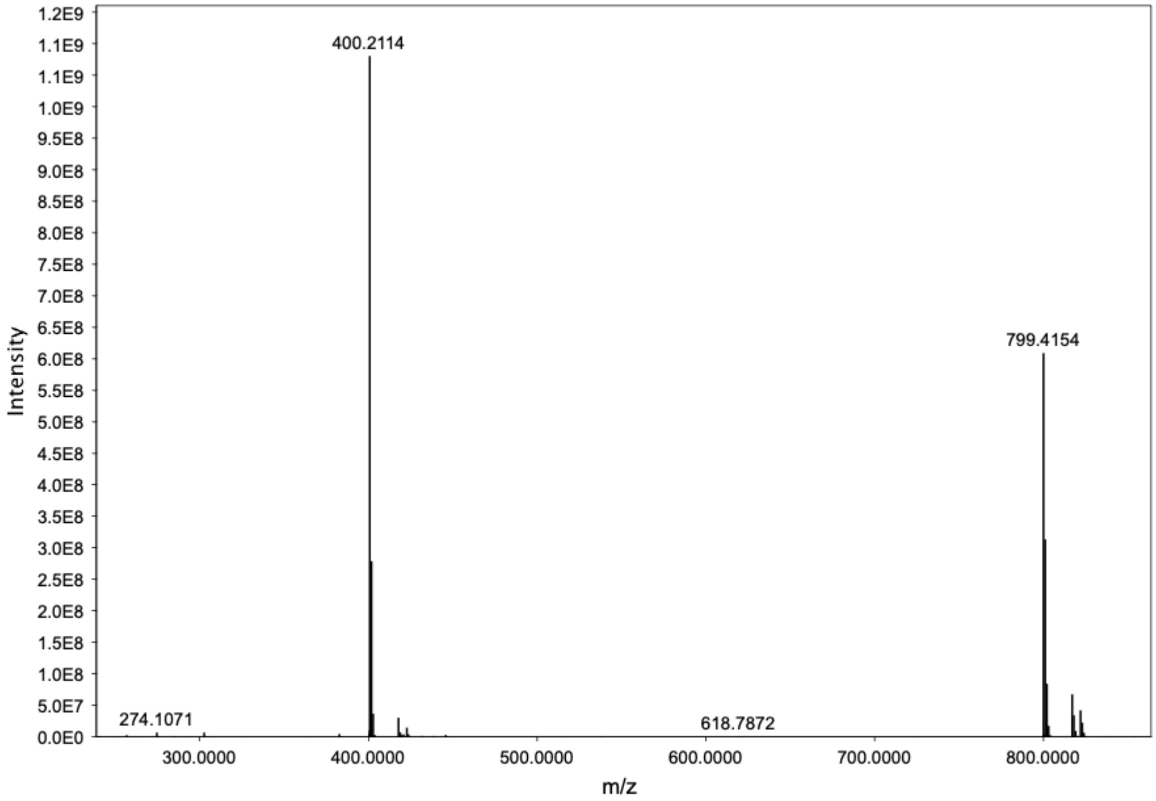

Figure S54: HRMS spectrum of **14**

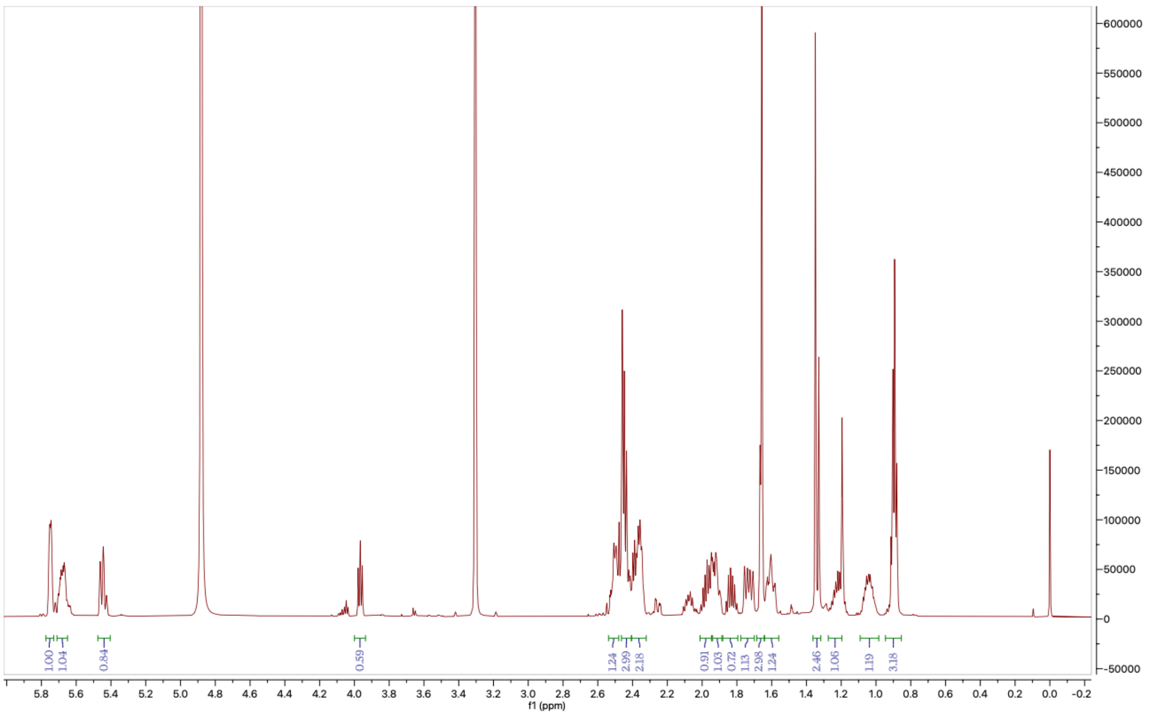

Figure S55: The ^{1}H NMR Spectrum of **15** in MeOD (600MHz)

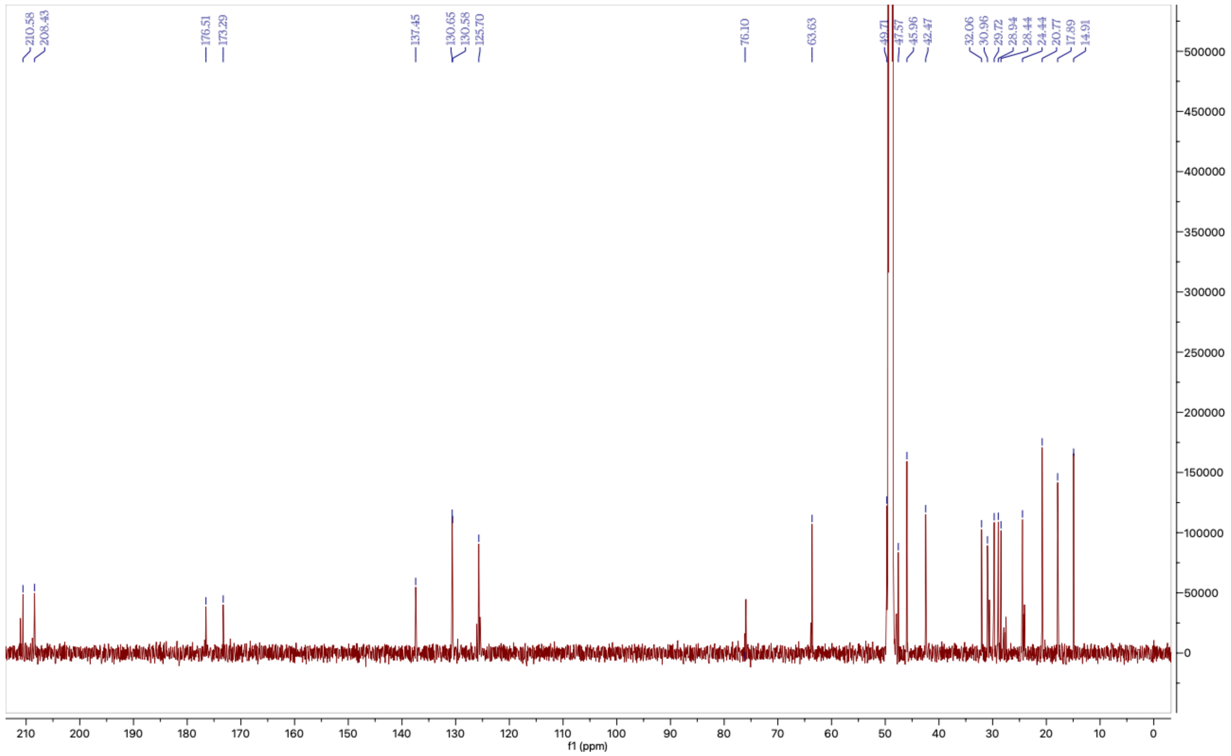

Figure S56: The ^{13}C NMR Spectrum of **15** in MeOD (600MHz)

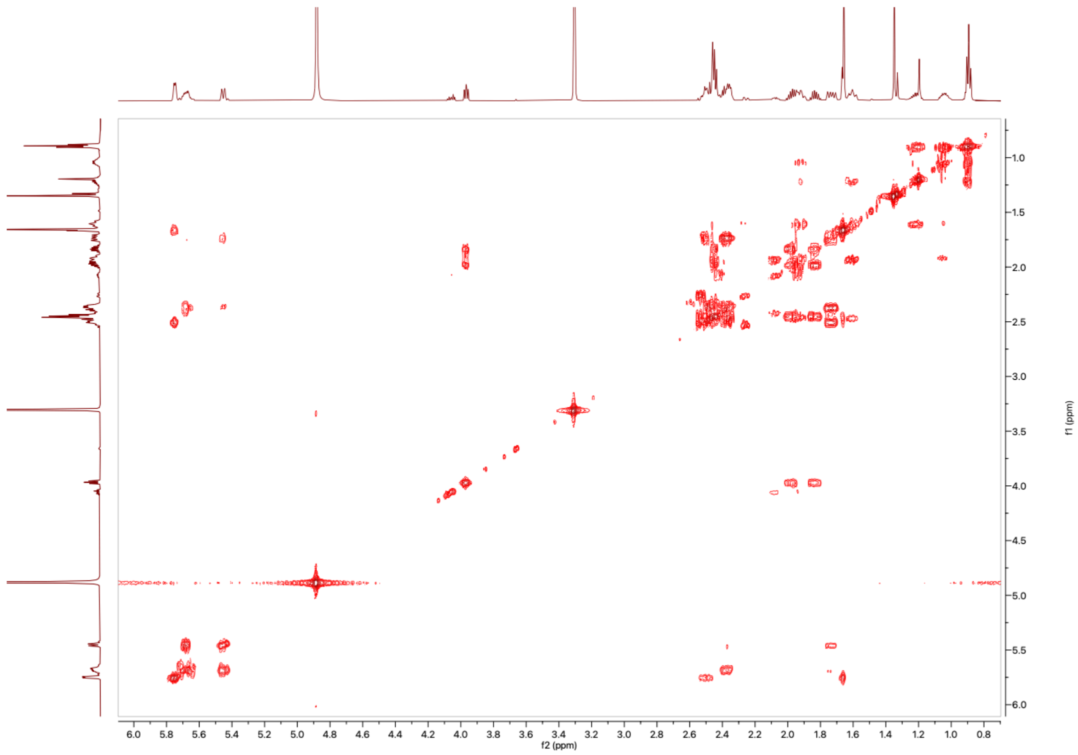

Figure S57: The ^{1}H-^{1}H COSY NMR Spectrum of **15** in MeOD (600 MHz)

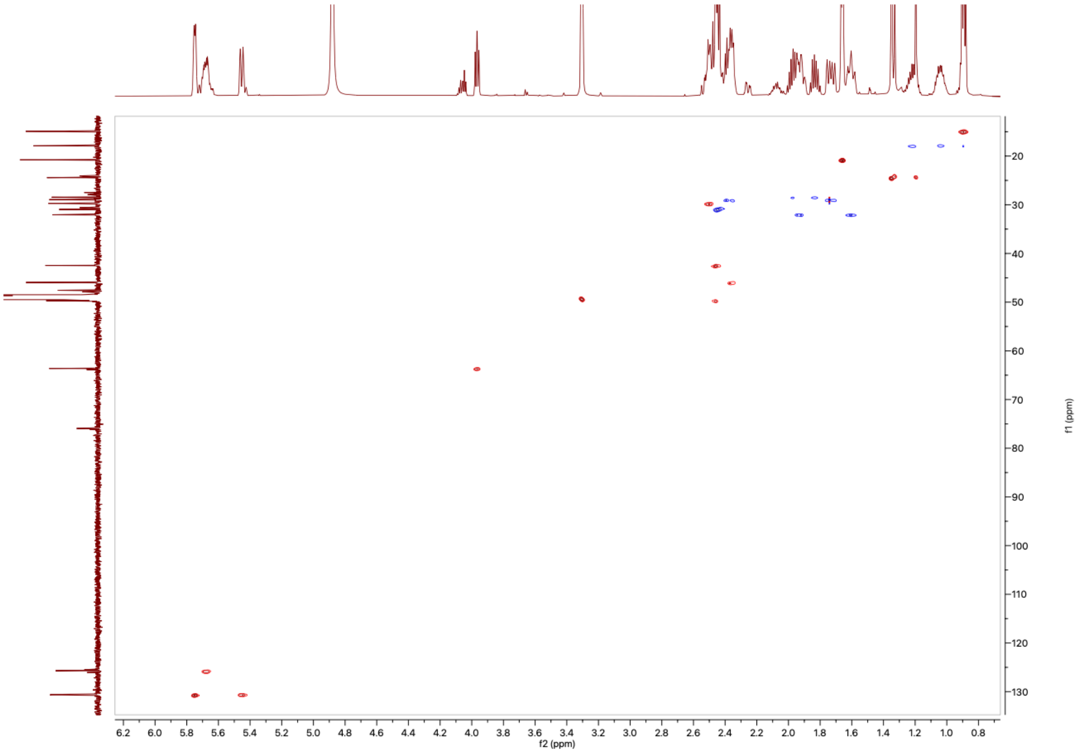

Figure S58: The ^{1}H-^{13}C HSQC NMR Spectrum of **15** in MeOD (600 MHz)

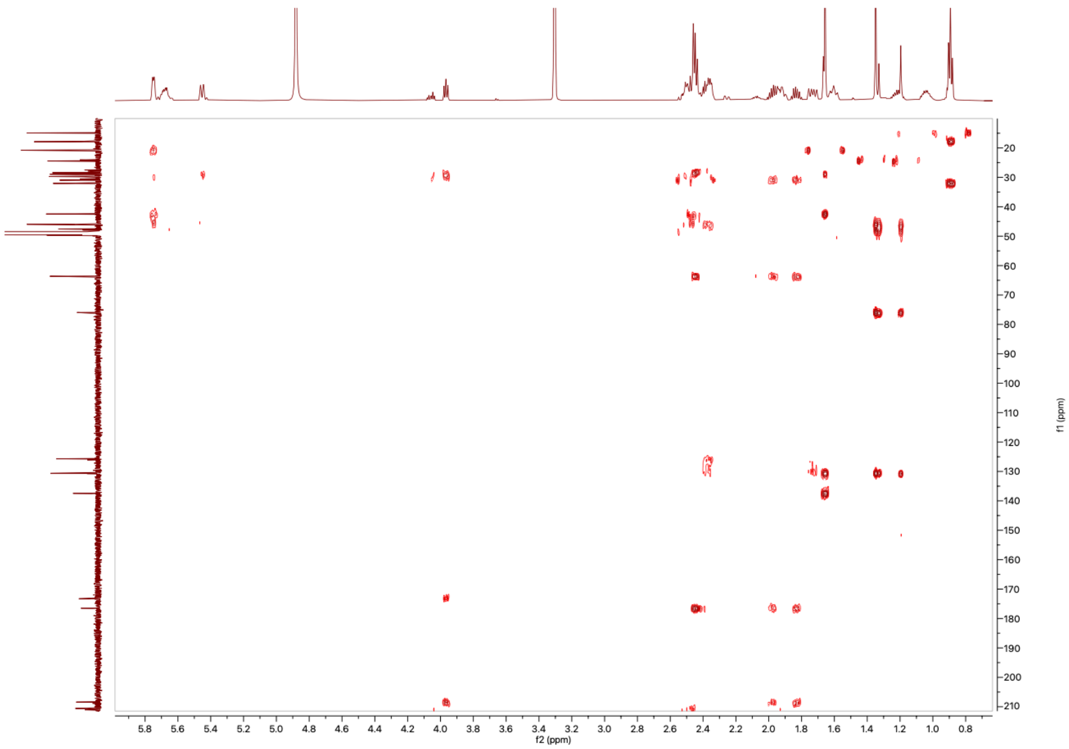

Figure S59: The ^{1}H-^{13}C HMBC NMR Spectrum of **15** in MeOD (600 MHz)

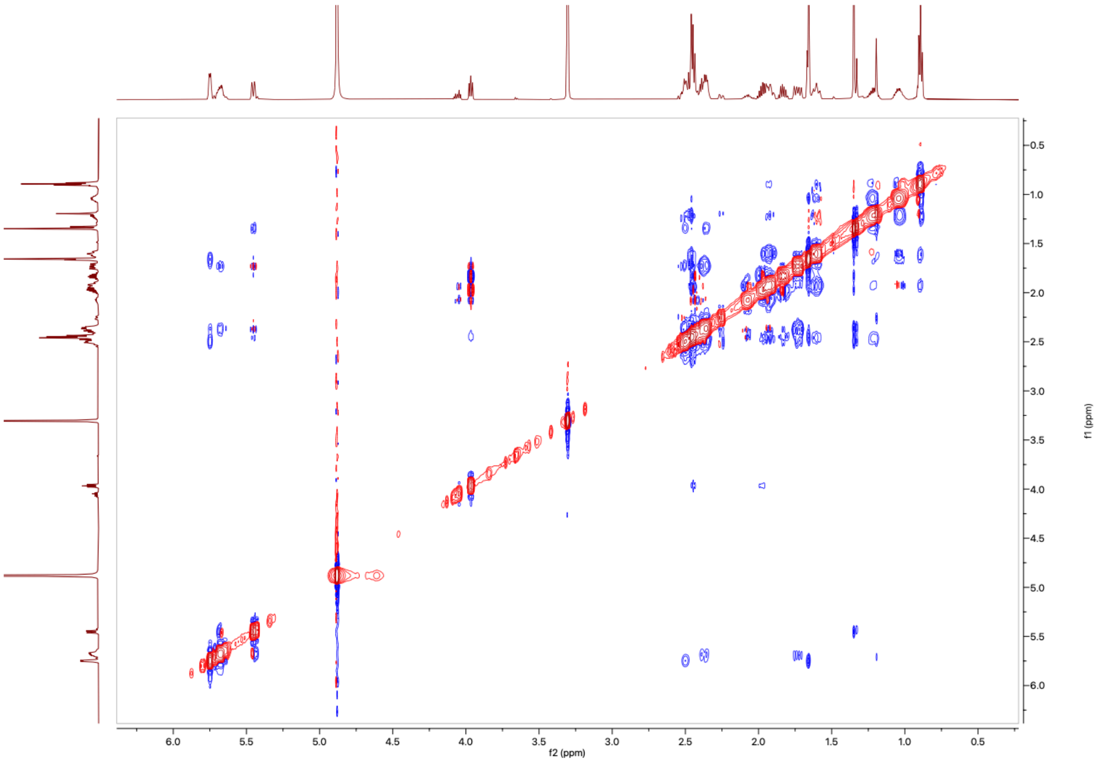

Figure S60: The ^{1}H-^{1}H NOESY NMR Spectrum of **15** in MeOD (600 MHz)

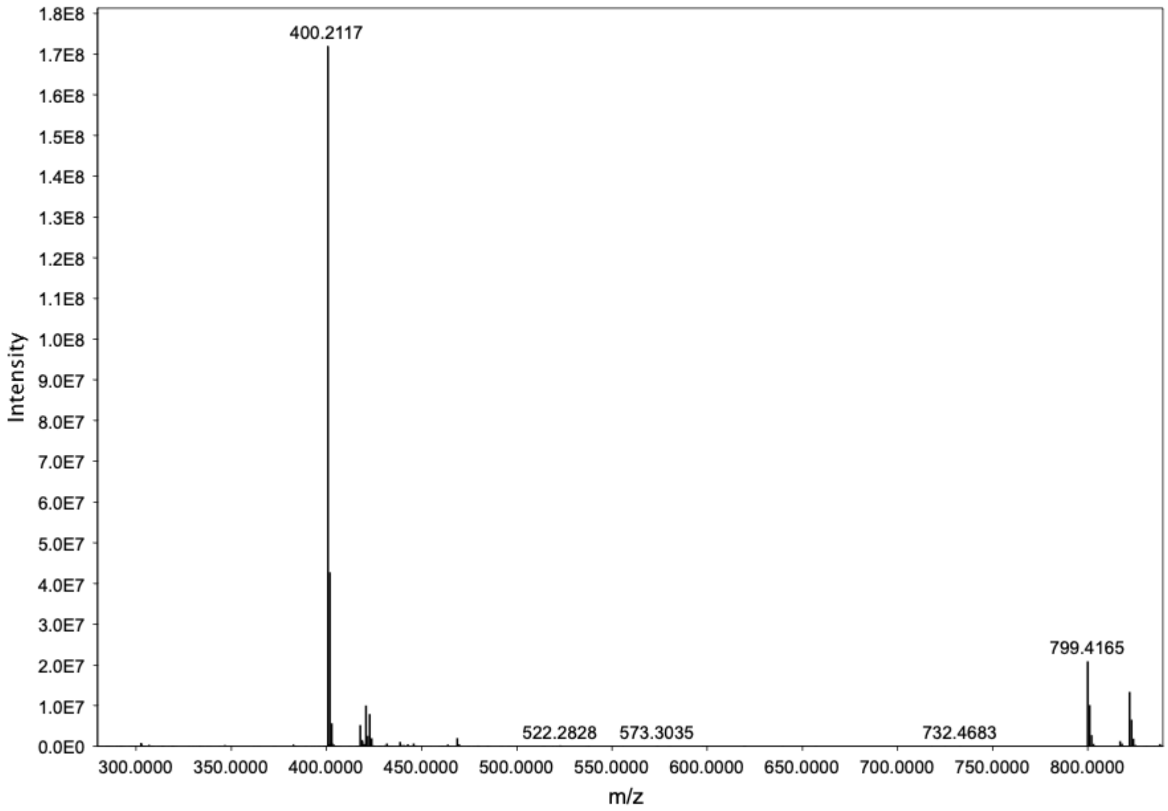

Figure S61: HRMS spectrum of **15**

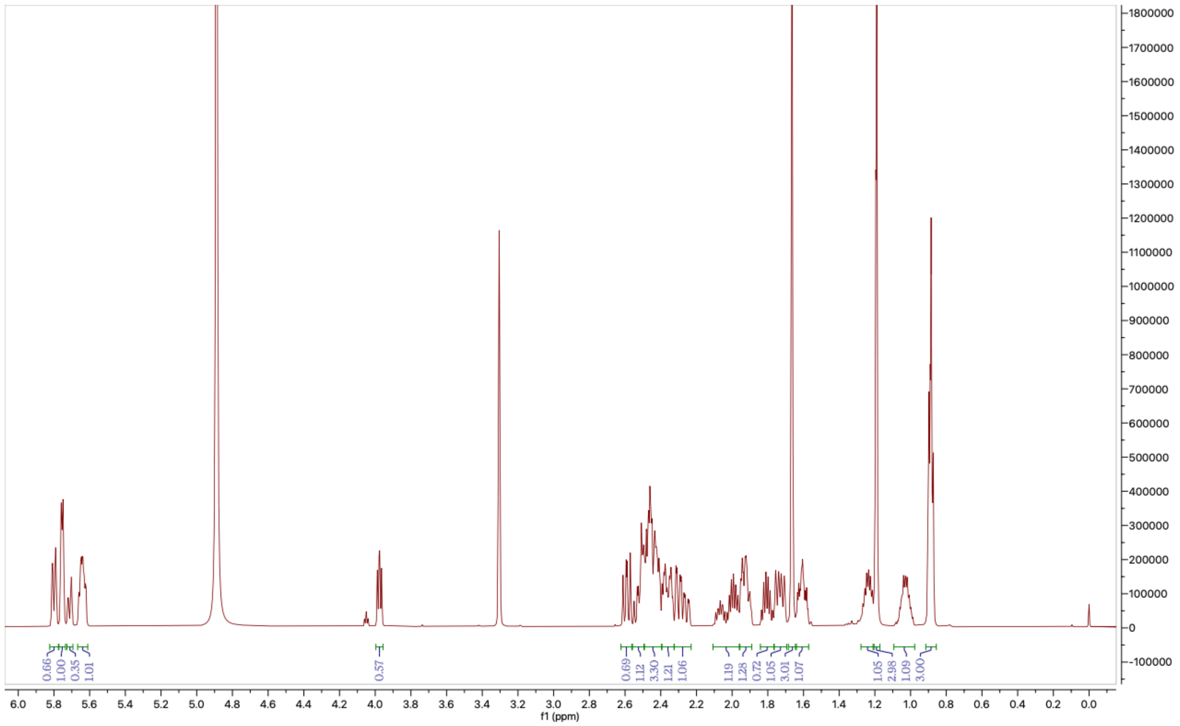

Figure S62: The ¹H NMR Spectrum of **16** in MeOD (600MHz)

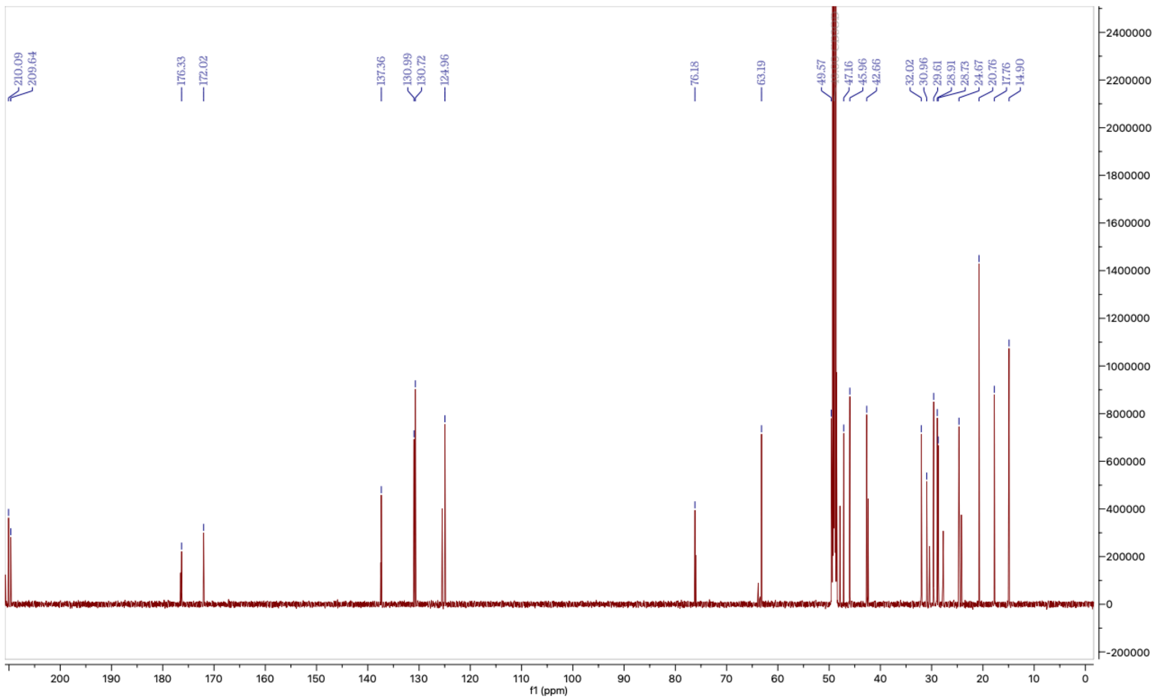

Figure S63: The ^{13}C NMR Spectrum of **16** in MeOD (600MHz)

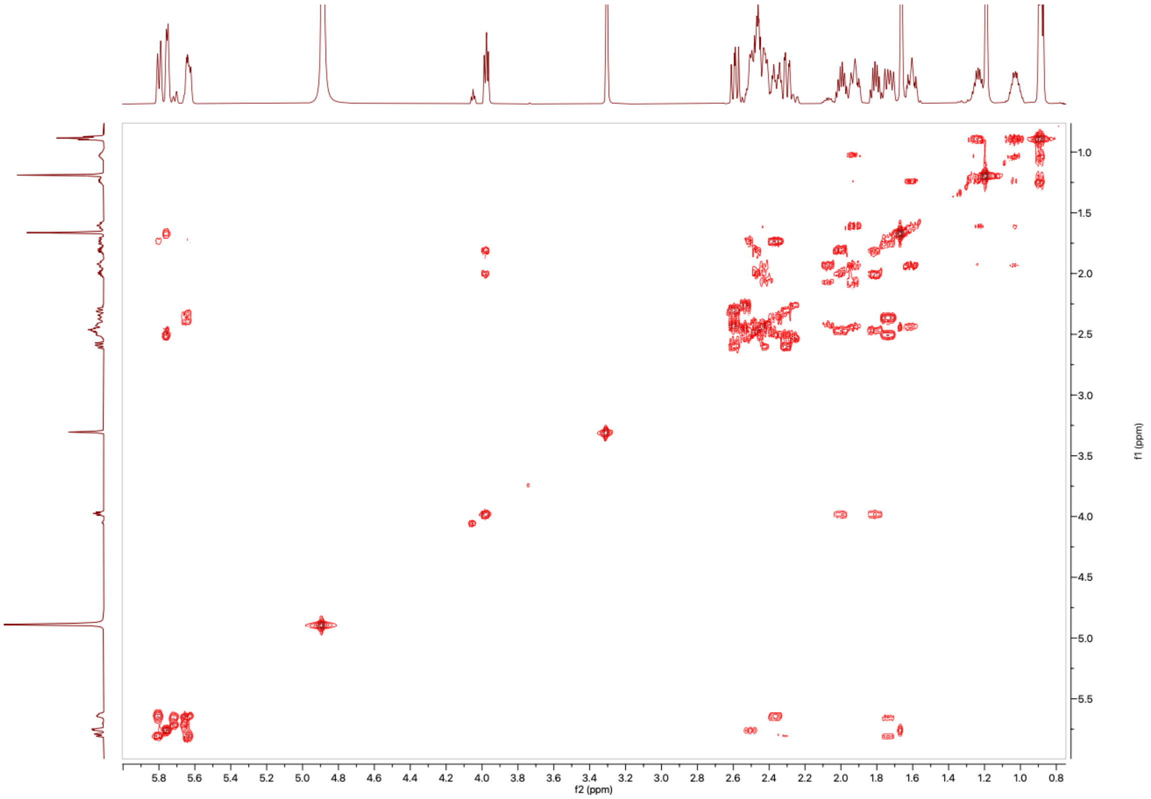

Figure S64: The ^{1}H-^{1}H COSY NMR Spectrum of **16** in MeOD (600 MHz)

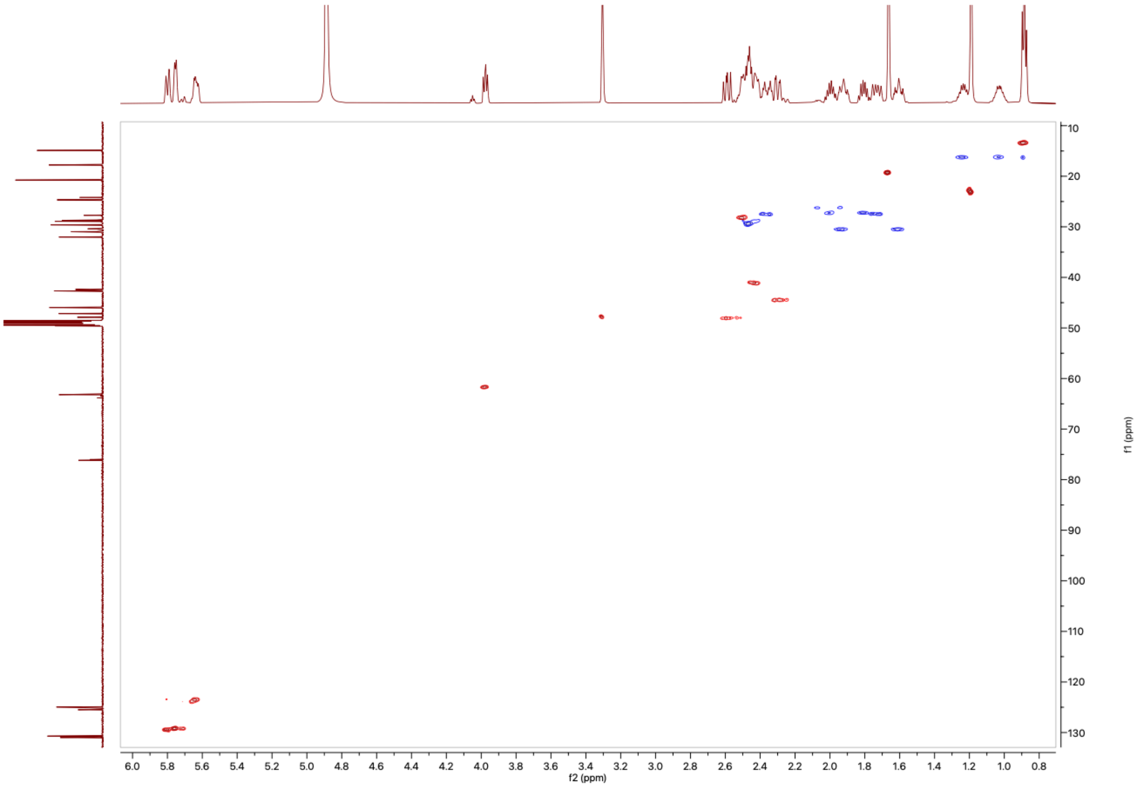

Figure S65: The ¹H-¹³C HSQC NMR Spectrum of **16** in MeOD (600 MHz)

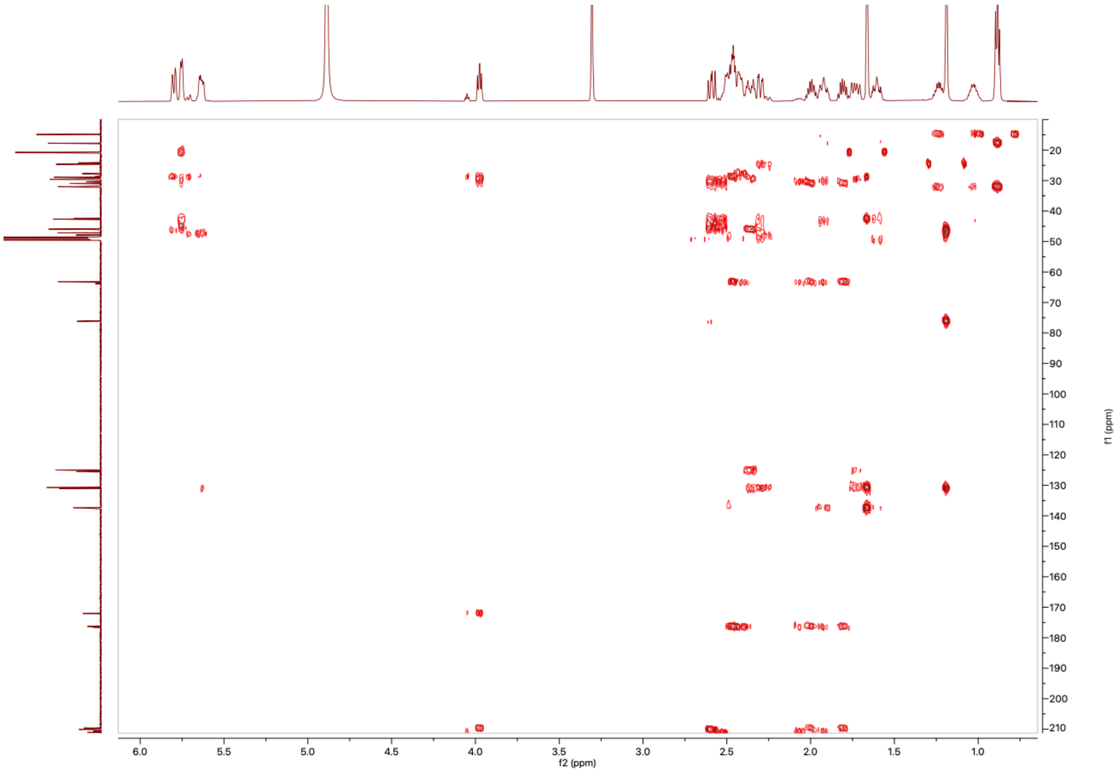

Figure S66: The ¹H-¹³C HMBC NMR Spectrum of **16** in MeOD (600 MHz)

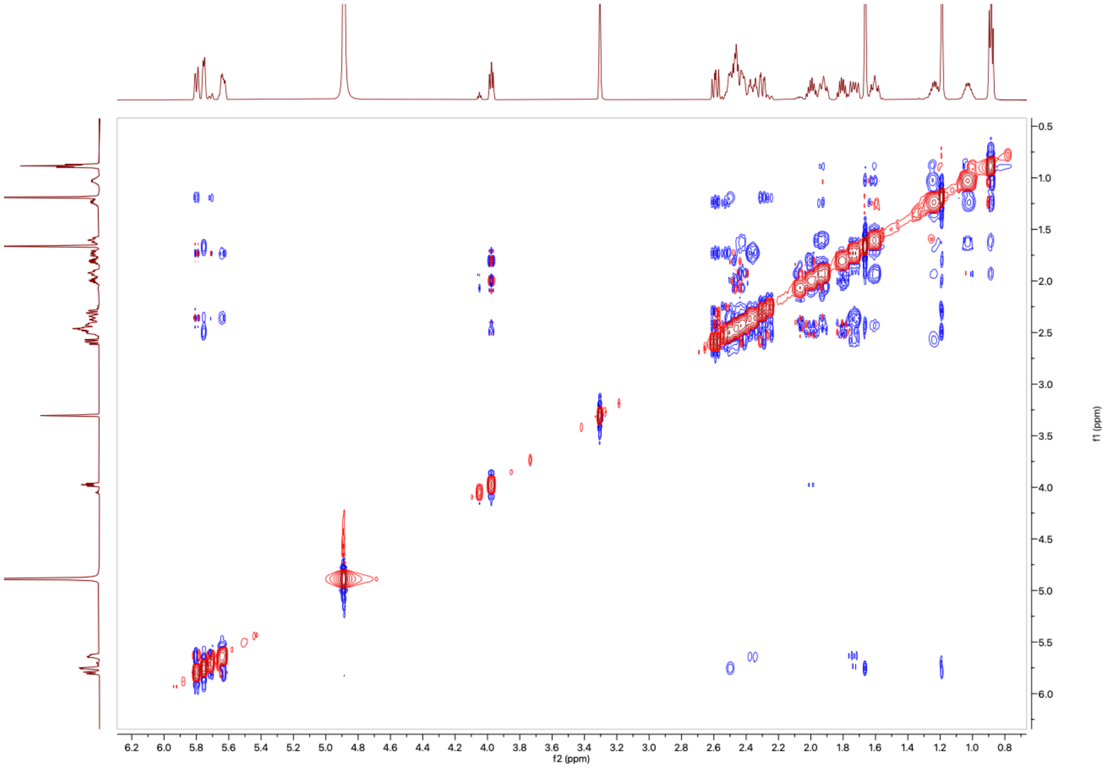

Figure S67: The ¹H-¹H NOESY NMR Spectrum of **16** in MeOD (600 MHz)

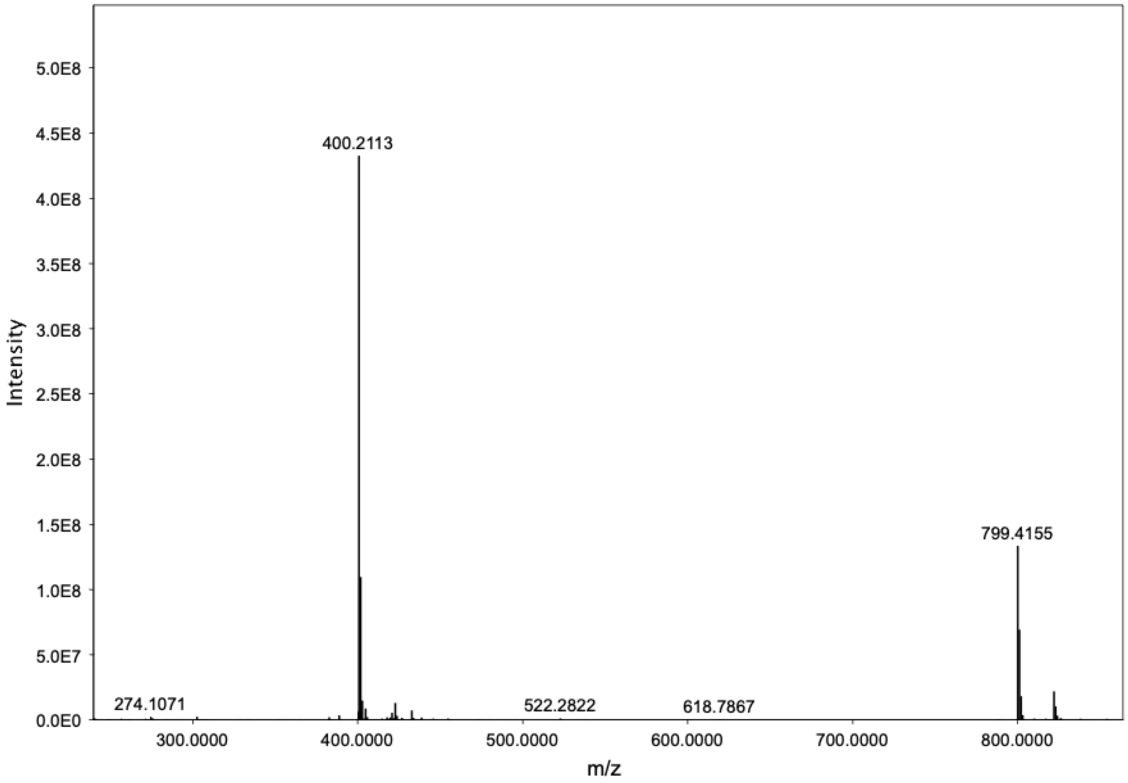

Figure S68: HRMS spectrum of **16**

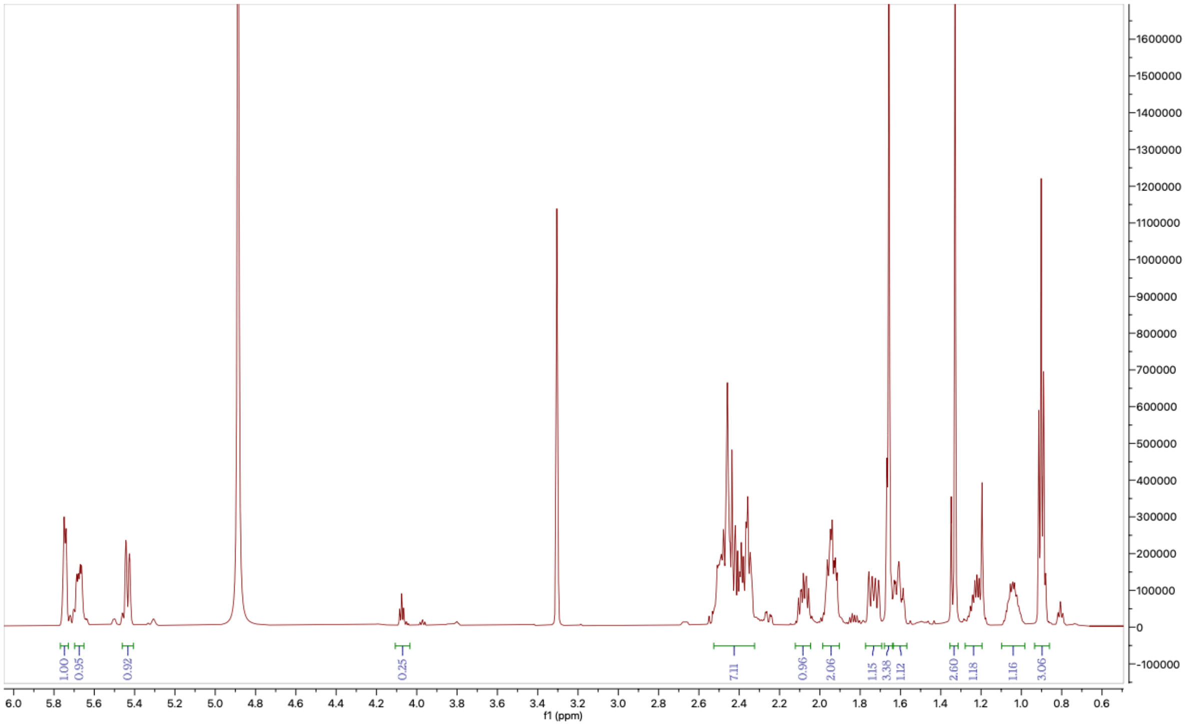

Figure S69: The ^{1}H NMR Spectrum of **17** in MeOD (600MHz)

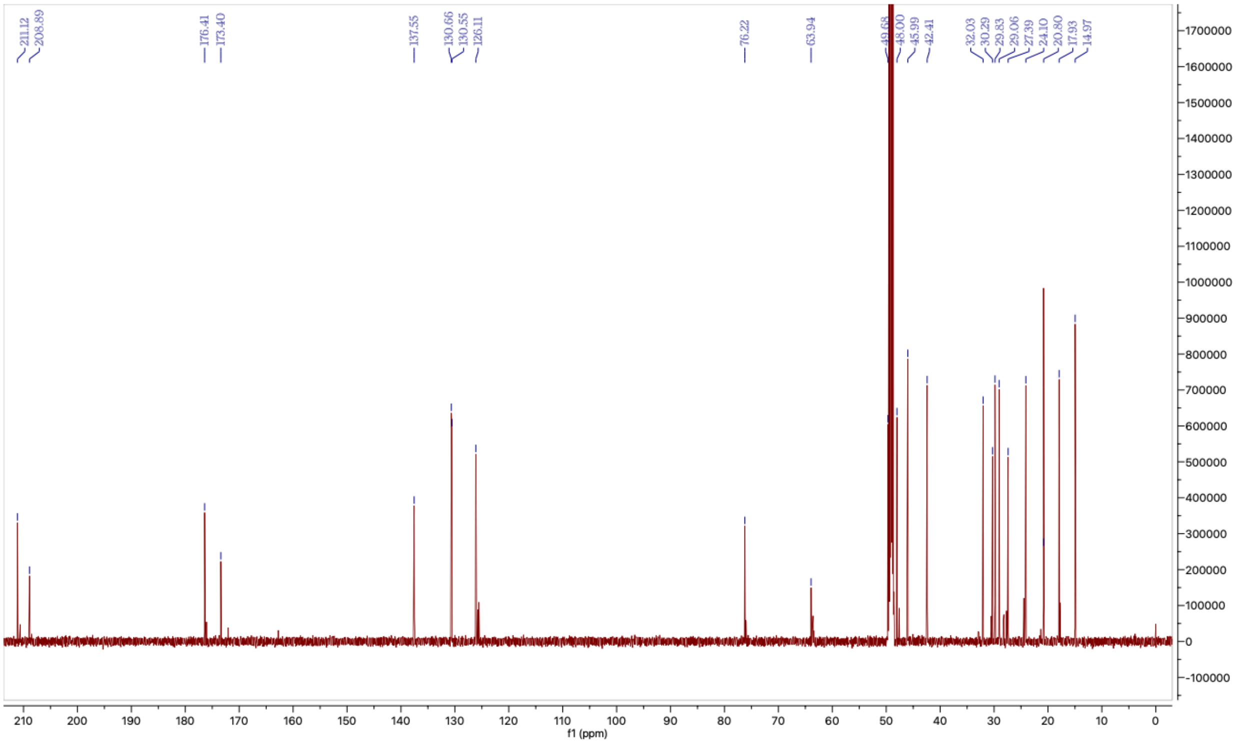

Figure S70: The ^{13}C NMR Spectrum of **17** in MeOD (600MHz)

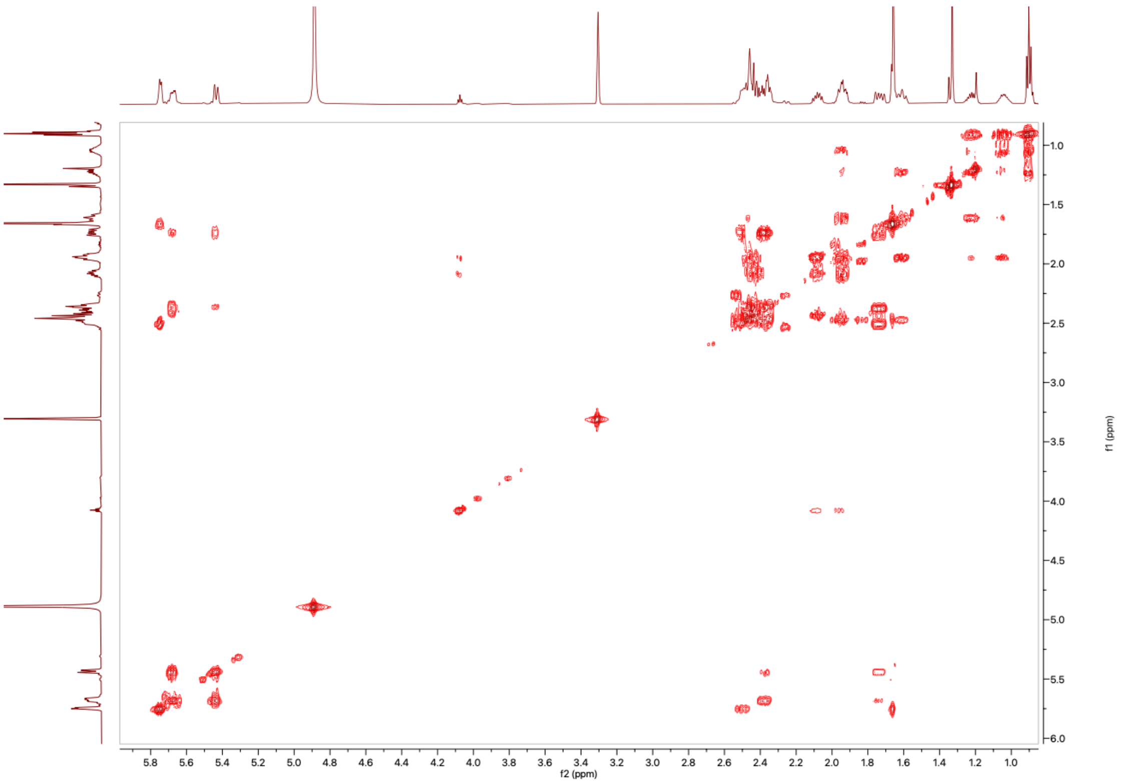

Figure S71: The ¹H-¹H COSY NMR Spectrum of **17** in MeOD (600 MHz)

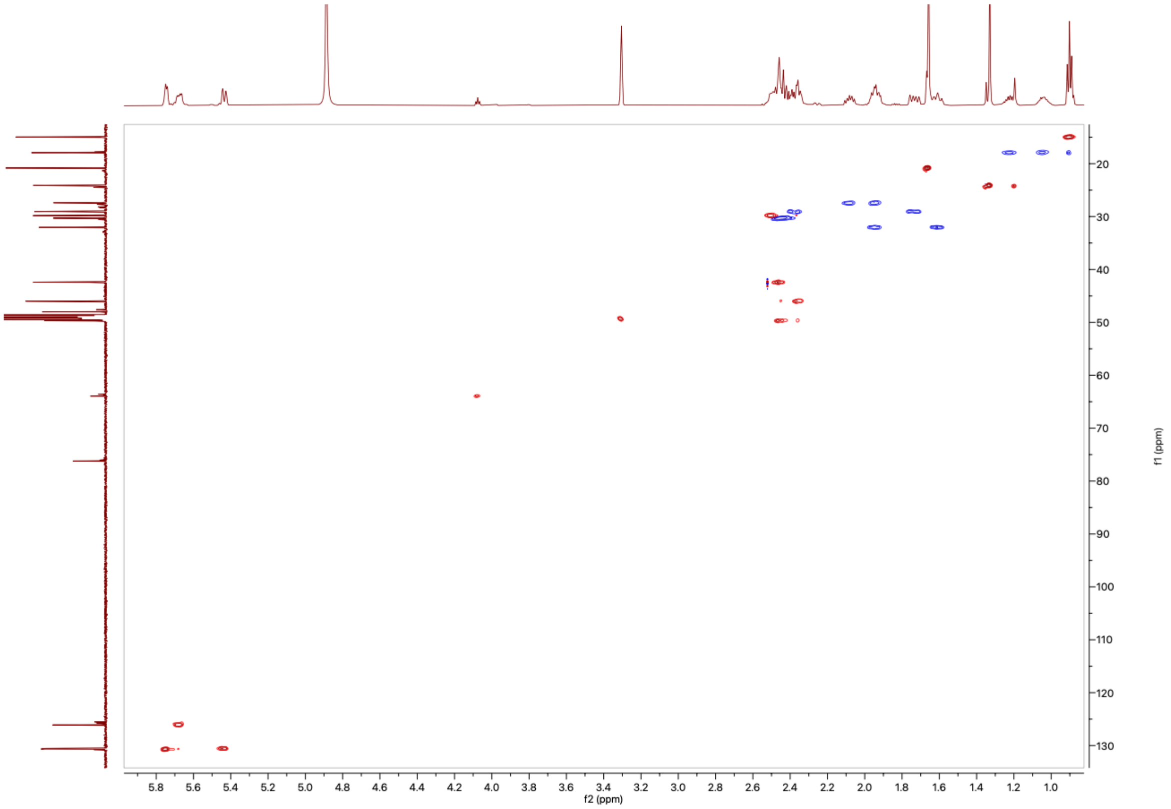

Figure S72: The ¹H-¹³C HSQC NMR Spectrum of **17** in MeOD (600 MHz)

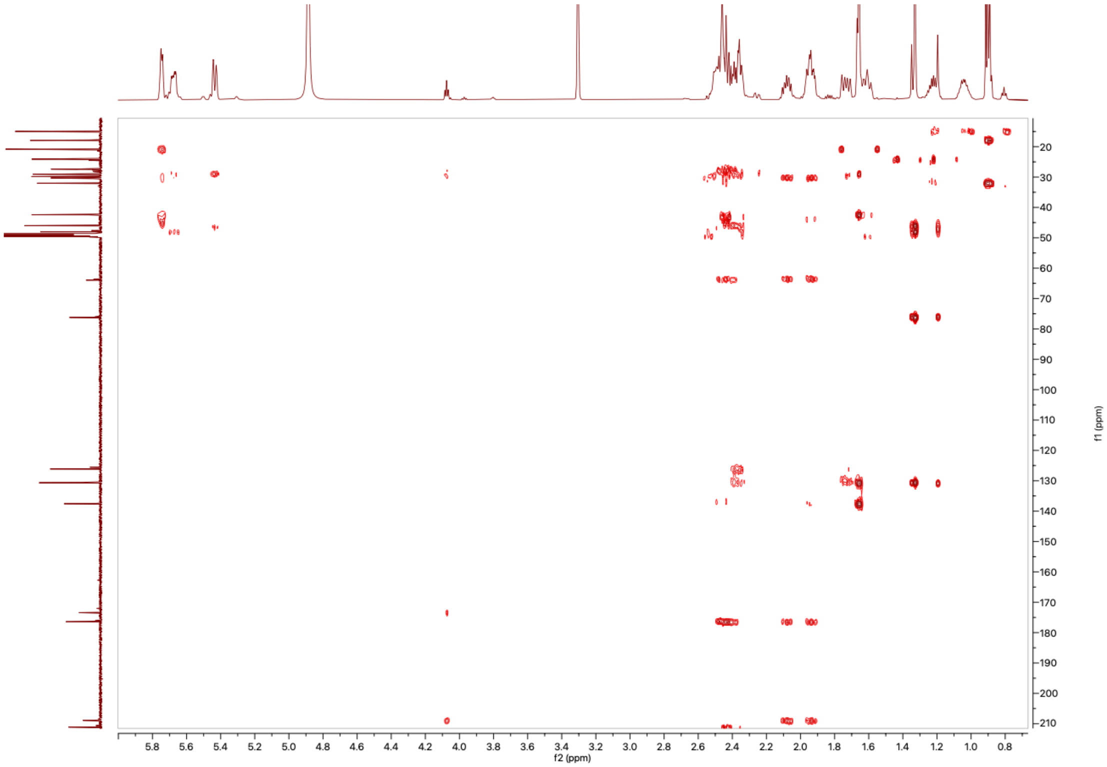

Figure S73: The ^{1}H-^{13}C HMBC NMR Spectrum of **17** in MeOD (600 MHz)

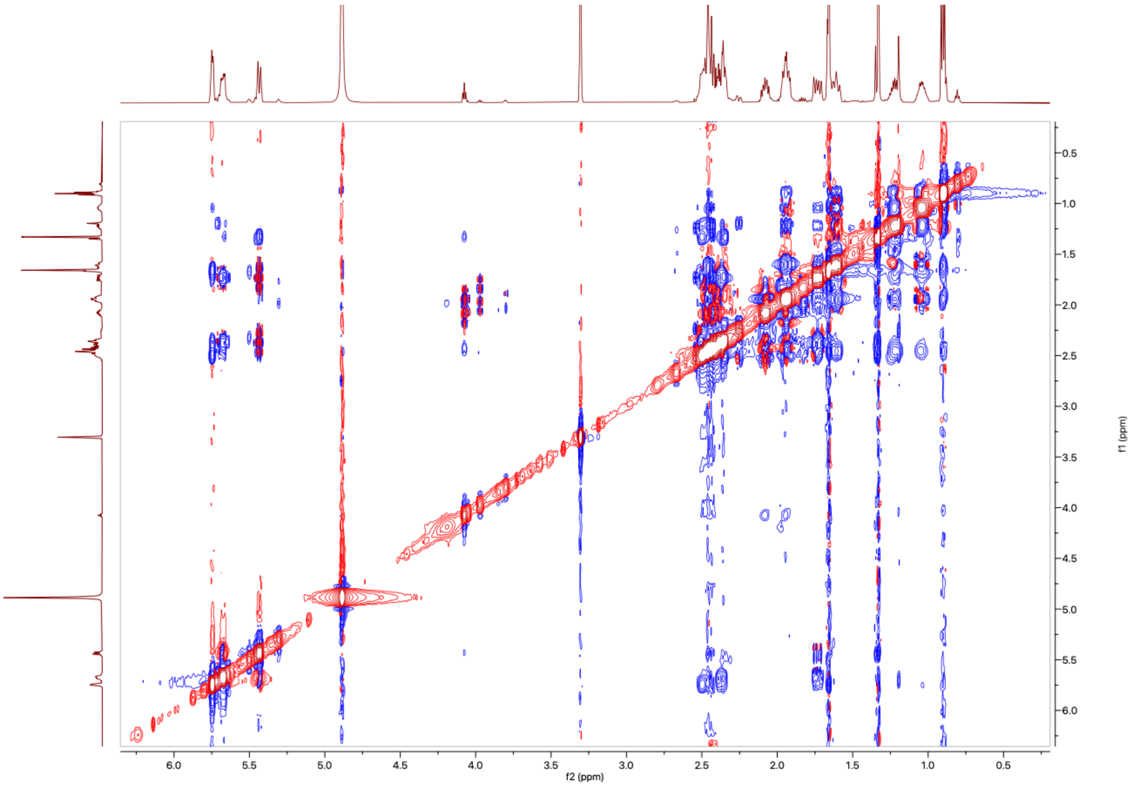

Figure S74: The ^{1}H-^{1}H NOESY NMR Spectrum of **17** in MeOD (600 MHz)

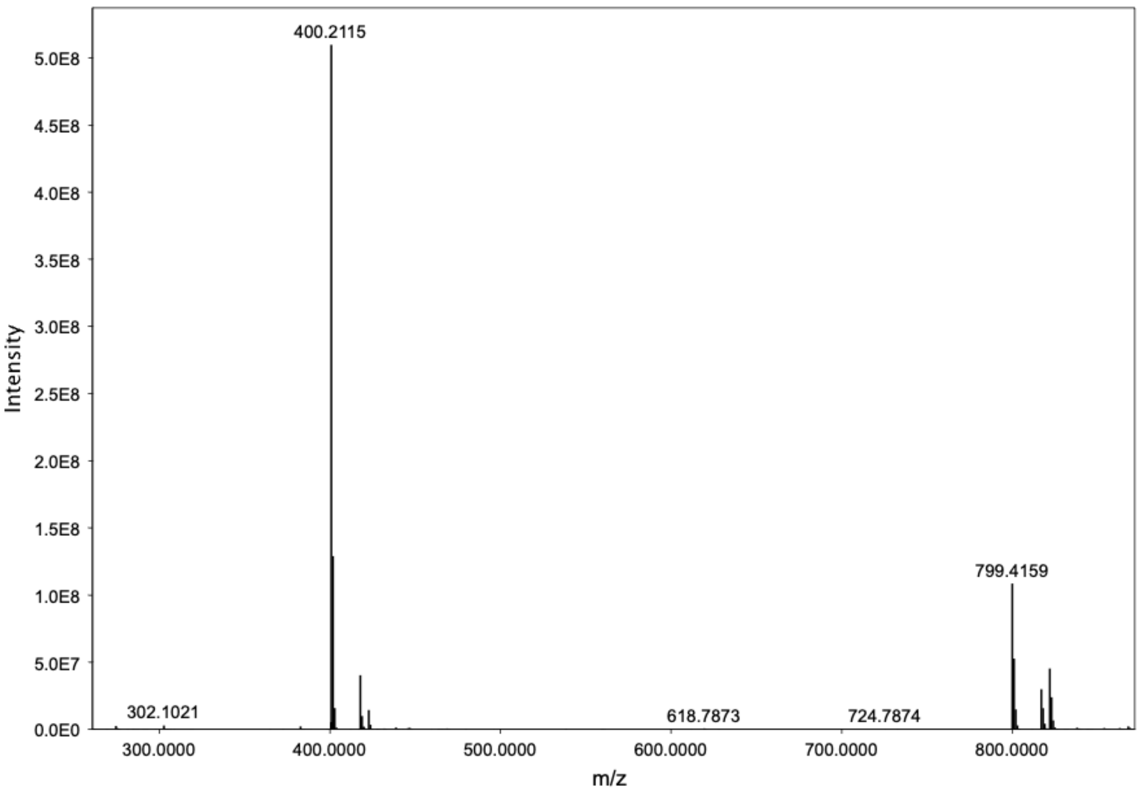

Figure S75: HRMS spectrum of **17**

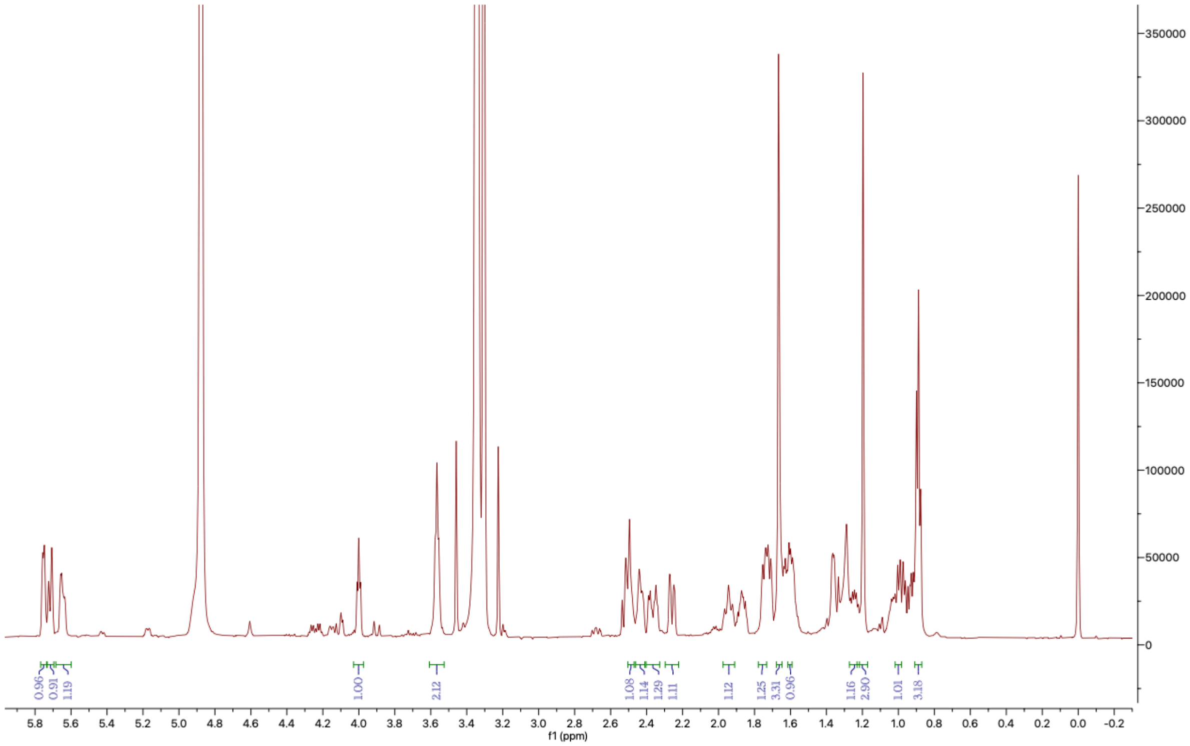

Figure S76: The ¹H NMR Spectrum of **19** in MeOD (600MHz)

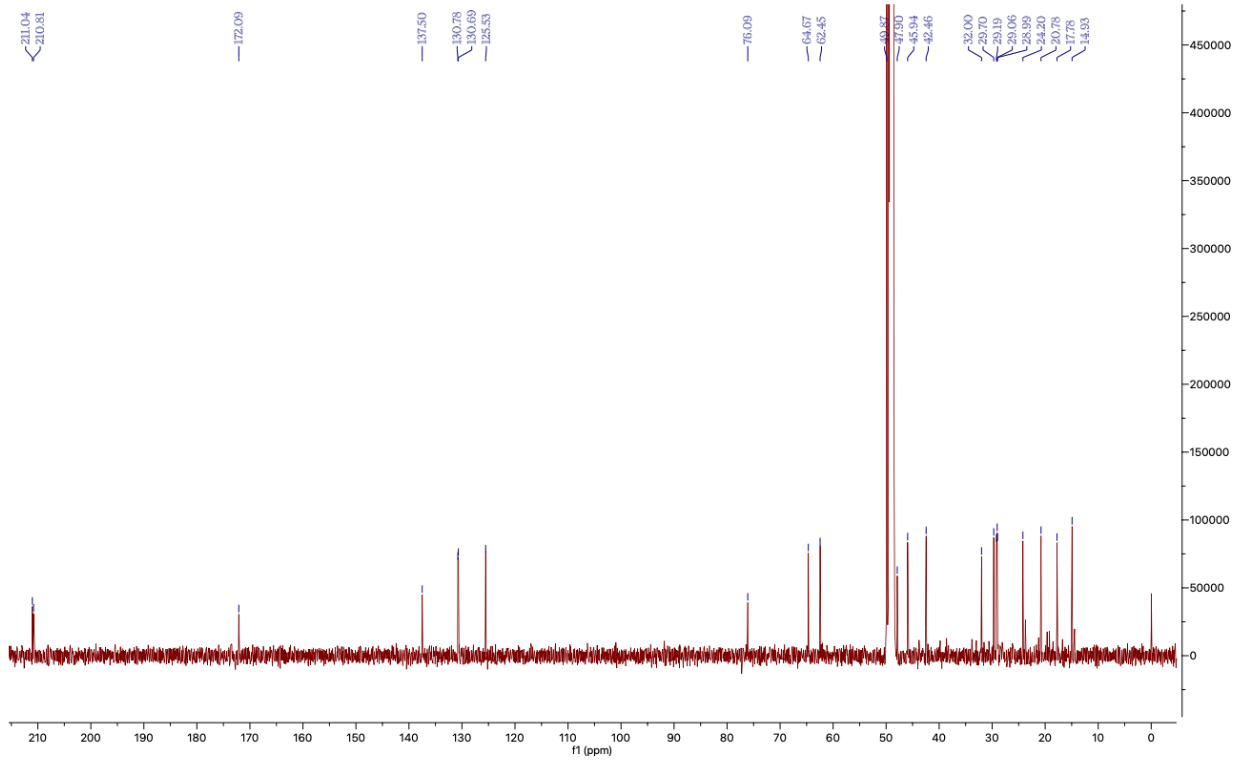

Figure S77: The ^{13}C NMR Spectrum of **19** in MeOD (150MHz)

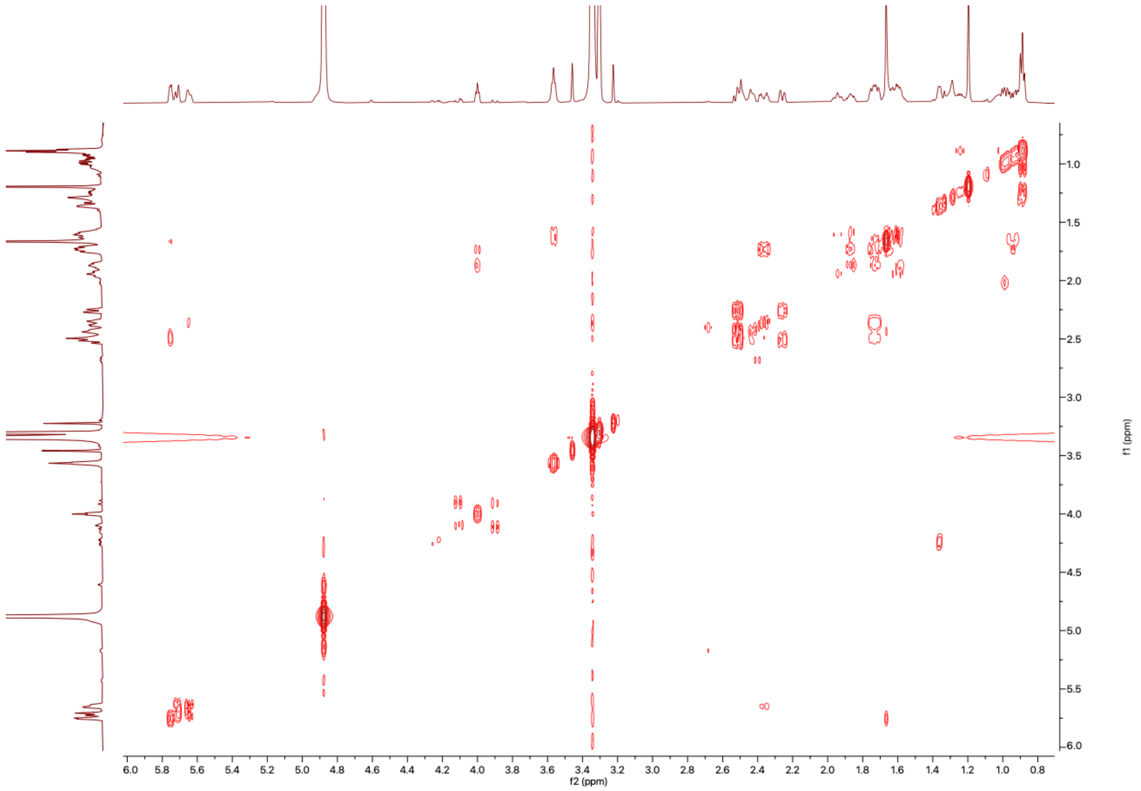

Figure S78: The ^{1}H-^{1}H COSY NMR Spectrum of **19** in MeOD (600 MHz)

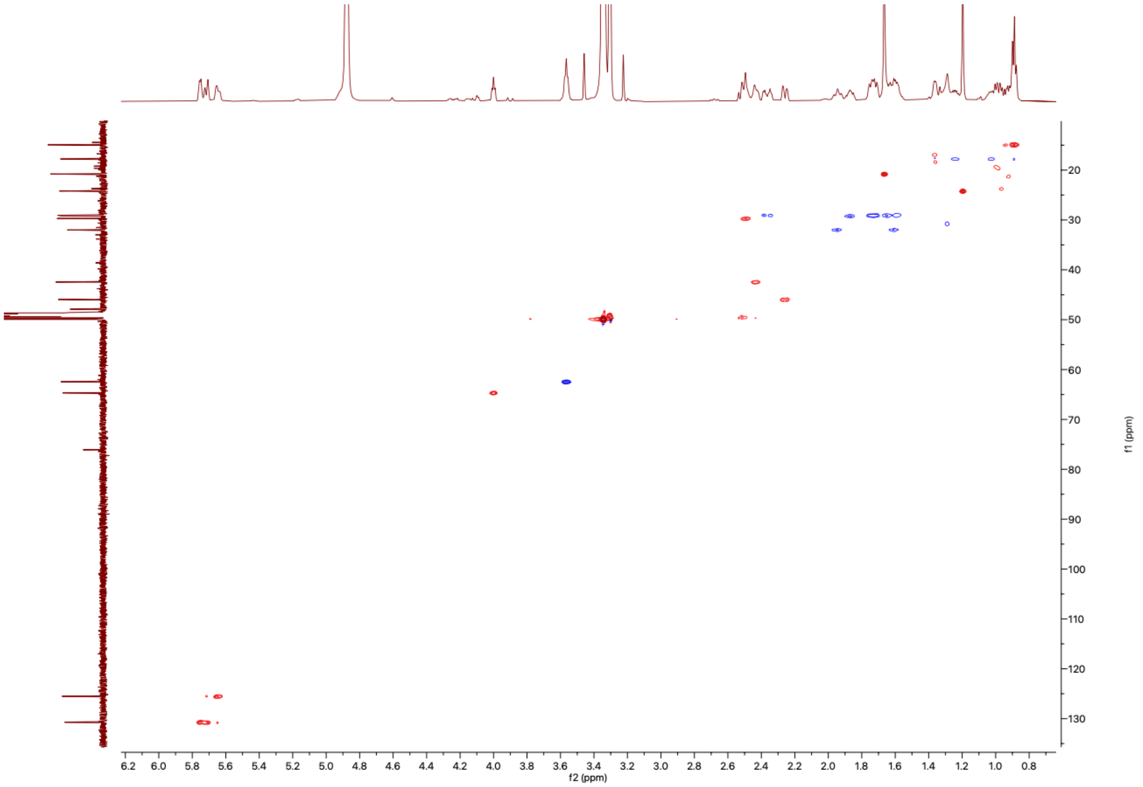

Figure S79: The ^{1}H-^{13}C HSQC NMR Spectrum of **19** in MeOD (600 MHz)

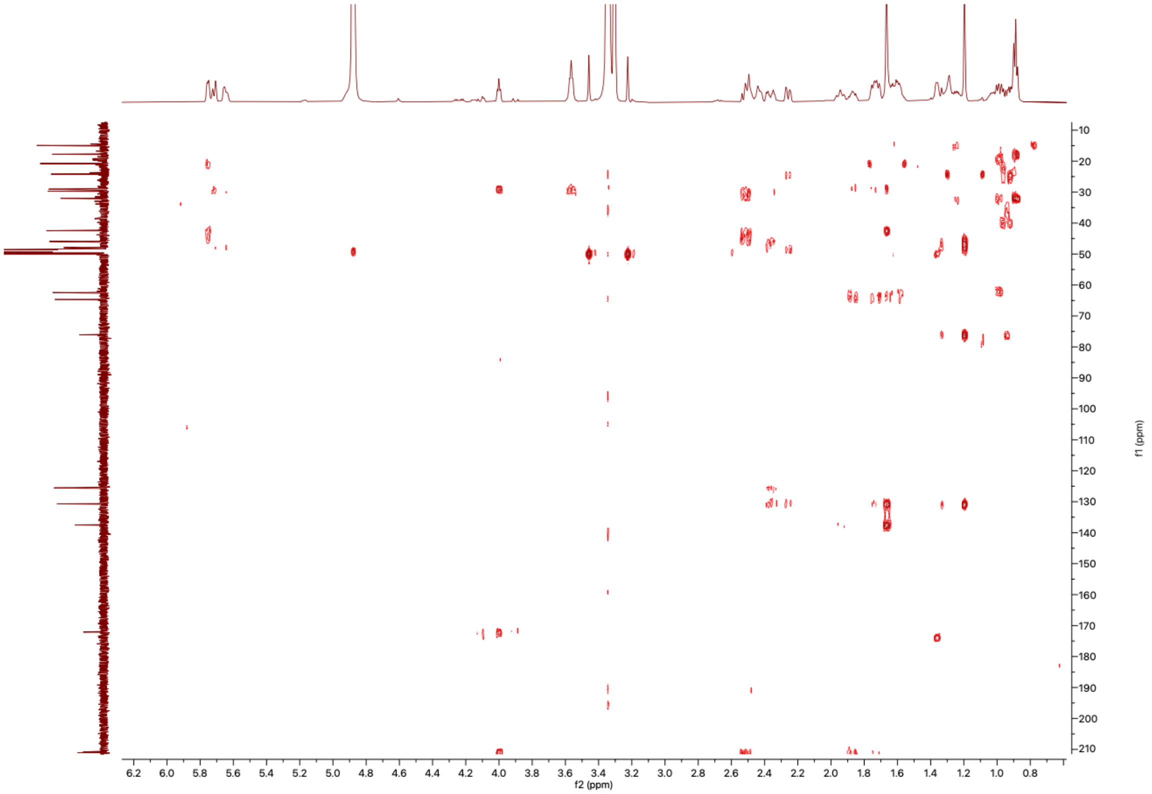

Figure S80: The ^{1}H-^{13}C HMBC NMR Spectrum of **19** in MeOD (600 MHz)

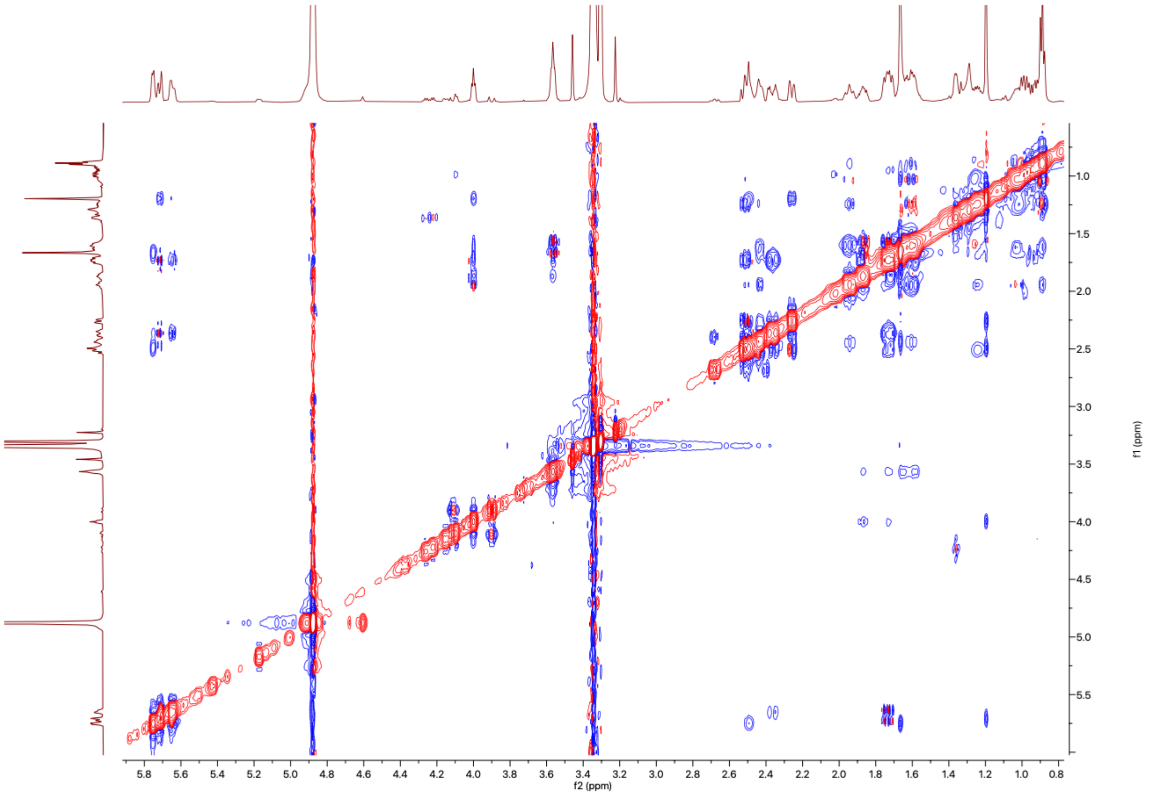

Figure S81: The ^{1}H-^{1}H NOESY NMR Spectrum of **19** in MeOD (600 MHz)

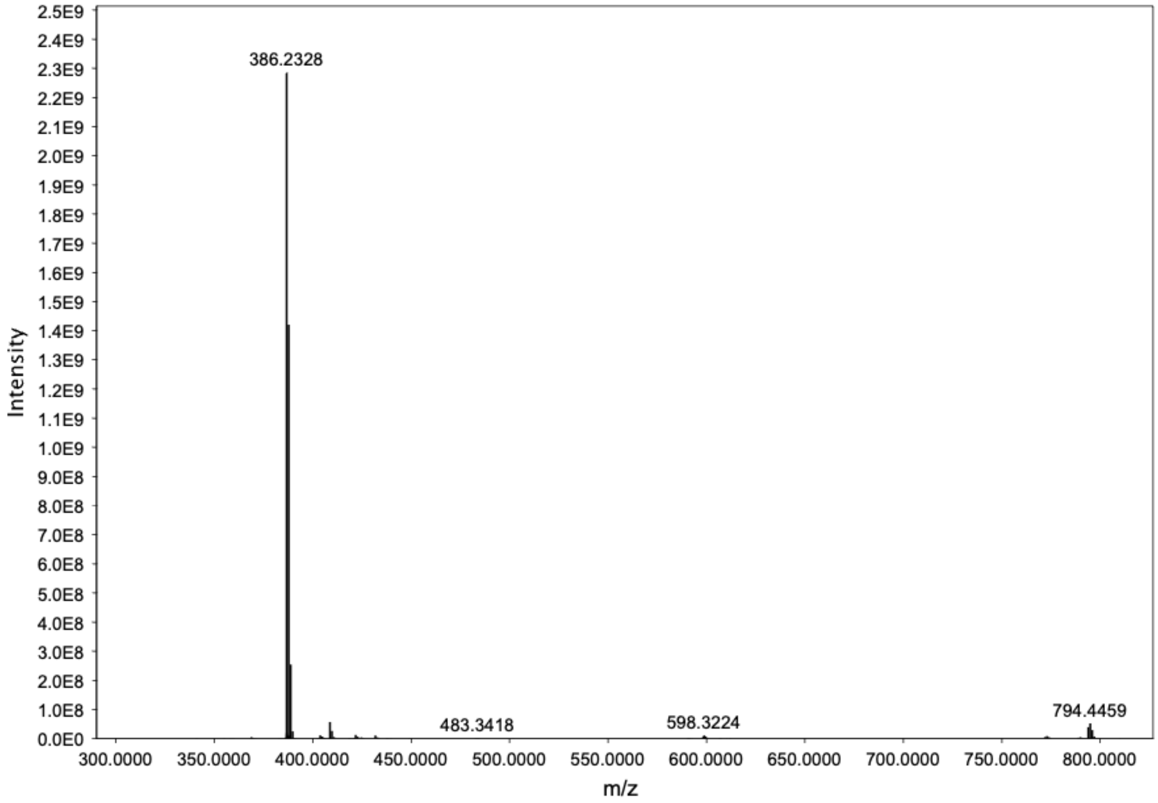

Figure S82: HRMS spectrum of **19**

References

(1) Chiang, Y.-M.; Lin, T.-S.; Chang, S.-L.; Ahn, G.; Wang, C. C. C. An Platform for the Complete Cluster Refactoring and Total Biosynthesis of Fungal Natural Products. *ACS Synth Biol* **2021**, *10* (1), 173–182.

(2) Szewczyk, E.; Nayak, T.; Oakley, C. E.; Edgerton, H.; Xiong, Y.; Taheri-Talesh, N.; Osmani, S. A.; Oakley, B. R. Fusion PCR and Gene Targeting in Aspergillus Nidulans. *Nat Protoc* **2006**, *1* (6), 3111–3120.

(3) Ralston, L.; Kwon, S. T.; Schoenbeck, M.; Ralston, J.; Schenk, D. J.; Coates, R. M.; Chappell, J. Cloning, Heterologous Expression, and Functional Characterization of 5-Epi-Aristolochene-1,3-Dihydroxylase from Tobacco (Nicotiana Tabacum). *Arch Biochem Biophys* **2001**, *393* (2), 222–235.

(4) Cacho, R. A.; Tang, Y. Reconstitution of Fungal Nonribosomal Peptide Synthetases in Yeast and In Vitro. *Methods Mol Biol* **2016**, *1401*, 103–119.

(5) Alexander, B. D. *Reference Method for Broth Dilution Antifungal Susceptibility Testing of Filamentous Fungi*; Clinical and Laboratory Standards Institute, 2017.

(6) Alexander, B. D. *Reference Method for Broth Dilution Antifungal Susceptibility Testing of Yeasts*; Clinical and Laboratory Standards Institute, 2017.

(7) Punjani, A.; Rubinstein, J. L.; Fleet, D. J.; Brubaker, M. A. cryoSPARC: Algorithms for Rapid Unsupervised Cryo-EM Structure Determination. *Nat Methods* **2017**, *14* (3), 290–296.

(8) Scheres, S. H. W. RELION: Implementation of a Bayesian Approach to Cryo-EM Structure Determination. *J Struct Biol* **2012**, *180* (3), 519–530.

(9) Waterhouse, A.; Bertoni, M.; Bienert, S.; Studer, G.; Tauriello, G.; Gumienny, R.; Heer, F. T.; de Beer, T. A. P.; Rempfer, C.; Bordoli, L.; Lepore, R.; Schwede, T. SWISS-MODEL: Homology Modelling of Protein Structures and Complexes. *Nucleic Acids Res* **2018**, *46* (W1), W296–W303.

(10) Guddat, L. W.; Lonhienne, T. Saccharomyces Cerevisiae Acetohydroxyacid Synthase. Worldwide Protein Data Bank February 15, 2017. https://doi.org/10.2210/pdb5ims/pdb.

(11) Pettersen, E. F.; Goddard, T. D.; Huang, C. C.; Couch, G. S.; Greenblatt, D. M.; Meng, E. C.; Ferrin, T. E. UCSF Chimera--a Visualization System for Exploratory Research and Analysis. *J Comput Chem* **2004**, *25* (13), 1605–1612.

(12) Emsley, P.; Lohkamp, B.; Scott, W. G.; Cowtan, K. Features and Development of Coot. *Acta Crystallogr D Biol Crystallogr* **2010**, *66* (Pt 4), 486–501.

(13) Afonine, P. V.; Klaholz, B. P.; Moriarty, N. W.; Poon, B. K.; Sobolev, O. V.; Terwilliger, T. C.; Adams, P. D.; Urzhumtsev, A. New Tools for the Analysis and Validation of Cryo-EM Maps and Atomic Models. *Acta Crystallogr D Struct Biol* **2018**, *74* (Pt 9), 814–840.

(14) Adams, P. D.; Afonine, P. V.; Bunkóczi, G.; Chen, V. B.; Davis, I. W.; Echols, N.; Headd, J. J.; Hung, L.-W.; Kapral, G. J.; Grosse-Kunstleve, R. W.; McCoy, A. J.; Moriarty, N. W.; Oeffner, R.; Read, R. J.; Richardson, D. C.; Richardson, J. S.; Terwilliger, T. C.; Zwart, P. H. PHENIX: A Comprehensive Python-Based System for Macromolecular Structure Solution. *Acta Crystallogr D Biol Crystallogr* **2010**, *66* (Pt 2), 213–221.

(15) *Website*. W. L. DeLano, “The PyMOL Molecular Graphics System,” DeLano Scientific LLC, Palo Alto, 2002. http://www.pymol.org.

(16) Pettersen, E. F.; Goddard, T. D.; Huang, C. C.; Meng, E. C.; Couch, G. S.; Croll, T. I.; Morris, J. H.; Ferrin, T. E. UCSF ChimeraX: Structure Visualization for Researchers, Educators, and Developers. *Protein Sci* **2021**, *30* (1), 70–82.

(17) Nierman, W. C.; Pain, A.; Anderson, M. J.; Wortman, J. R.; Kim, H. S.; Arroyo, J.; Berriman, M.; Abe, K.; Archer, D. B.; Bermejo, C.; Bennett, J.; Bowyer, P.; Chen, D.; Collins, M.; Coulsen, R.; Davies, R.; Dyer, P. S.; Farman, M.; Fedorova, N.; Fedorova, N.; Feldblyum, T. V.; Fischer, R.; Fosker, N.; Fraser, A.; García, J. L.; García, M. J.; Goble, A.; Goldman, G. H.; Gomi, K.; Griffith-Jones, S.; Gwilliam, R.; Haas, B.; Haas, H.; Harris, D.; Horiuchi, H.; Huang, J.; Humphray, S.; Jiménez, J.; Keller, N.; Khouri, H.; Kitamoto, K.; Kobayashi, T.; Konzack, S.; Kulkarni, R.; Kumagai, T.; Lafon, A.; Latgé, J.-P.; Li, W.; Lord, A.; Lu, C.; Majoros, W. H.; May, G. S.; Miller, B. L.; Mohamoud, Y.; Molina, M.; Monod, M.; Mouyna, I.; Mulligan, S.; Murphy, L.; O’Neil, S.; Paulsen, I.; Peñalva, M. A.; Pertea, M.; Price, C.; Pritchard, B. L.; Quail, M. A.; Rabbinowitsch, E.; Rawlins, N.; Rajandream, M.-A.; Reichard, U.; Renauld, H.; Robson, G. D.; Rodriguez de Córdoba, S.; Rodríguez-Peña, J. M.; Ronning, C. M.;

Rutter, S.; Salzberg, S. L.; Sanchez, M.; Sánchez-Ferrero, J. C.; Saunders, D.; Seeger, K.; Squares, R.; Squares, S.; Takeuchi, M.; Tekaia, F.; Turner, G.; Vazquez de Aldana, C. R.; Weidman, J.; White, O.; Woodward, J.; Yu, J.-H.; Fraser, C.; Galagan, J. E.; Asai, K.; Machida, M.; Hall, N.; Barrell, B.; Denning, D. W. Genomic Sequence of the Pathogenic and Allergenic Filamentous Fungus Aspergillus Fumigatus. *Nature* **2005**, *438* (7071), 1151–1156.

(18) Al Abdallah, Q.; Ge, W.; Fortwendel, J. R. A Simple and Universal System for Gene Manipulation in : -Assembled Cas9-Guide RNA Ribonucleoproteins Coupled with Microhomology Repair Templates. *mSphere* **2017**, *2* (6). https://doi.org/10.1128/mSphere.00446-17.

(19) Bok, J. W.; Keller, N. P. LaeA, a Regulator of Secondary Metabolism in Aspergillus Spp. *Eukaryot Cell* **2004**, *3* (2), 527–535.

(20) Shimizu, K.; Keller, N. P. Genetic Involvement of a cAMP-Dependent Protein Kinase in a G Protein Signaling Pathway Regulating Morphological and Chemical Transitions in Aspergillus Nidulans. *Genetics* **2001**, *157* (2), 591–600.

(21) Furukawa, T.; van Rhijn, N.; Chown, H.; Rhodes, J.; Alfuraiji, N.; Fortune-Grant, R.; Bignell, E.; Fisher, M. C.; Bromley, M. Exploring a Novel Genomic Safe-Haven Site in the Human Pathogenic Mould Aspergillus Fumigatus. *Fungal Genet Biol* **2022**, *161*, 103702.

(22) Kowalski, C. H.; Beattie, S. R.; Fuller, K. K.; McGurk, E. A.; Tang, Y.-W.; Hohl, T. M.; Obar, J. J.; Cramer, R. A., Jr. Heterogeneity among Isolates Reveals That Fitness in Low Oxygen Correlates with Aspergillus Fumigatus Virulence. *mBio* **2016**, *7* (5). https://doi.org/10.1128/mBio.01515-16.

(23) Li, H.; Barker, B. M.; Grahl, N.; Puttikamonkul, S.; Bell, J. D.; Craven, K. D.; Cramer, R. A., Jr. The Small GTPase RacA Mediates Intracellular Reactive Oxygen Species Production, Polarized Growth, and Virulence in the Human Fungal Pathogen Aspergillus Fumigatus. *Eukaryot Cell* **2011**, *10* (2), 174–186.

(24) Kerkaert, J. D.; Le Mauff, F.; Wucher, B. R.; Beattie, S. R.; Vesely, E. M.; Sheppard, D. C.; Nadell, C. D.; Cramer, R. A. An Alanine Aminotransferase Is Required for Biofilm-Specific Resistance of Aspergillus Fumigatus to Echinocandin Treatment. *mBio* **2022**, *13* (2), e0293321.

(25) National Research Council; Division on Earth and Life Studies; Institute for Laboratory Animal Research; Committee for the Update of the Guide for the Care and Use of Laboratory Animals. *Guide for the Care and Use of Laboratory Animals: Eighth Edition*; National Academies Press, 2011.
