# Supplementary material for: An Antifungal with a Novel Mechanism of Action Discovered via Resistance Gene-Guided Genome Mining
Source: ACS Cent Sci. 2026 Jan 31;12(2):197–207. doi: 10.1021/acscentsci.5c02019 (PMC12947280; doi:10.1021/acscentsci.5c02019)
Supplement: Supplementary file 3 [file oc5c02019_si_003.pdf]

oc-2025-02019a.R1

Name: Peer Review Information for "An antifungal with a novel mechanism of action discovered via resistance gene-guided genome mining"

First Round of Reviewer Comments

Reviewer: 1

Comments to the Author

The paper makes several important conceptual advances in the field. Firstly the study shows that genome mining can uncover novel antimicrobial agents, such as the spiro-cis-decalin tetramic acid identified here. A previous study did highlight that aspterric acid was identified by a similar approach. So while not completely new, as a concept it emphasises that there are many such compounds to be found by this approach..

Secondly, that this compound has activity against A. fumigatus is cell based assays represents a novel finding. This haas not been shown before to any great effect.

Solving the structure of the complex with A. fumigatus AHAS using cryo-EM represents a novel technological advancement as previously structural studies with AHAS have required crystallographic determination. The structures clearly reveal the mode of binding and show the covalent bonding to the lysine and the movement of nearby side-chains to lock the compound in place.

The gene knockout studies also provides new information. Though similar studies on C. neoformams and C albicans have been performed previously, it is important that the essential nature of the AHAS in Aspergillus be confirmed before major efforts are put into inhibitor discovery.

Somewhat unfortunate that the chemical structure is not stable in mouse plasma and microsomes, but this work represents a significant advance along the drug discovery pipeline.

I didn't find any significant issues with the manuscript so dont have any corrections to suggest.

Reviewer: 2

Comments to the Author

The manuscript entitled “An antifungal with a novel mechanism of action discovered via resistance gene-guided genome mining” by Perlatti et al is a very well written manuscript with detailed results, methods, and analysis. Overall, the paper works well and flows from section to section. I have some points of concern that the authors should address prior to publication but these are more clarifying then negative and so I believe once the corrections are made the manuscript will be ready for publication.

Major:

1. Pg. 4, ln 37-40. The statement “Consistent with this precedent, LCMS analysis of sdtE overexpression strains led to the identification of multiple features that were upregulated as compared to the wild-type strain, including a prominent feature with m/z = 386.233 (Figure 1E).” is not entirely accurate. In the figure shown, the authors show an EIC, they do not show an entire TIC that shows multiple features. This could be included in the SI. This leads to a second question of how the authors knew to do an EIC for 386.233? Was this the largest new peak seen in the TIC of the overexpressing mutant? Was this identified by metabolomics and a volcano plot? A little more detail would be appreciated by the reader.
2. In the SI the authors talk about “Untargeted metabolomics analysis” but not the processing or computational parameters that were used. These should be included and should include programs used, settings, etc.
3. Pg. 5, ln 47-48. This sentence states “Upon isolation, the metabolite responsible for this feature was determined to be HB-35018 (1, Table S5, Figures S11-S16), a unique spirocis-decalin containing tetramic acid.” This gives the implication that the stereochemistry and absolute configuration was determined by NMR. A few paragraphs later the authors state that the structure was confirmed by X-ray. I suggest moving that sentence to immediately following the sentence above.
    a. Also a reference to the section “Structure elucidation of isolated compounds” in the SI would be a good thing here.

4. There was no CIF provided for the X-ray structure as SI material.
5. In Figure S1Ci, the authors show the formation of compound **10** without sdtD, the Diels-

Alderase. Can they explain the formation of the Diels-Alder product without the enzyme?

6. Figure 2B legend. The authors state "Inhibition of wildtype (WT) and P192S mutants of ALS from S. cerevisiae by 1-3." But they show data for 1-5.
7. Figure 2D. It seems that the mechanism of ALS is slightly incorrect as it shows the formation of acetate not the TPP bound enamine intermediate.

Minor:

1. Pg. 1, ln 49. Italicize "Aspergillus terreus"
2. LCMS chromatograms are shown in seconds, which is unusual and are typically shown in minutes. Is there a reason why?
3. Pg. 8, ln 1. The sentence "1 exhibits broad antifungal activity via covalent, nonaccumulative inhibition of ALS" seems weirdly placed and is formatted differently than the text. Is it a title?
4. In the SI, Figure S5 and S4 are in mislabeled. In the text (Pg 9, ln 5 and ln, 17) they are referenced to what the labels should be in the SI.
5. Accurate HRMS spectrum for all compounds isolated should be included in the SI.
6. Figure 5. A title seems to be included. It should be removed.
7. Pg 13, ln 5. The "Center for disease control (CDC)" should be "Centers for Disease Control and Prevention"
8. In the SI, "To prepare biomass for transformation, Aspergillus pseudoterreus", the organism should be italicized.
9. In the SI, pg 6 has the statement "1 mL trace elements solution" I assume this is the "Trace elements concentrate" listed on pg 5?
10. SI, pg 7. "S. cerevisiae RC01 transformed with a plasmid expressing neoG" how was this plasmid created? What was the backbone?
11. SI, pg 8. "and a 6545 QTOF high resolution mass spectrometer" What were the MS settings?
12. SI, pg 10. "10^8 cells addedre to" I believe this is supposed to be "10^8 cells added to"

Reviewer: 3

Comments to the Author

Existing antifungal drugs- azoles, echinocandins, and polyenes- are old, face increasing resistance, and can be toxic, yet few new antifungals with novel mechanisms have been developed. The authors focus on finding new antifungal agents by mining fungal genomes,

specifically looking for biosynthetic gene clusters that contain resistance genes similar to essential fungal targets. One such pathway is the biosynthesis of branched-chain amino acids, where the enzyme acetolactate synthase (ALS) plays a key role. Using resistance gene–guided genome mining, the researchers discover a biosynthetic gene cluster, sdtBGC, that encodes a resistance gene homologous to ALS. They show that this cluster produces a natural product, HB-35018 (1), a structurally unique spiro–cis-decalin tetramic acid and a previously unknown ALS inhibitor. Biochemical studies demonstrate that HB-35018 strongly inhibits recombinant ALS from several fungal species. Structural analyses using intact protein mass spectrometry and cryo-electron microscopy reveal that (1) is a covalent inhibitor, binding ALS in a way distinct from known ALS inhibitors. The biochemical activity translates into activity in vitro in fungal inhibitory assays.

The authors demonstrate a natural product, produced by A. terreus, that has antifungal in vitro activity. Although the authors show in Figure 4 that has a MIC >32 μM, however, there is so data on the susceptibility of the A. terreus ALS enzyme (or Aspergillus nidulans which they used to produce 1). As the authors are proposing this target for antifungal development, some discussion, at least, of the likely resistance from some species, might be warranted.

Figure 4A. Would suggest labeling the 'IC50' values on the right side of panel 4A as EC50 values, as it looks like this is cellular inhibition data, for clarity. Also, it looks like the values listed might need verification. From the graph there is a trend, for 3- 2- 5 in regards to potency, however, the values listed for inhibition concentration are 3- 27uM; 2- 200uM; 5-90uM?

Figure 5 has the declarative statement "ALS is essential for pathogenicity of A. fumigatus." While the text below states "In vivo infection studies in a mouse model of invasive aspergillosis showed a significant increase in the survival of mice infected with an ilv2Δ strain (Figure 5A), demonstrating reduced virulence." Later in the text "These results demonstrate that ALS is essential for the pathogenicity of A. fumigatus" and in the discussion "ALS is essential to pathogenicity in a mouse model of invasive aspergillosis." As figure 5 demonstrates delayed pathogenicity, but still ~40% loss of survival in the ilv2Δ strain, the data does not support that ALS is essential to A. fumigatus pathogenicity. It is suggested the authors revise their declarative statements, based on the data. Some discussion of the impact, especially on the impacts of in vivo kinetics would be potentially warranted.

Would suggest removing or rephrasing- “Given the relatively recent evolutionary divergence of humans and fungi, potential targets meeting this criterion are rare.” Highlighting that both are eukaryotes and share similar processes/pathways would be more to the point, as it has been an estimated 1-1.5 billion years from the divergence of the common ancestor.

CLSI guidelines for Aspergillus are to read out at approx.. 48 hrs, why are only the 24 hr in vitro inhibitory values given (48 hr was also determined according to the methods), would suggest listing the CLSI recommended timepoint instead, or both values to allow interpretation.

Listed as [DNA] in text, legend and supplemental material, but also described as 18S DNA, shouldn’t this be 18S RNA?

In the mouse study, please describe the number of mice in each group (neither lung burden groups nor survival groups) are listed currently.

Author's Response to Peer Review Comments:

Colin Harvey
Hexagon Bio
1490 O'Brien Dr.
Menlo Park, CA
94041

Senior Editor
ACS Central Science

January 2, 2026

Editor,

I would like to take this opportunity to again thank you for taking the time to consider our manuscript for publication in ACS Central Science. We continue to believe that it is the ideal venue for the work.

I would also like to request you extend our sincere thanks to the reviewers you secured to consider our work. Their comments undoubtedly made our manuscript stronger. In the revised manuscript that has been resubmitted along with this letter, we have done our best to address the points raised by both reviewers and a point-by-point response with explicit call-outs to the alterations made in the manuscript is included here. A high level summary of the most significant alterations is as follows:

- Addition of multiple new Figures to the supporting information:
    - **Figure S1:** Demonstration that m/z = 386.233 is the most prominent of the upregulated features upon overexpression of *sdtE*.

- **Figure S9:** Multiple sequence alignment of multiple ALS protein sequences, attempting to potential justify the activity profile reported in **Figure 4**. These data are summarised in the newly added **Table S5**.
- HR-MS for all isolated compounds have also been added for all isolated molecules per a request of one of the reviewers.

As well as the above additional datasets, significant improvements have been made to the text of the manuscript to improve the clarity and accuracy. While we have attempted to address each suggestion and comment raised by each of the journal staff and reviewers 2 and 3 in the pages that follow, please don't hesitate to reach out for additional clarification or discussions of any of our responses.

Per the instructions we received we have uploaded our updated manuscript both as a Microsoft Word document with all changes from the original tracked as well as a clean PDF.

Thank you again for taking the time to consider our manuscript for publication in ACS Central Science..

All the best,

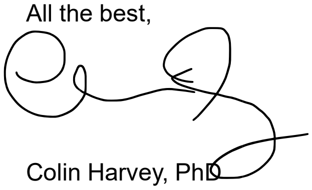

Colin Harvey, PhD
colin@hexagonbio.com
Co-founder & VP, Early Discovery
Hexagon Bio

Hexagon Bio, Inc., 1490 O'Brien Drive, Menlo Park, CA, 94025
+1-650-254-6044

Point-by-point responses

All reviewer comments and questions are retained verbatim from the received reviews. Please see the blue text for the author's responses.

Formatting Needs:

Supporting Information: The supporting information (SI) paragraph in the main file is incomplete. Please list each item (including graphics) that can be found in the SI-for-Publication file individually.

*Examples of sufficient descriptions: “Supporting Information: 1H NMR spectra for all compounds” or “Additional experimental details, materials, and methods, including photographs of experimental setup.”

*Examples of insufficient descriptions: “Supporting Information: Figures S1-S3” or “Additional figures as mentioned in the text.”

- We have updated the "Associated Content" portion of the manuscript to read as follows: "Supporting Information: Materials and methods; supporting data; and 1D NMR, 2D NMR, and HR-MS spectra for all isolated compounds.
  A CIF file containing the X-ray data collected for 1 is also provided."

Supporting Information: Please number all pages in the following format: S1, S2, S3, etc.

- Please see the updated SI

TOC Graphic: Include a TOC graphic illustrating the significance of the paper. The TOC graphic should be something that is representative of your entire work. Color schemes or illustrations typically make good choices. The TOC graphic must be original and free from any copyright issues. Confirm that all text is legible. Present the TOC graphic on the last page of the manuscript by itself. Please label the TOC as “TOC Graphic”. A caption describing the TOC is not needed.

Reviewer #1:

We thank reviewer #1 for taking the time to carefully read our manuscript. As they had no edits to suggest, we have no additional responses to include here.

Reviewer #2:

Major:

1. 1. Pg. 4, In 37-40. The statement “Consistent with this precedent, LCMS analysis of sdtE overexpression strains led to the identification of multiple features that were upregulated as compared to the wild-type strain, including a prominent feature with m/z = 386.233 (Figure 1E).” is not entirely accurate. In the figure shown, the authors show an EIC, they do not show an entire TIC that shows multiple features. This could be included in the SI. This leads to a second question of how the authors knew to do an EIC for 386.233? Was this the largest new peak seen in the TIC of the overexpressing mutant? Was this identified by metabolomics and a volcano plot? A little more detail would be appreciated by the reader
    a. We apologize for the lack of clarity here. The language in the manuscript has been updated as follows:
    "Consistent with this precedent, LCMS analysis of sdtE overexpression strains led to the identification of multiple features that were upregulated as compared to the wild-type strain, the most prominent of which was a peak with m/z = 386.233 (Figure 1E, Figure S1)."

We have added Figure S1 to the SI showing overlays of the BPCs for both the wild-type and sdtE overexpression strains as well as their associated EICs for m/z = 386.233 demonstrating that it was the most highly upregulated feature.

2. In the SI the authors talk about "Untargeted metabolomics analysis" but not the processing or computational parameters that were used. These should be included and should include programs used, settings, etc.
    a. We apologize for this omission. The following passage has been added to the 'Untargeted metabolomics analysis' section of the 'Materials and Methods': "Untargeted LCMS metabolomics data were analyzed using an internally developed, proprietary algorithm (FTOP; Hexagon Bio), which is not publicly available. The algorithm operates on a reference corpus comprising LC–MS features extracted from approximately 10,000 high-resolution LC–MS runs of *Aspergillus nidulans* chassis strains engineered for heterologous expression of ~400 distinct fungal BGCs. For each detected feature, defined by an m/z value and retention time, the algorithm estimates the likelihood that the feature originates from a specific target BGC (*sdt*BGC) based on its frequency of occurrence across the full dataset. Feature matching across runs was performed using an m/z tolerance of 5 ppm and a retention time tolerance of 0.03 min. Features that occur preferentially in samples expressing the *sdt*BGC relative to the broader dataset are assigned higher scores. While the full implementation of the algorithm is proprietary, the methodological framework described here enables evaluation of its application and limitations."
3. Pg. 5, ln 47-48. This sentence states "Upon isolation, the metabolite responsible for this feature was determined to be HB-35018 (1, Table S5, Figures S11-S16), a unique spiro-cis-decalin containing tetramic acid." This gives the implication that the stereochemistry and absolute configuration was determined by NMR. A few paragraphs later the authors state that the structure was confirmed by X-ray. I suggest moving that sentence to immediately following the sentence above. a. Also a reference to the section "Structure elucidation of isolated compounds" in the SI would be a good thing here.
    a. Please see the following sentence insert directly after the passage referenced above:
    "While the cis-stereochemistry of the decalin was determined by NMR using J-coupling constants and 2D-NOE correlations, X-ray crystallography was required to confirm the absolute stereochemistry (Figure S2, Supporting Data files) "
4. There was no CIF provided for the X-ray structure as SI material.
    a. A file containing these data has been included as supporting information and the deposited version will be unembargoed upon publication of the manuscript.
5. In Figure S1Ci, the authors show the formation of compound 10 without sdtD, the Diels-Alderase. Can they explain the formation of the Diels-Alder product without the enzyme?
    a. Spontaneous intramolecular Diels-Alder reactions of this type are precedented. We have added the following sentence to the manuscript to address this: "Upon formation of 9, a spontaneous intramolecular Diels-Alder reaction, analogous to that observed for intermediates in varicidin biosynthesis, results in partial conversion to a decalin (**Figure S1Ci**)"

6. Figure 2B legend. The authors state “Inhibition of wildtype (WT) and P192S mutants of ALS from S. cerevisiae by 1-3.” But they show data for 1-5.
    a. Thanks for catching this. It has been corrected
7. Figure 2D. It seems that the mechanism of ALS is slightly incorrect as it shows the formation of acetate not the TPP bound enamine intermediate.
    a. We thank the reviewer for identifying this error in the schematic representation of the ALS mechanism. We agree that the primary product of the first decarboxylation step is the TPP-bound hydroxyethyl intermediate, not free acetate.
    We have revised Figure 2D to accurately depict the formation of the hydroxyethyl-TPP enamine intermediate (HETPP) following the decarboxylation of the first pyruvate molecule. This intermediate is now shown to react with the second ketoacid, consistent with the established catalytic cycle of ALS.

Minor:

1. Pg. 1, ln 49. Italicize “Aspergillus terreus”
    a. Please see updated text.
2. LCMS chromatograms are shown in seconds, which is unusual and are typically shown in minutes. Is there a reason why?
    a. In order to better comply with the reviewers expectations, all chromatograms in Figure 1 and Figure S2 have been updated to show retention time in minutes.
3. Pg. 8, ln 1. The sentence “1 exhibits broad antifungal activity via covalent, non-accumulative inhibition of ALS” seems weirdly placed and is formatted differently than the text. Is it a title?
    a. This sentence is intended to be a subheading for a section of the results portion of the manuscript. It is similarly formatted to all other subheadings in the document and we will be sure to check the proofs to ensure that they are all correctly interpreted.
4. In the SI, Figure S5 and S4 are in mislabeled. In the text (Pg 9, ln 5 and ln, 17) they are referenced to what the labels should be in the SI.
    a. Apologies for the typo. This has been corrected.
5. Accurate HRMS spectrum for all compounds isolated should be included in the SI.
    a. HRMS spectra for each isolated compound have been added to the Supporting Figures, grouped by compound with the NMR spectra.
6. Figure 5. A title seems to be included. It should be removed.
    a. Apologies for the confusion, but I believe this is an artifact of figure placement. The title is meant to serve as a subheading for this section of the results. We will ensure that this is interpreted correctly at the proof stage.
7. Pg 13, ln 5. The “Center for disease control (CDC)” should be “Centers for Disease Control and Prevention”
    a. Please see updated text
8. In the SI, “To prepare biomass for transformation, Aspergillus pseudoterreus”, the organism should be italicized.

a. Please see updated text

9. In the SI, pg 6 has the statement “1 mL trace elements solution” I assume this is the “Trace elements concentrate” listed on pg 5?
    a. This assumption is correct. The language has been updated to be consistent.
10. SI, pg 7. “S. cerevisiae RC01 transformed with a plasmid expressing neoG” how was this plasmid created? What was the backbone?
    a. Apologies for the omission. A section describing the construction of this plasmid has been added to the methods.
11. SI, pg 8. “and a 6545 QTOF high resolution mass spectrometer” What were the MS settings?
    a. We apologize for this omission we have added the following text to the relevant method:
    "(Dual AJS ESI ion source - positive mode, Vcap voltage 3.5kV, nozzle voltage 1kV, gas and sheath gas temperature 350 °C, drying and sheath gas flow 11 L/min, nebulizer 60 psig. MS TOF - fragmentor, skimmer and Oct 1 RF Vpp voltages 175, 65 and 750 V respectively, *m/z* range 100-3000 *m/z* at 1 spectra/s, 9949 transients/spectrum, resolution 40.000 at *m/z* 2722)"
12. SI, pg 10. “108 cells addedre to” I believe this is supposed to be “108 cells added to”
    a. This is correct. Please see updated text

Reviewer #3:

1. The authors demonstrate a natural product, produced by A. terreus, that has antifungal in vitro activity. Although the authors show in Figure 4 that has a MIC >32 μM, however, there is so data on the susceptibility of the A. terreus ALS enzyme (or Aspergillus nidulans which they used to produce 1). As the authors are proposing this target for antifungal development, some discussion, at least, of the likely resistance from some species, might be warranted.
    a. We appreciate the suggestion. We have added a multiple sequence alignment of ALS sequences in **Figure S9** and a summary of key residues in **Table S3**. We have added the following passage referencing these results to the discussion section of the manuscript:
    "The spectrum of antifungal activity of 1 is also notable. When considered alongside our forward genetic screens, a potential trend emerges: among Aspergillus and Fusarium species, sensitivity to 1 correlates with the identity of residue Pro192 (based on the S. cerevisiae sequence), the site most frequently mutated in our screens. Species sensitive to 1 retain a proline at this position, whereas resistant species carry a valine or alanine (Table S3, Figure S9). Notably, this residue is a valine in the ALS of A. nidulans and an alanine both in SdtC and the ALS of A. terreus, providing a potential partial explanation for the lack of host toxicity during both native and heterologous production of 1."
2. Figure 4A. Would suggest labeling the ‘IC50’ values on the right side of panel 4A as EC50 values, as it looks like this is cellular inhibition data, for clarity. Also, it looks like the values listed might need verification. From the graph there is a trend, for 3- 2- 5 in regards to potency, however, the values listed for inhibition concentration are 3- 27uM; 2- 200uM; 5- 90uM?

a. We appreciate the careful inspection of the IC50 values. We have corrected the listed values and included an updated Figure 4 in the revised manuscript. As the data listed are calculated based inhibitory data. Additionally we have taken the suggestion to relabel these values as EC50s rather than IC50s in the updated version of Figure 4A.

3. Figure 5 has the declarative statement “ALS is essential for pathogenicity of A. fumigatus.” While the text below states “In vivo infection studies in a mouse model of invasive aspergillosis showed a significant increase in the survival of mice infected with an ilv2Δ strain (Figure 5A), demonstrating reduced virulence.” Later in the text “These results demonstrate that ALS is essential for the pathogenicity of A. fumigatus” and in the discussion “ALS is essential to pathogenicity in a mouse model of invasive aspergillosis.” As figure 5 demonstrates delayed pathogenicity, but still ~40% loss of survival in the ilv2Δ strain, the data does not support that ALS is essential to A. fumigatus pathogenicity. It is suggested the authors revise their declarative statements, based on the data. Some discussion of the impact, especially on the impacts of in vivo kinetics would be potentially warranted.
    a. We appreciate the suggestion. All references to ILV2 being 'essential to pathogenicity' have been updated to 'required for full virulence'
4. Would suggest removing or rephrasing- “Given the relatively recent evolutionary divergence of humans and fungi, potential targets meeting this criterion are rare.” Highlighting that both are eukaryotes and share similar processes/pathways would be more to the point, as it has been an estimated 1-1.5 billion years from the divergence of the common ancestor.
    a. We have updated this sentence to read as follows:
       "Given the high level of conservation between humans and fungi at both the protein and pathway levelsrelatively recent evolutionary divergence of humans and fungi, potential targets meeting this criterion are rare"
5. CLSI guidelines for Aspergillus are to read out at approx.. 48 hrs, why are only the 24 hr in vitro inhibitory values given (48 hr was also determined according to the methods), would suggest listing the CLSI recommended timepoint instead, or both values to allow interpretation.
    a. We have included these data alongside the 24hr data in Table S5. Additionally, we have added the following sentence to the discussion of the PK parameters of 1:
       "Consistent with these assumptions, we see a significant decrease in the potency of antifungal activity after 48 hr incubations (Table S5)."
6. Listed as [DNA] in text, legend and supplemental material, but also described as 18S DNA, shouldn’t this be 18S RNA?
    a. We apologize for any confusion. The assay is, in fact, amplifying DNA for the gene for the 18S rRNA. We have attempted to clarify this by updating the text as follows:
       "To determine the fungal burden in the lungs, the mice were sacrificed 72 hours after inoculation. The lungs were harvested, flash frozen, and the relative fungal burden was assessed through qPCR quantitation of *A. fumigatus* 18S rDNA (the gene for the 18S rRNA), as previously described."

7. In the mouse study, please describe the number of mice in each group (neither lung burden groups nor survival groups) are listed currently.
    a. Rather than having these data exist solely in caption to Figure 5, we have also included them in main text alongside references to Figure 5A and Figure 5B.
